# Joint Three-Dimensional Path Planning for Multiple Unmanned Aerial Vehicles in Static Environments Using an Improved Whale Optimization Algorithm

**DOI:** 10.3390/biomimetics11100685

**Published:** 2026-09-22

**Authors:** Yizhou Wang, Simon Liu

**Affiliations:** School of Electrical and Electronic Engineering, Nanyang Technological University, Singapore 639798, Singapore; simon.liu@ntu.edu.sg

**Keywords:** multiple unmanned aerial vehicles, three-dimensional coordinated path planning, improved whale optimization algorithm, spherical-increment encoding

## Abstract

Coordinated multi-unmanned aerial vehicles (UAVs) planning requires the joint consideration of spatial paths, flight speeds, arrival times, and separation constraints. This study develops an improved whale optimization algorithm (IWOA) for offline path–speed planning in known static environments. The method combines continuous spherical-increment encoding with deterministic initialization, nonlinear adaptive weighting, population migration, and a heavy-tailed candidate. Among eight algorithms, IWOA achieved the best mean rank of 1.38 on 29 2017 IEEE Congress on Evolutionary Computation (CEC2017) functions and a mean rank of 1.25 across four repeated single-UAV scenarios. Eight algorithms were also evaluated in four 230-dimensional, five-UAV scenarios using 30 runs, a population of 30, and 500 iterations, for 960 runs in total. The penalty-only IWOA obtained the lowest median final fitness in three scenarios, but no solution passed the post hoc feasibility screen. We therefore introduced a feasibility-priority variant with corrected angular and geometric checks, normalized violation magnitudes, continuous-time separation checks, and Deb-style ordering. In a separate 240-run comparison, Improved Whale Optimization Algorithm with Feasibility-Priority (IWOA-FP) and Whale Optimization Algorithm with Feasibility-Priority (WOA-FP) each returned 120/120 modeled-feasible terminal incumbents from a shared, prevalidated feasible warm start. Conditional objective comparisons favored IWOA-FP in Map 4, Complexity Setting 1 (M4-C1) and Map 4, Complexity Setting 2 (M4-C2) and WOA-FP in Map 3, Complexity Setting 2 (M3-C2), with neither method favored in Map 3, Complexity Setting 1 (M3-C1). These results show that IWOA is competitive under fixed-generation testing and that explicit feasibility-priority selection can preserve and optimize a feasible plan. Validation remains limited to the tested static model, and neither measures unseeded feasibility discovery nor certifies flight safety.

## 1. Introduction

Advances in flight control, navigation and positioning, wireless communication, and intelligent sensing have expanded the use of unmanned aerial vehicles (UAVs). Applications include terrain mapping, disaster rescue, forest and power-line inspection, target monitoring, and logistics transportation [1]. Path planning is essential to autonomous UAV flight and mission execution. Given the start and target points and environmental information, a planner seeks a flight path that balances safety, executability, and mission efficiency [2]. Unlike two-dimensional mobile robots, UAVs must adjust their horizontal position and altitude in three-dimensional space while satisfying terrain, obstacle, threat-region, no-fly-zone, and flight-attitude constraints [3]. Multi-UAV systems must also coordinate the path, speed, and arrival time of each UAV to prevent conflicts during mission execution. Three-dimensional cooperative path planning has therefore become an important problem in autonomous UAV navigation.

Several representative approaches have been developed for UAV path planning [2]. Graph-search methods are conceptually clear and yield interpretable routes, but their time and memory requirements increase rapidly with map scale and three-dimensional discretization resolution [4]. Artificial-potential-field methods provide rapid local obstacle avoidance, although local minima and oscillations remain problematic in narrow regions [5,6]. Random-sampling methods can rapidly identify feasible paths in continuous spaces, but the resulting stochastic paths generally require refinement. Metaheuristics require no explicit gradients and can flexibly incorporate nonlinear costs and constraints. UAV applications include genetic algorithms [7,8], differential evolution [9], gray wolf optimization [10,11], WOA [12,13], bat algorithms [14,15], the golden eagle optimizer [16], and chimp optimization [17,18]. Broad surveys describe the trade-offs among computational cost, path quality, and environmental adaptability [1]. Recent reviews further emphasize that multi-UAV planning requires the explicit treatment of coordination, scalability, and safety validation, rather than merely aggregating single-UAV routes [19].

This study focuses on offline three-dimensional mission preplanning for multiple UAVs in known static environments. Optimizing only the spatial geometry of multi-UAV paths is generally insufficient to establish that the overall mission plan is executable [1,3]. The planner must also consider terrain and no-fly-zone avoidance, threat exposure, turning and pitch angles, minimum segment length, task delay, and inter-UAV separation. In cooperative missions, flight speed affects both mission completion time and when each UAV enters a potential conflict region. Spatial path planning and temporal coordination are therefore interdependent. Accordingly, this study jointly optimizes three-dimensional UAV paths and flight speeds using an implemented composite objective, while evaluating complete safety and mission feasibility separately.

This problem presents several challenges. First, as the numbers of UAVs and intermediate waypoints increase, the dimension of the joint decision vector comprising spatial paths and flight speeds grows rapidly. The resulting optimization problem is high-dimensional, nonlinear, and multimodal, which substantially increases the difficulty of global search. Second, path length, safety risk, flight attitude, and mission time are strongly coupled and mutually constraining. Simply shortening a path may increase the risk of entering a threat region or violating attitude constraints, whereas excessive detours may increase flight time and cause task delay. A single performance measure is therefore insufficient to obtain a plan that balances safety and mission efficiency. Finally, multiple UAVs exhibit a more complex spatiotemporal coupling. Intersecting spatial paths do not necessarily imply a collision; a conflict can be determined accurately only by considering the times at which the UAVs reach the potential intersection region. Planning each spatial path independently is therefore unlikely to yield a globally coordinated mission plan, and spatial paths and temporal information must be considered within a unified optimization framework.

Metaheuristic optimization offers a flexible way to solve this high-dimensional, multi-constraint, strongly coupled problem. Whale Optimization Algorithm (WOA) [12,13] is a representative swarm-intelligence algorithm that performs global optimization by simulating humpback whales’ prey encirclement, spiral bubble-net attacks, and random search. Its simple structure, small number of parameters, and ease of implementation make it suitable for continuous-space optimization. However, on high-dimensional, multimodal, strongly constrained problems, the original WOA may suffer from inadequate initial population coverage, excessive dependence on the current best individual, and declining population diversity in later iterations, which can impair global search and convergence stability. Random initialization may distribute a limited population unevenly in a high-dimensional search space. Moreover, because position updates depend strongly on the current best individual, the population can cluster rapidly and converge prematurely if that solution lies in a local optimum. Population diversity and effective step sizes also decrease during the iterations, making it difficult to balance local search accuracy with the ability to escape local optima. These limitations are particularly pronounced in multi-UAV cooperative path planning, where the decision dimension and constraint relationships are more complex.

This study addresses these issues at the modeling and optimization levels. First, it establishes a joint three-dimensional path–speed optimization model for multiple UAVs. A unified continuous encoding represents both spatial trajectories and flight speeds, allowing inter-UAV conflicts to be evaluated from actual arrival times. Second, it develops a multi-strategy improvement framework based on WOA to strengthen global optimization in high-dimensional complex search spaces by improving initial population distribution, regulating the search stages, and preserving population diversity. Finally, the proposed method is evaluated using 2017 IEEE Congress on Evolutionary Computation (CEC2017) benchmark tests and multi-scenario UAV path-planning experiments.

This study makes the following contributions:

1. We establish a joint three-dimensional path–speed planning formulation for multiple UAVs. The continuous optimization framework combines the three-dimensional path parameters and flight speeds of all UAVs to couple spatial trajectories with temporal coordination. The formulation considers terrain and no-fly-zone avoidance, threat risk, turning and pitch angles, minimum segment length, task delay, and inter-UAV separation. It also uses waypoint arrival times in the implemented pairwise-separation penalty. With spherical-increment path parameterization, radial step lengths, elevation angles, azimuth angles, and flight speeds form a continuous joint decision vector. The experimental evaluator represents these requirements through weighted surrogate and sampled penalty terms; complete compliance is not inferred from the composite score.
2. We evaluate an integrated multi-strategy whale optimizer for high-dimensional joint planning. Its principal mechanisms are deterministic coordinate-wise initialization, nonlinear adaptive weighting, population migration, and a heavy-tailed Cauchy candidate. Section 4 documents the executable routine and its retained selection and diversity mechanisms. The controlled factor screen evaluates the four named mechanisms on a common modular WOA backbone. We therefore attribute performance to the evaluated integrated procedure and report factor-level associations without claiming that any single operator is independently novel or causally dominant.
3. We use a multilevel evaluation scheme to assess numerical optimization and simulated path-planning performance. Improved Whale Optimization Algorithm (IWOA) is evaluated on the CEC2017 benchmark functions, in repeated single-UAV experiments, and across 960 legacy penalty-only cooperative runs. A controlled six-variant modular WOA-backbone factor screen complements a 240-run feasibility-priority comparison between Improved Whale Optimization Algorithm with Feasibility-Priority (IWOA-FP) and Whale Optimization Algorithm with Feasibility-Priority (WOA-FP). This comparison uses a common validated feasible incumbent, normalized violation magnitudes, corrected geometry, and Deb-style ordering. It reports both the modeled-feasibility proportion and the implemented composite objective conditional on feasibility. We keep these result families separate to distinguish feasibility preservation from random-start discovery and complete flight-safety certification.

## 2. Related Work

### 2.1. Three-Dimensional UAV Path-Planning Methods

Three-dimensional UAV path-planning methods can be broadly classified into discrete search, local reactive planning, random sampling, and continuous optimization [1]. Discrete-search methods identify feasible paths by discretizing three-dimensional space into grids or graphs. Continuous-optimization methods instead optimize waypoints or path parameters directly in a continuous decision space.

Grid-based methods, including A*, Theta*, and Jump Point Search, are interpretable and can identify low-cost paths on discrete models using heuristic functions. However, their time and space requirements are sensitive to map scale and resolution. Artificial potential field (APF) and dynamic window approaches use local geometry to generate rapid obstacle-avoidance maneuvers, but they can become trapped in local minima or oscillate in narrow passages. Huang and Chen integrated the dynamic window approach with APF for three-dimensional UAV path planning [5]. Han et al. combined global GWO search with APF-based local path refinement to improve safety and planning efficiency in post-disaster rescue [6]. Zhao et al. developed a three-dimensional global planner for UAV swarms by combining Theta* with APF [20]. These hybrid methods reduce the limitations of individual planning mechanisms, although their performance remains sensitive to environment discretization, heuristic functions, and local rules.

Continuous-optimization methods use waypoints or parameterized curves as decision variables and evaluate path length, threat exposure, smoothness, and constraint violations through a unified objective function. Tu et al. proposed Differential Evolution-based Constrained Multi-objective Path-planning Algorithm (DECMPA), which integrates differential evolution and quasi-oppositional learning into the marine predators algorithm and adopts a spherical-coordinate vector for three-dimensional navigation [21]. Their results indicate that encoding and search mechanisms jointly affect the feasible-solution rate and optimization efficiency. Population-based optimization is therefore suitable for continuous, multi-constraint planning in complex static environments. For a single UAV, the primary challenges are environmental avoidance and trajectory executability. Multi-UAV planning additionally requires temporal coordination along the modeled paths, and therefore cannot be treated as a simple superposition of independent single-UAV problems.

### 2.2. Cooperative Path Planning for Multiple UAVs

In addition to terrain, obstacle, and flight-attitude constraints, cooperative multi-UAV planning must account for interactions among aircraft [2,3]. Spatial-path intersections alone cannot identify inter-UAV conflicts. Two UAVs may safely traverse the same region at different times, whereas temporal overlap in a shared region can create a conflict [22,23]. Explicit position–time modeling is therefore essential for distinguishing geometrically feasible paths from executable cooperative plans.

One research direction extends single-UAV graph search with conflict detection and constraint trees. Liu et al. proposed Multi-Conflict-Based Search (MCBS), which uses a refined A* method to enforce UAV flight constraints at the lower level and resolves spatiotemporal conflicts at the higher level [22]. Lu et al. generated initial paths using three-dimensional Jump Point Search and replanned selected segments through two-agent conflict search and incremental updates [4]. These methods preserve the interpretability of graph search and can produce explicit conflict-free path sets. However, as the numbers of UAVs, discrete nodes, and conflicts increase, high-level constraint combinations and repeated searches can impose substantial computational costs.

Another research direction reduces the dimension of the joint search through problem decomposition and cooperative coevolution. Wu et al. divided multi-UAV paths among subpopulations, used a separable representation with shared individuals, and incorporated flight, spatial, and temporal coordination into a common cost function [23]. Their model constructs a time horizon for each UAV from path length and speed bounds and evaluates temporal overlap at path intersections. Meng et al. further proposed a cooperative evolutionary method with adaptive decision-variable selection, which concentrates computational resources on variables associated with infeasible or highly conflicted UAVs [24]. Zhou et al. used a two-stage, knowledge-assisted coevolutionary NSGA-II for bi-objective multi-UAV path planning [25]. These studies show that decomposition, information sharing, and multiobjective selection can mitigate dimensional growth, although performance remains sensitive to subproblem definitions, representative selection, and constraint-handling strategies.

Search-based methods generally detect and resolve conflicts among previously generated paths, whereas evolutionary methods emphasize joint optimization of multiple paths [4,22,23,24]. For offline missions involving delay, threat avoidance, attitude constraints, and safety separation, assigning speeds only after path generation prevents spatial and temporal decisions from interacting during the search. Sequential path planning and speed allocation may therefore limit spatiotemporal coordination. Here, the three-dimensional trajectory and flight speed of each UAV are encoded jointly. Path geometry, flight time, and inter-UAV conflicts can consequently be adjusted within a unified fitness evaluation. The resulting framework supports global spatiotemporal preplanning in known static environments, rather than online avoidance or task allocation.

### 2.3. Metaheuristic Path-Planning Methods

Metaheuristic optimization algorithms generally require no explicit gradient information and are highly adaptable to nonconvex and nondifferentiable optimization models with complex penalty terms; they are therefore widely used in UAV path planning [1,19]. Genetic algorithms, differential evolution, particle swarm optimization, gray wolf optimization, and WOA typically represent candidate paths as population individuals and progressively reduce the composite cost through selection, mutation, individual interaction, or collective cooperation. Their advantages include flexible model adaptation and the ease of incorporating constraints related to collisions, space and time, threats, and energy consumption [19]. Their main limitation is the difficulty of ensuring both global exploration and local exploitation under a limited computational budget; performance also varies with initialization, path encoding, parameter settings, and penalty design.

Recent studies have addressed these limitations by combining complementary search strategies. Han et al. coupled GWO with APF to guide local safety adjustments using global search results [6]. Tu et al. combined quasi-oppositional learning, differential evolution, and greedy selection in DECMPA to improve population coverage, convergence, and escape from local optima [21]. Other studies have introduced Latin hypercube sampling, a golden-sine strategy, and adaptive weights [26], or combined chaotic initialization, Levy-Metropolis search, SA-PSO, and elite mutation [27]. Yu and Luo developed a reinforcement-learning-based multi-strategy cuckoo search method that selects complementary search operators adaptively and evaluated it on both CEC2017 functions and three-dimensional UAV planning tasks [28]. These methods reflect three common directions: improving initial search-space coverage, dynamically balancing exploration and exploitation, and restoring population diversity through perturbation, crossover, or local planning. Because multi-strategy designs can contain overlapping mechanisms, their contributions should be assessed using benchmark functions and repeated experiments.

WOA updates candidate solutions in continuous space by simulating prey encirclement, spiral bubble-net attacks, and random search [29]. For UAV path planning, Li et al. used opposition-based learning, random convergence factors, and neighborhood perturbations to improve initial diversity and escape from local optima in small-swarm trajectory planning [12]. Yang et al. combined opposition-based learning, a nonlinear convergence factor, and a random-number generation mechanism to improve the convergence accuracy and stability of three-dimensional UAV trajectory planning [13]. Collectively, these studies show that WOA can be extended to continuous, multi-constraint, and multiobjective path optimization. Initialization quality, use of leader information, and population diversity are recurring design considerations.

WOA variants have used enhanced initialization, parameter adaptation, and mutation-based perturbation to improve numerical optimization [30]. The distinction of the present study is therefore the integration of established search mechanisms with one high-dimensional spatiotemporal decision vector, rather than a claim that each operator is individually new. Relative to sequential path and speed allocation, the joint encoding allows geometry, travel time, and pairwise proximity to influence every fitness evaluation. Relative to the cooperative evolutionary methods summarized in Section 2.2, the complete mission plan is optimized in one continuous vector and evaluated with an explicit feasibility-priority branch. The experiments consequently test an integrated offline planning procedure and the practical effect of feasibility-first selection under the stated simulation conditions.

## 3. Three-Dimensional Cooperative Path-Planning Model for Multiple UAVs

### 3.1. Model Overview

Three-dimensional cooperative path planning determines flight paths for multiple UAVs between specified start and target points in a known mission environment. The paths must satisfy spatial boundaries, threat-avoidance requirements, flight-performance limits, arrival-time requirements, and inter-UAV separation while minimizing the composite mission cost. Unlike single-UAV planning, cooperative planning must balance the length and safety of each path while accounting for spatiotemporal coupling among UAVs. Changing the waypoints, speed, or departure time of one UAV also changes its arrival times and separation from other UAVs. The resulting problem is high-dimensional, nonlinear, strongly constrained, and multimodal.

Building on common three-dimensional path-planning costs, including path length, threat exposure, altitude, and smoothness, this study adds minimum-segment, mission-delay, and inter-UAV separation requirements. Section 3 first defines the problem and its assumptions and then models the terrain, threat regions, and no-fly zones. A spherical-increment encoding is subsequently introduced. Finally, the path-length, threat, turning-angle, minimum-segment, delay, and sampled inter-UAV separation costs are combined into the objective function for cooperative three-dimensional path planning.

### 3.2. Problem Description and Basic Assumptions

#### 3.2.1. Problem Description

Consider $N_{u}$ UAVs in the mission space. The start and target points of UAV *i* are defined as

(1) $$S_{i}=\left(x_{i}^{s},y_{i}^{s},z_{i}^{s}\right)$$

(2) $$G_{i}=\left(x_{i}^{g},y_{i}^{g},z_{i}^{g}\right)$$

where $i=1,2,\ldots ,N_{u}$ identifies the UAV.

Let $n_{i}$ denote the number of intermediate waypoints between the start and target points. The complete path of the *i*th UAV is expressed as

(3) $$P_{i}=\left\{P_{i,0},P_{i,1},\ldots ,P_{i,n_{i}},P_{i,n_{i}+1}\right\}$$

where

(4) $$P_{i,0}=S_{i},\;P_{i,n_{i}+1}=G_{i}$$

Each intermediate waypoint is

(5) $$P_{i,k}=\left(x_{i,k},y_{i,k},z_{i,k}\right),\;k=1,2,\ldots ,n_{i}$$

These coordinates are the intermediate waypoints to be optimized.

The paths of all UAVs are assembled as

(6) $$P=\left\{P_{1},P_{2},\ldots ,P_{N_{u}}\right\}$$

The objective of multi-UAV path planning is to identify the optimal joint decision vector $X^{*}$, which minimizes the composite objective function while satisfying all modeled constraints:

(7) $$X^{*}=\arg\min_{X\in \Omega }F\left(X\right)$$

The feasible set is defined by constraints on spatial boundaries, speed, turning angles, threat avoidance, minimum segment length, arrival time, and inter-UAV safety separation.

#### 3.2.2. Basic Assumptions

The following assumptions isolate the path-planning problem and control model complexity. First, planning is completed before mission execution, and the terrain, radar threats, other threats, and no-fly zones are known. The study therefore addresses offline planning in static environments. Second, each UAV has a specified start point, target point, departure time, and expected arrival time, and it follows its planned waypoints to the target. Third, each UAV maintains a constant mission speed, and brief acceleration transients are ignored. Speeds may differ among UAVs and are optimized as cooperative planning variables. Fourth, each UAV follows straight segments between adjacent waypoints. B-spline smoothing is applied to the resulting piecewise-linear trajectory only for visualization. Fifth, the model excludes sudden airflow, communication interruptions, positioning errors, and other random disturbances. Threat-region safety radii are assumed to include the UAV dimensions and required safety margin. Sixth, each UAV pair must maintain at least the prescribed separation distance. Inter-UAV conflicts are evaluated from both position and traversal time, rather than from spatial intersections alone.

### 3.3. Three-Dimensional Mission-Environment Modeling

#### 3.3.1. Mission Space and Terrain Model

The three-dimensional mission space is defined as

(8) $$\Omega_{\mathrm{map}}=\left\{\left(x,y,z\right)|x_{\min}\leq x\leq x_{\max},\;y_{\min}\leq y\leq y_{\max},\;z_{\min}\leq z\leq z_{\max}\right\}$$

Terrain is represented by a discrete elevation matrix:

(9) $$H=\left[H(x,y)\right]$$

where H(x,y) is the terrain elevation at horizontal coordinates (x,y).

Two maps with different spatial scales are used. The first covers approximately 1045×879 units, and the second covers approximately 450×450 units. Different complexity settings yield four scenarios: M1-C1, M1-C2, M2-C1, and M2-C2. The cooperative multi-UAV experiments use five start–target pairs and 15 intermediate waypoints per UAV. Given the required terrain-clearance margin $h_{\mathrm{safe}}$, each planned waypoint must satisfy

(10) $$z_{i,k}\geq H\left(x_{i,k},y_{i,k}\right)+h_{\mathrm{safe}}$$

Equation (3) defines the intended terrain-clearance requirement. In the reported MATLAB(R2018a) experiments, however, the elevation matrix is used to construct and visualize the three-dimensional environment, whereas the evaluated objective uses an absolute altitude-band surrogate and does not query the DEM at each waypoint. The statistical export directly reports counts for radar and other threats, turning angles, minimum segment lengths, and sampled inter-UAV proximity. Consequently, neither the objective value nor the plotted trajectory independently verifies DEM clearance.

#### 3.3.2. Radar and Other Threat Models

Radar and other threat regions are stored separately as

(11) $$M^{r}=\left\{M_{1}^{r},\ldots ,M_{N_{r}}^{r}\right\}$$

(12) $$M^{o}=\left\{M_{1}^{o},\ldots ,M_{N_{o}}^{o}\right\}$$

Each threat region is represented as

(13) $$M_{m}=\left(C_{m},R_{m}\right)$$

where $C_{m}=\left(c_{m,x},c_{m,y},c_{m,z}\right)$ is the center of threat region *m*, and $R_{m}$ is its radius.

In the mathematical formulation, radar and other threats are modeled as spheres, and Equations (14)–(19) define the reference three-dimensional segment–sphere intersection test that should be used by an independent validator. The visualization program uses the stored centers and radii to display each threat object. The reported optimization runs, however, use the legacy coded approximation described after Equation (19), not a separately verified implementation of these reference equations.

Consider a flight segment with endpoints

(14) $$P_{i,k},\;P_{i,k+1}$$

Let

(15) $$q_{i,k}=P_{i,k+1}-P_{i,k}$$

The projection factor from the threat center onto this segment is

(16) $$\eta_{i,k,m}=\frac{{\left(C_{m}-P_{i,k}\right)}^{T}q_{i,k}}{{∥q_{i,k}∥}_{2}^{2}}$$

The factor is clamped to the segment as follows:

(17) $${\bar{\eta }}_{i,k,m}=\min\left[1,\max\left(0,\eta_{i,k,m}\right)\right]$$

The minimum distance from the threat center to the flight segment is

(18) $$d_{i,k,m}={∥P_{i,k}+{\bar{\eta }}_{i,k,m}q_{i,k}-C_{m}∥}_{2}$$

If

(19) $$d_{i,k,m}<R_{m}$$

the segment is considered to intersect threat region m.

The independent reference validator should implement the clamped projection above. The legacy experimental evaluator first tests the endpoints and then applies a line-distance/cosine branch; that branch has not been shown to be algebraically equivalent to Equations (16)–(19). In addition, the current other-threat cost path inherits a radar-radius lookup rather than using the stored other-threat radius in every term. Accordingly, the reported threat counts and composite fitness describe the executed code and are not claimed as exact reference segment–sphere validation.

#### 3.3.3. No-Fly-Zone Model

No-fly zones are regions that UAVs are prohibited from entering during a mission. Each zone is modeled as a vertical cylinder with parameters

(20) $$N_{j}=\left(c_{j,x}^{n},c_{j,y}^{n},h_{j}^{n},R_{j}^{n}\right)$$

where $\left(c_{j,x}^{n},c_{j,y}^{n}\right)$ gives the horizontal position of the cylinder axis, $R_{j}^{n}$ is the no-fly-zone radius, and $h_{j}^{n}$ is its upper altitude.

A waypoint lies within a no-fly zone when

(21) $$\sqrt{{\left(x_{i,k}-c_{j,x}^{n}\right)}^{2}+{\left(y_{i,k}-c_{j,y}^{n}\right)}^{2}}\leq R_{j}^{n}$$

and its altitude also lies within the vertical bounds of the zone. Radar threats, other threats, and no-fly zones are visualized as distinct environmental objects. Radar and other threats are spherical, whereas no-fly zones are cylindrical. Subsequent statistics report threat-affected segments for the spherical regions. No-fly-zone violations are evaluated jointly from the corresponding objective term and the plotted trajectory.

### 3.4. Multi-UAV Path Encoding and Decoding

#### 3.4.1. Path Encoding

Directly optimizing the Cartesian coordinates of all intermediate waypoints does not explicitly represent motion between adjacent nodes. It can therefore produce disordered jumps, path reversals, and large turning angles. To reduce these effects, this study uses spherical-increment encoding. The path and speed variables of UAV *i* are represented as

(22) $$X_{i}=\left[\rho_{i},\psi_{i},\phi_{i},v_{i}\right]$$

where $\rho_{i}=\left[\rho_{i,1},\ldots ,\rho_{i,n_{i}}\right]$ contains radial increments, $\psi_{i}=\left[\psi_{i,1},\ldots ,\psi_{i,n_{i}}\right]$ contains elevation angles, $\phi_{i}=\left[\phi_{i,1},\ldots ,\phi_{i,n_{i}}\right]$ contains azimuth angles, and $v_{i}$ is the constant flight speed of UAV *i*. The complete joint decision vector is $X=[X_{1},X_{2},\ldots ,X_{N_{u}}]$. This spherical-increment encoding explicitly represents the length and direction of each segment, aligns the decision variables with UAV motion, and facilitates continuous candidate paths [21].

#### 3.4.2. Ranges of Path Variables

Let the straight-line distance between the start and target points of UAV *i* be

(23) $$D_{i}^{\mathrm{sg}}={∥G_{i}-S_{i}∥}_{2}$$

The nominal segment length is determined from the endpoint distance and the number of intermediate waypoints:

(24) $$r_{i,\max}=\frac{2D_{i}^{\mathrm{sg}}}{n_{i}}$$

The basic range of the radial-increment variable is

(25) $$0\leq \rho_{i,k}\leq r_{i,\max}$$

During coordinate decoding, the program adds a nominal step length to the radial variable:

(26) $$r_{i,k}=\rho_{i,k}+\frac{r_{i,\max}}{2}$$

Consequently, the actual segment length varies around the nominal endpoint spacing, reducing the likelihood of excessive overlap between adjacent waypoints.

The elevation-angle range is set to

(27) $$-\frac{\pi }{4}\leq \psi_{i,k}\leq \frac{\pi }{4}$$

The baseline azimuth from the start point toward the target point is first calculated as

(28) $$\phi_{i}^{0}=\mathrm{atan2}\left(x_{i}^{g}-x_{i}^{s},y_{i}^{g}-y_{i}^{s}\right)$$

Here, the superscripts *s* and *g* denote the start and goal coordinates, respectively, and the subscript *i* identifies the UAV. This notation replaces the ambiguous superscripts in the previous rendering of Equation (28).

The azimuth search range is then set to

(29) $$\phi_{i}^{0}-\frac{\pi }{4}\leq \phi_{i,k}\leq \phi_{i}^{0}+\frac{\pi }{4}$$

The radial, elevation-angle, and azimuth ranges are generated automatically from the start and target points of each UAV.

#### 3.4.3. Conversion from Spherical Increments to Cartesian Coordinates

Using the start point of UAV *i* as the reference position, the first intermediate waypoint is calculated as

(30) $$x_{i,1}=x_{i}^{s}+r_{i,1}\cos\psi_{i,1}\sin\phi_{i,1}$$

(31) $$y_{i,1}=y_{i}^{s}+r_{i,1}\cos\psi_{i,1}\cos\phi_{i,1}$$

(32) $$z_{i,1}=z_{i}^{s}+r_{i,1}\sin\psi_{i,1}$$

The remaining waypoints are generated recursively:

(33) $$x_{i,k}=x_{i,k-1}+r_{i,k}\cos\psi_{i,k}\sin\phi_{i,k}$$

(34) $$y_{i,k}=y_{i,k-1}+r_{i,k}\cos\psi_{i,k}\cos\phi_{i,k}$$

(35) $$z_{i,k}=z_{i,k-1}+r_{i,k}\sin\psi_{i,k}$$

where $k=2,3,\ldots ,n_{i}$. After each intermediate waypoint is calculated, its *x*, *y*, and *z* coordinates are clipped to the allowable bounds. The final $N_{u}$ variables in the joint decision vector represent UAV speeds and are repaired to their corresponding lower and upper bounds.

#### 3.4.4. Decision-Vector Dimension

UAV *i* has $n_{i}$ radial-increment variables, $n_{i}$ elevation-angle variables, and $n_{i}$ azimuth variables, together with one speed variable. Its decision dimension is

(36) $$D_{i}=3n_{i}+1$$

The total dimension of the joint multi-UAV path-planning problem is

(37) $$D=\sum_{i=1}^{N_{u}}\left(3n_{i}+1\right)$$

For $N_{u}=5$ and $n_{i}=15$, the decision dimension per UAV is 46.

The joint decision vector therefore has D=230 dimensions.

The joint multi-UAV path–speed planning problem is thus a 230-dimensional continuous constrained-optimization problem.

### 3.5. UAV Flight Constraints

#### 3.5.1. Spatial-Boundary Constraints

Every UAV waypoint must satisfy

(38) $$x_{\min}\leq x_{i,k}\leq x_{\max}$$

(39) $$y_{\min}\leq y_{i,k}\leq y_{\max}$$

(40) $$z_{\min}\leq z_{i,k}\leq z_{\max}$$

Boundary repair handles spatial-boundary violations. A coordinate above or below its allowable range is clipped to the corresponding upper or lower bound. This procedure keeps every decoded intermediate waypoint within the mission space.

#### 3.5.2. Speed Constraints

The speed of UAV *i* must satisfy

(41) $$v_{i,\min}\leq v_{i}\leq v_{i,\max}$$

Speed determines each UAV’s flight time and changes when it reaches potential conflict regions. Speed and path parameters are therefore optimized jointly to reduce inter-UAV conflicts and mission delays through temporal coordination. The program extracts each UAV’s speed from the end of the joint decision vector and repairs any bound violations before decoding the path.

#### 3.5.3. Turning and Pitch-Angle Constraints

Let the direction vector of segment k be

(42) $$e_{i,k}=P_{i,k+1}-P_{i,k}=\left(\Delta x_{i,k},\Delta y_{i,k},\Delta z_{i,k}\right)$$

The horizontal heading of this segment is

(43) $$\alpha_{i,k}=atan2\left(\Delta y_{i,k},\Delta x_{i,k}\right)$$

and its pitch angle is

(44) $$\beta_{i,k}=\arctan\left[\frac{\Delta z_{i,k}}{\sqrt{\Delta x_{i,k}^{2}+\Delta y_{i,k}^{2}}}\right]$$

The changes in heading and pitch between consecutive segments are, respectively,

(45) $$\Delta \alpha_{i,k}=\alpha_{i,k}-\alpha_{i,k-1}$$

(46) $$\Delta \beta_{i,k}=\beta_{i,k}-\beta_{i,k-1}$$

To satisfy UAV maneuverability limits,

(47) $$\left|\Delta \alpha_{i,k}\right|\leq \alpha_{i,\max}$$

(48) $$\left|\Delta \beta_{i,k}\right|\leq \beta_{i,\max}$$

The trajectory-checking program calculates the heading and pitch of adjacent segments using atan2 and determines whether either limit is violated. A waypoint is marked as an angular-constraint violation when a change exceeds the corresponding upper bound.

#### 3.5.4. Minimum-Segment-Length Constraint

Excessively short segments can create dense waypoint clusters, increase command-switching frequency, and introduce path changes with little practical value. The length of segment k is defined as

(49) $$l_{i,k}={∥P_{i,k+1}-P_{i,k}∥}_{2}$$

and must satisfy

(50) $$l_{i,k}\geq l_{i,\min}$$

The program checks every segment, including the start-to-first-waypoint segment, all intermediate segments, and the final-waypoint-to-target segment. Any segment shorter than the specified threshold is recorded as a minimum-segment-length violation.

#### 3.5.5. Threat Avoidance

Every segment of every UAV path should satisfy

(51) $$d_{i,k,m}\geq R_{m}+d_{\mathrm{margin}}$$

where $d_{\mathrm{margin}}$ is the prescribed safety-buffer distance.

When

(52) $$d_{i,k,m}<R_{m}$$

the segment intersects the threat region. For a binary test, the indicator is defined as

(53) $$I_{i,k,m}^{T}=\left\{\begin{array}{c} 1,d_{i,k,m}<R_{m},\\ 0,d_{i,k,m}\geq R_{m}. \end{array}\right.\;$$

The program records intersections with radar and other threats separately. These indicators are combined with angular and minimum-segment-length violations when the constraint status of each waypoint is assembled.

#### 3.5.6. Arrival-Time Constraints

The cumulative path length of UAV *i* from the start point to waypoint *k* is

(54) $$L_{i,k}=\sum_{q=0}^{k}l_{i,q}$$

If the UAV departs at time $t_{i}^{0}$ and flies at speed $v_{i}$, its arrival time at waypoint *k* is

(55) $$t_{i,k}=t_{i}^{0}+\frac{L_{i,k}}{v_{i}}$$

Its actual arrival time at the target point is

(56) $$T_{i}^{a}=t_{i}^{0}+\frac{L_{i}}{v_{i}}$$

where $L_{i}$ is the total path length.

Given the expected arrival time, the delay is

(57) $$\Delta T_{i}=T_{i}^{a}-T_{i}^{e}$$

Because early arrival incurs no delay penalty, the nonnegative delay is defined as

(58) $$\Delta T_{i}^{+}=\max\left(\Delta T_{i},0\right)$$

The detection program calculates waypoint and final arrival times as the departure time plus cumulative path length divided by speed. The statistical program further reports total delay and maximum delay.

#### 3.5.7. Inter-UAV Collision Constraints

Spatial paths may intersect without causing a collision when UAVs traverse the intersection at different times. Collision assessment must therefore consider position and time jointly.

Let the positions of UAVs i and j at time t be

(59) $$p_{i}\left(t\right),\;p_{j}\left(t\right)$$

Their separation distance is

(60) $$d_{\mathrm{ij}}\left(t\right)={∥p_{i}\left(t\right)-p_{j}\left(t\right)∥}_{2}$$

Any pair of UAVs must satisfy

(61) $$d_{\mathrm{ij}}\left(t\right)\geq d_{\mathrm{safe}},\;i\neq j$$

where $d_{\mathrm{safe}}$ is the minimum safe separation between UAVs.

For a segment of UAV j,

(62) $$\left[P_{j,q-1},P_{j,q}\right]$$

its traversal-time interval is

(63) $$\left[t_{j,q-1},t_{j,q}\right]$$

If evaluation time t lies within this interval, the UAV position is estimated by linear interpolation:

(64) $$p_{j}\left(t\right)=P_{j,q-1}+\frac{t-t_{j,q-1}}{t_{j,q}-t_{j,q-1}}\left(P_{j,q}-P_{j,q-1}\right)$$

This position is then compared with that of UAV i at the same time.

The program evaluates every unordered UAV pair. At each breakpoint time of one UAV, the position of the other UAV on its corresponding segment is estimated by linear interpolation. A sampled proximity violation is recorded when their simultaneous separation is below the prescribed safety distance; otherwise, no violation is recorded at the sampled times. This test does not establish continuous-time collision freedom between breakpoints.

### 3.6. Objective Function for Multi-UAV Path Planning

#### 3.6.1. Flight Cost

Path length is an important indicator of flight-energy consumption and mission efficiency. The total path length of UAV *i* is

(65) $$L_{i}=\sum_{k=0}^{n_{i}}{∥P_{i,k+1}-P_{i,k}∥}_{2}$$

The total path length of all UAVs is

(66) $$L_{\mathrm{total}}=\sum_{i=1}^{N_{u}}L_{i}$$

To reduce the effect of different map scales on the objective values, this sum is normalized by the total straight-line distance between each UAV’s endpoints:

(67) $$J_{L}=\frac{L_{\mathrm{total}}}{\sum_{i=1}^{N_{u}}{∥G_{i}-S_{i}∥}_{2}}$$

A smaller $J_{L}$ indicates a shorter total path across all UAVs.

After optimization, the main program decodes the UAV paths and calculates the individual path lengths, total length, mean length, maximum length, and standard deviation of path length.

#### 3.6.2. Threat Cost

Let $N_{T}$ denote the total number of radar and other threat regions. The threat cost is defined as

(68) $$J_{T}=\sum_{i=1}^{N_{u}}\sum_{k=0}^{n_{i}}\sum_{m=1}^{N_{T}}I_{i,k,m}^{T}$$

A continuous threat cost can also be defined to represent the depth to which a UAV enters a threat region:

(69) $$J_{T}^{d}=\sum_{i=1}^{N_{u}}\sum_{k=0}^{n_{i}}\sum_{m=1}^{N_{T}}\max\left(0,\frac{R_{m}-d_{i,k,m}}{R_{m}}\right)$$

Equation (69) distinguishes a shallow boundary crossing from deeper penetration into a threat region. The reported experimental statistics use the number of threat-affected segments defined in Equation (68).

#### 3.6.3. Turning-Angle Cost

Horizontal-heading and pitch-angle violations are defined as

(70) $$J_{\alpha }=\sum_{i=1}^{N_{u}}\sum_{k=1}^{n_{i}}\max\left[0,\frac{\left|\Delta \alpha_{i,k}\right|-\alpha_{i,\max}}{\alpha_{i,\max}}\right]$$

(71) $$J_{\beta }=\sum_{i=1}^{N_{u}}\sum_{k=1}^{n_{i}}\max\left[0,\frac{\left|\Delta \beta_{i,k}\right|-\beta_{i,\max}}{\beta_{i,\max}}\right]$$

The combined angular cost is

(72) $$J_{A}=J_{\alpha }+J_{\beta }$$

When all heading and pitch changes between consecutive segments remain within their limits,

(73) $$J_{A}=0$$

#### 3.6.4. Minimum-Segment-Length Cost

A minimum-segment-length violation is defined as

(74) $$J_{S}=\sum_{i=1}^{N_{u}}\sum_{k=0}^{n_{i}}\max\left[0,\frac{l_{i,\min}-l_{i,k}}{l_{i,\min}}\right]$$

When all segments satisfy the minimum-length constraint,

(75) $$l_{i,k}\geq l_{i,\min}$$

then $J_{S}=0$.

This term discourages paths that are mathematically continuous but physically impractical because they contain many nearly overlapping waypoints.

#### 3.6.5. Delay Cost

The total multi-UAV delay cost is

(76) $$J_{D}=\sum_{i=1}^{N_{u}}\frac{\Delta T_{i}^{+}}{T_{i}^{e}}$$

If all UAVs arrive at or before their expected arrival times,

(77) $$J_{D}=0$$

In addition to the total delay, the maximum delay is defined as

(78) $$J_{D}^{\max}=\max_{1\leq i\leq N_{u}}\Delta T_{i}^{+}$$

This term prevents severe delay in a small number of UAVs from being obscured by the total.

#### 3.6.6. Sampled Inter-UAV Separation Cost

Define the sampled inter-UAV separation-violation indicator as

(79) $$I_{\mathrm{ij}}^{C}\left(t\right)=\left\{\begin{array}{c} 1,d_{\mathrm{ij}}\left(t\right)<d_{\mathrm{safe}},\\ 0,d_{\mathrm{ij}}\left(t\right)\geq d_{\mathrm{safe}}. \end{array}\right.\;$$

Under discrete-time evaluation, the sampled separation cost is

(80) $$J_{C}=\sum_{i=1}^{N_{u}-1}\sum_{j=i+1}^{N_{u}}\sum_{t\in T_{ij}}I_{ij}^{C}\left(t\right)$$

where $T_{ij}$ is the set of sampled times shared by UAVs *i* and *j*. Sampled separation violations directly affect mission safety and are therefore assigned a larger penalty weight than ordinary path length. When

(81) $$J_{C}>0$$

the solution is not strictly safe or feasible.

#### 3.6.7. Terrain and No-Fly-Zone Costs

Define the terrain-safety violation function as

(82) $$I_{i,k}^{H}=\left\{\begin{array}{c} 1,z_{i,k}<H\left(x_{i,k},y_{i,k}\right)+h_{\mathrm{safe}},\\ 0,z_{i,k}\geq H\left(x_{i,k},y_{i,k}\right)+h_{\mathrm{safe}}. \end{array}\right.\;$$

The terrain cost is defined as

(83) $$J_{H}=\sum_{i=1}^{N_{u}}\sum_{k=1}^{n_{i}}I_{i,k}^{H}$$

For a no-fly-zone indicator, $\chi_{i,k,j}^{N}$, the corresponding cost records whether a waypoint or adjacent segment of UAV i enters no-fly zone j.

(84) $$J_{N}=\sum_{i=1}^{N_{u}}\sum_{k=0}^{n_{i}}\sum_{j=1}^{N_{N}}\chi_{i,k,j}^{N}$$

Terrain clearance and no-fly-zone avoidance are safety requirements in the formulated model. In the reported implementation, the term occupying the $J_{H}$ position is an absolute altitude-band surrogate, not the DEM-clearance indicator in Equation (82), while no-fly-zone entry is represented by a segment-based penalty. The exported Feasible field independently re-evaluates neither DEM clearance nor no-fly-zone entry. These conditions must therefore be checked by the comprehensive post-optimization validator; a low objective value or Feasible label is not, by itself, proof of a safe path.

#### 3.6.8. Composite Objective Function

A conceptual eight-requirement objective for cooperative three-dimensional multi-UAV path planning can be written as

(85) $$F_{\mathrm{ref}}\left(X\right)=\omega_{1}J_{L}+\omega_{2}J_{H}+\omega_{3}J_{N}+\omega_{4}J_{T}+\omega_{5}J_{A}+\omega_{6}J_{S}+\omega_{7}J_{D}+\omega_{8}J_{C}$$

where

(86) $$\omega_{q}>0,\;q=1,2,\ldots ,8$$

where the coefficients are positive requirement-level weights. The implementation represents these eight requirement categories with nine code-level components because timing includes both arrival-window deviation and tardiness. The components are normalized path length $f_{o}$, altitude-band surrogate $f_{h}$, threat cost $f_{t}$, arrival-window deviation $f_{m}$, sampled inter-UAV proximity $f_{c}$, angle and minimum-segment indicators $f_{a}$ and $f_{tr}$, plan-view no-fly-zone penalty $f_{no}$, and quadratic tardiness $f_{delay}$. The repeated experiments evaluate the following objective:

$$F_{\mathrm{impl}}=0.05f_{o}+0.05f_{h}+10000f_{t}+0.7f_{m}+0.7f_{c}+0.6f_{a}+0.9f_{tr}+20000f_{no}+f_{delay}.$$

Here, $f_{m}$ is zero only inside the implemented arrival window and otherwise grows with absolute deviation, whereas $f_{delay}$ is applied only to late arrival and grows quadratically. Table 1, Table 2 and Table 3, Figure 1, Figure 2 and Figure 3, and all reported repeated-run statistics refer to $F_{\mathrm{impl}}$.

To emphasize safety, the weights assigned to safety-related costs are larger than the path-length weight:

(87) $$\omega_{8},\omega_{3},\omega_{2},\omega_{4}>\omega_{1}$$

The coefficients in $F_{\mathrm{impl}}$ serve two implementation purposes: balancing component scales and encoding planning priorities. The continuously varying path-length and altitude-surrogate terms receive weights of 0.05 so that routine changes in distance or altitude do not dominate the remaining penalties. Threat and no-fly-zone terms are assigned weights of 10,000 and 20,000 because they represent safety-critical region incursions and should dominate ordinary path shortening. The intermediate coefficients (0.6–0.9) retain the influence of timing, sampled proximity, angular, and minimum-segment terms, while the quadratic tardiness term retains unit weight. These coefficients are engineering penalty factors used consistently for every algorithm and repeated run within a scenario; they were not optimized separately for any algorithm. Because the components are not uniformly normalized, the coefficients should not be interpreted as physical trade-off rates. Moreover, large penalties discourage violations but cannot impose hard feasibility, because every finite penalty remains exchangeable with other objective components. This limitation motivates the explicit feasibility-priority ordering in Equation (94).

The implemented cost components use different scales and are not uniformly normalized. Nevertheless, every comparison algorithm uses the same objective, weights, and constraint parameters within each scenario, ensuring a consistent algorithmic comparison.

### 3.7. Feasibility Determination

For reproducible post hoc reporting, the current implementation records a limited feasibility screen based on the following four counted conditions:

(88) $$N_{T}=0$$

(89) $$N_{A}=0$$

(90) $$N_{S}=0$$

(91) $$N_{C}=0$$

Here, $N_{T}$ is the total number of threat-affected segments, $N_{A}$ is the number of angular-constraint violations, $N_{S}$ is the number of minimum-segment-length violations, and $N_{C}$ is the number of sampled inter-UAV separation violations.

The feasibility rule used in the program is expressed as

(92) $$Feasible=\left\{\begin{array}{c} 1,N_{T}=0\wedge N_{A}=0\wedge N_{S}=0\wedge N_{C}=0,\\ 0,otherwise. \end{array}\right.\;$$

Equations (88)–(92) describe the legacy four-count screen used with the original penalty-only experiment. It is distinct from the penalty-weighted ordering used during that search and does not constitute a complete flight-safety certificate. The legacy repeated experiment produced 0/120 passing IWOA solutions and 0/960 passing solutions overall, which motivated the feasibility-priority experiment described below rather than being presented as an acceptable feasibility result.

For the added feasibility-priority study, a corrected validator computes five non-negative normalized violation magnitudes: segment penetration into modeled threat spheres, wrapped heading/pitch excess, normalized minimum-segment shortfall, continuous-time inter-UAV separation shortfall for every pair of overlapping piecewise-linear segments, and plan-view no-fly-zone penetration. The heading change is wrapped with atan2(sinΔψ,cosΔψ), and the azimuth search corridor is centered using the convention implemented by the spherical decoder. The aggregate violation is

(93) $$CV\left(X\right)=CV_{T}+CV_{A}+CV_{S}+CV_{C}+CV_{N},\;Feasible_{FP}\left(X\right)=I\left[CV\left(X\right)\leq 10^{-10}\right].$$

Candidates are then ordered using a scalar implementation of Deb’s feasibility-priority rule. Any feasible candidate precedes every infeasible candidate; feasible candidates are ranked through a monotone transform of the implemented composite objective $F_{\mathrm{impl}}$; and infeasible candidates are ranked by CV. The scalar value supplied to the unchanged minimization interfaces is

(94) $$F_{FP}\left(X\right)=\left\{\begin{array}{cc} F_{+}\left(X\right)/\left(1+F_{+}\left(X\right)\right), & CV\left(X\right)\leq 10^{-10},\\ 1+CV(X), & CV\left(X\right)>10^{-10}. \end{array}\right.$$

Here, $F_{+}\left(X\right)=\max\left\{0,F_{\mathrm{impl}}\left(X\right)\right\}$, matching the executed code. Equation (94) preserves the intended ordering for the non-negative implemented objectives in exact arithmetic. In the stored finite-precision implementation, extremely close feasible objective values can map to the same scalar score; such numerical ties do not affect the feasible-before-infeasible ordering and are retained rather than hidden.

Before the formal repeated runs, preliminary feasibility-priority runs generated one feasible pilot solution for each scenario. Each solution was rechecked with Equation (93), archived with its provenance, and excluded from the formal statistics. Exactly the same scenario-specific incumbent was then inserted as one initial population member for IWOA-FP and WOA-FP; all other population members and stochastic updates used the paired run seeds. Elitist best-so-far retention and Equation (94) therefore test whether the optimizers preserve and improve a known feasible plan. The resulting proportion is a feasibility-preservation rate, not the probability of discovering feasibility from an entirely random population. The validator omits complete DEM clearance, timing acceptance, smoothed-curve validation, vehicle dynamics, and uncertainty, so $Feasible_{FP}$ remains a modeled constraint outcome rather than flight-safety certification.

### 3.8. Model Solution Procedure

The cooperative three-dimensional multi-UAV paths are obtained using the encoding and implemented objective described above. The terrain matrix, radar threats, other threats, and no-fly zones are loaded for the selected map and complexity setting, together with each UAV’s start point, target point, departure time, expected arrival time, and flight-performance limits. Radial-increment ranges are determined from endpoint distances and the number of intermediate waypoints; azimuth, elevation-angle, and speed bounds are then specified. The final $N_{u}$ variables are decoded as UAV speeds, and the remaining variables are grouped by UAV into radial increments, elevation angles, and azimuth angles. Equations (30)–(35) yield the three-dimensional waypoints. The current program evaluates boundaries, coded threat intersections, turning angles, minimum segment lengths, arrival-time delay, segment-based no-fly-zone penalties, an absolute altitude-band surrogate, and discretely sampled inter-UAV proximity. The resulting composite fitness is an implementation-specific optimization score rather than a complete constraint certificate.

The optimizer updates the population until the maximum number of iterations is reached and returns the best joint decision vector, best fitness, and convergence curve. To verify consistency between the reported optimum and the objective function, the main program reevaluates the returned solution to obtain CheckedFitness. It then decodes the solution and reports total path length, mission-completion time, delay, threat-affected segments, angular violations, minimum-segment-length violations, and sampled inter-UAV separation violations. The complete procedure can therefore be summarized as environment initialization, joint path–speed encoding, population-based optimization, path decoding, constraint evaluation, fitness verification, and output of the best plan.

In the added feasibility-priority branch, the implemented composite objective is preserved for conditional comparison, whereas selection is governed by Equation (94). The final solution is recomputed after each run using the same corrected validator, and the result file stores CV, its five components, violation counts, $F_{\mathrm{impl}}$, $F_{FP}$, and the score-recomputation error. This is an internal consistency check rather than an independent safety validation; the separation makes it possible to report constraint satisfaction before comparing composite objective values.

B-spline smoothing is used only to improve visual continuity and is not used to compute the tabulated fitness or feasibility indicators. A smoothed curve must not be released as an executable trajectory until it passes a dense post-smoothing validation gate. The recommended gate samples each smoothed segment at a spacing no greater than one tenth of the prescribed safety distance, recomputes terrain clearance, threat/no-fly-zone intersection, turning and pitch angles, minimum segment length, arrival time, and time-indexed pairwise separation, and then either accepts the curve or rejects it and restores the validated piecewise-linear path. The present study does not claim that the displayed smoothed curves passed this gate.

## 4. Multi-Strategy Improved Whale Optimization Algorithm

### 4.1. Original WOA

The WOA performs continuous-space optimization by simulating humpback-whale prey encirclement, spiral bubble-net attacks, and random search [31].

Let N be the population size and D the problem dimension. The position of whale i at iteration t is represented as

(95) $$X_{i}\left(t\right)=\left[x_{i,1}\left(t\right),x_{i,2}\left(t\right),\ldots ,x_{i,D}\left(t\right)\right]$$

The position of the individual with the best current fitness is

(96) $$X^{*}\left(t\right)$$

#### 4.1.1. Prey-Encirclement Phase

WOA approximates the prey position using the best current individual, and the remaining whales update their positions accordingly. The distance vector between a whale and the estimated prey is

(97) $$D_{i}=\left|C⊙X^{*}\left(t\right)-X_{i}\left(t\right)\right|$$

The whale position is updated as

(98) $$X_{i}\left(t+1\right)=X^{*}\left(t\right)-A⊙D_{i}$$

where the dot denotes the Hadamard product. The coefficient vectors A and C are defined as

(99) $$A=2ar_{1}-a$$

(100) $$C=2r_{2}$$

where r_1_ and r_2_ are independent random vectors uniformly distributed over [0, 1], and a decreases linearly with iteration number:

(101) $$a=2-\frac{2t}{T}$$

where T is the maximum number of iterations.

When

(102) $$\left|A\right|<1$$

the whale updates its position around the best current individual, emphasizing local exploitation. As a decreases linearly from 2 to 0, the range of A contracts and the population increasingly concentrates around the current best solution.

#### 4.1.2. Spiral Bubble-Net Attack Phase

WOA uses a logarithmic spiral to simulate a humpback whale approaching its prey. The distance between whale i and the current best individual is

(103) $$D_{i}^{\prime }=\left|X^{*}\left(t\right)-X_{i}\left(t\right)\right|$$

The spiral position-update equation is

(104) $$X_{i}\left(t+1\right)=D_{i}^{\prime }e^{\mathrm{bl}}\cos\left(2\pi l\right)+X^{*}\left(t\right)$$

where b is a constant controlling the spiral shape, set to b = 1, and l is a random number in [−1, 1].

Encirclement or random search and spiral attack are selected with equal probability. Let p be uniformly distributed over [0, 1]. Encirclement or random search is performed when *p* < 0.5, whereas spiral attack is performed when *p* ≥ 0.5.

#### 4.1.3. Random-Search Phase


(105) $$\left|A\right|\geq 1$$

Rather than following the best current individual, each whale randomly selects an individual $X_{\mathrm{rand}}\left(t\right)$ from the population and updates its position relative to that individual.

The distance between a whale and the randomly selected individual is

(106) $$D_{\mathrm{rand}}=\left|C⊙X_{\mathrm{rand}}\left(t\right)-X_{i}\left(t\right)\right|$$

The position-update equation is

(107) $$X_{i}\left(t+1\right)=X_{\mathrm{rand}}\left(t\right)-A⊙D_{\mathrm{rand}}$$

Random search allows whales to move away from the current best individual and explore other regions, thereby expanding the search range during early iterations.

Combining these three behaviors, the original WOA position-update model is

(108) $$X_{i}\left(t+1\right)=\left\{\begin{array}{c} X^{*}\left(t\right)-A⊙D_{i},p<0.5,\;\left|A\right|<1,\\ X_{\mathrm{rand}}\left(t\right)-A⊙D_{\mathrm{rand}},p<0.5,\;\left|A\right|\geq 1,\\ D_{i}^{\prime }e^{\mathrm{bl}}\cos\left(2\pi l\right)+X^{*}\left(t\right),p\geq 0.5. \end{array}\right.\;$$

The original WOA has a simple structure, requires few parameters, and uses no objective-function gradients, making it suitable for continuous optimization. However, it presents four limitations for the high-dimensional joint path–speed problem considered here. First, random initialization cannot ensure uniform coverage of a high-dimensional search space with a limited population. Second, a fixed linear convergence factor cannot adapt flexibly across search stages. Third, progressive concentration around the current best individual reduces diversity and increases the risk of stagnation. Fourth, the original algorithm lacks a heavy-tailed mutation mechanism for perturbing the best solution after convergence to a local optimum. IWOA addresses these limitations through good-point-based initialization, adaptive search weighting, population migration, and Cauchy mutation.

### 4.2. Improved WOA

The evaluated production IWOA retains WOA’s prey-encirclement, random-search, and spiral bubble-net-attack mechanisms. It highlights four modifications: deterministic good-point-based initialization, nonlinear adaptive weighting during encirclement, population migration, and a Cauchy-based candidate generated from the incumbent best. The executable code also retains pooling, preferential-selection, Dynamic Fitness-Distance Balance (DFDB), and Sobol Opposition-Based Learning (SOBL) routines. The following subsections explain the four highlighted mechanisms. However, the main numerical results represent the complete legacy production code and cannot isolate the effect of these four mechanisms alone.

The coefficient equations in Section 4.1 describe standard WOA as background. The production routine does not use the linearly decreasing vector $A=2ar_{1}-a$ in Equation (98). At each iteration it instead samples one scalar coefficient per candidate as $A_{i}=0.5+0.1\tan\left[\pi \left(u_{i}-0.5\right)\right]$, resamples nonpositive values, clips values above 1, and then adds one shared standard-normal draw before using the $A_{i}<0.5$ versus $A_{i}\geq 0.5$ branches. This implementation-specific rule is another reason Algorithm 1 is treated as a conceptual overview rather than executable pseudocode.

#### 4.2.1. Good-Point-Set Population Initialization

The original WOA is generally initialized using a uniform random distribution:

(109) $$x_{i,j}=lb_{j}+r_{i,j}\left(ub_{j}-lb_{j}\right)$$

where $r_{i,j}$ is uniformly distributed over [0,1], and $lb_{j}$ and $ub_{j}$ are the lower and upper bounds of decision variable j, respectively.

Although random initialization is simple, a limited population may cluster locally in a high-dimensional problem. Equations (109)–(112) state a general multidimensional good-point construction [30]. Let the population size be *N* and the problem dimension be *D*. Select the smallest prime *p* satisfying

(110) p≥2D+3

and construct the generating vector for the good-point set as

(111) $$h_{j}=2\cos\left(\frac{2\pi j}{p}\right),\;j=1,2,\ldots ,D$$

The jth standardized coordinate of point i is

(112) $$q_{i,j}=\mathrm{mod}\left(i\;h_{j},\;1\right),\;i=1,2,\ldots ,N$$

Here, mod(·,·) returns the fractional part, so $q_{i,j}$ lies in [0, 1]. The good-point coordinates are mapped to the actual search space as

(113) $$x_{i,j}\left(0\right)=lb_{j}+q_{i,j}\left(ub_{j}-lb_{j}\right)$$

The evaluated production routine does not execute this general *D*-dimensional construction for D>1. Scalar bounds are expanded to *D*-vectors before initialization, after which the routine calls the one-dimensional good-point generator separately for every coordinate. Because each call is deterministic, the same normalized sequence is replicated across coordinates before coordinate-wise bound mapping in both the reported CEC2017 and UAV runs. Thus, normalized initial points lie on a rank-one sequence rather than a general *D*-dimensional good-point set. The results therefore support only a claim of deterministic stratified initialization, not verified high-dimensional coverage. The modular factor screen uses a separate full-dimensional construction, which is one reason it is not treated as a causal decomposition of the production IWOA implementation.

**Algorithm 1** Pseudocode of the IWOA
    Input: population size N, maximum iterations Tmax, problem dimension D, search bounds [lb,ub], and objective function F(X)    Output: best solution Xbest, best fitness Scorebest, and convergence curve Convergence

1  Initialize the whale population X using the good-point-based sequence;2  Evaluate F(X) and determine Xbest and Scorebest;3  Set t ← 1;4  while t ≤ Tmax do5  Calculate a, A, C, p, l, and w(t);6  for each whale individual Xi do7  if *p* < 0.5 then8  if |A| < 1 then9  Xi,new ← Xbest − w(t)A ⊙ |CXbest − Xi|;10else11Randomly select a whale Xrand;12Xi,new ← Xrand − A ⊙ |CXrand − Xi|;13end if14else15Xi,new ← |Xbest − Xi| · exp(bl) · cos(2πl) + Xbest;16end if17end for18Randomly select Nm whales and generate migration candidates;19Apply boundary repair and evaluate the updated candidates;20Update X, Xbest, and Scorebest using the greedy criterion;21Generate the Cauchy mutation candidate Xc;22if F(Xc) < Scorebest then23Xbest ← Xc; Scorebest ← F(Xc);24end if25Convergence(t) ← Scorebest; t ← t + 1;26end while


#### 4.2.2. Adaptive Weighting Strategy

The original WOA relies mainly on the linearly decreasing convergence factor a to regulate exploration and exploitation. A single linear schedule may not suit all search stages, because late iterations require smaller updates for local refinement. A nonlinear adaptive weight w(t) is therefore introduced into the prey-encirclement phase:

(114) $$w\left(t\right)=\alpha exp\left[-\beta exp\left(\gamma t\right)\right]+\delta$$

where the parameters are set as

(115) α=0.6,β=0.02,γ=0.04,δ=0.3

The derivative of the weight function with respect to iteration t is

(116) $$\frac{\mathrm{dw}}{\mathrm{dt}}=-\alpha \beta \gamma e^{\gamma t}\exp\left[-\beta e^{\gamma t}\right]$$

Because alpha, beta, and gamma are positive,

(117) $$\frac{\mathrm{dw}}{\mathrm{dt}}<0$$

which indicates that w(t) decreases monotonically with iteration number. The encirclement update is therefore rewritten as

(118) $$X_{i}\left(t+1\right)=X^{*}\left(t\right)-w\left(t\right)A⊙D_{i}$$

where the distance vector is still calculated as

(119) $$D_{i}=\left|C⊙X^{*}\left(t\right)-X_{i}\left(t\right)\right|$$

The adaptive weight works with the original convergence factor to regulate whale movement. A larger early-stage weight strengthens guidance by the best current individual, whereas the gradually reduced weight permits smoother local adjustments near promising regions and mitigates late-stage oscillation.

#### 4.2.3. Population-Migration Search Strategy

As iterations proceed, most whales gather near the best individual and their differences diminish. If the current best solution is locally optimal, conventional encirclement and spiral search may fail to leave the concentrated region. To restore population diversity, $N_{m}$ individuals are selected randomly at each iteration. A migration candidate is generated for each selected whale from a global random position and a weighted range derived from the current best individual. For the selected whale, *i*, the random coordinate in dimension *j* is $x_{i,j}^{\mathrm{rand}}=lb_{j}+r_{i,j}\left(ub_{j}-lb_{j}\right)$, where $r_{i,j}\in \left[0,1\right]$. The dimension-wise lower and upper bounds around the best individual are

(120) $$x_{\max}^{*}=\max_{1\leq j\leq D}x_{j}^{*}$$

(121) $$x_{\min}^{*}=\min_{1\leq j\leq D}x_{j}^{*}$$

A random reference value is generated within the interval [$x_{\min}^{*}$, $x_{\max}^{*}$]:

(122) $$x_{i,j}^{\mathrm{brnd}}=x_{\min}^{*}+q_{i,j}\left(x_{\max}^{*}-x_{\min}^{*}\right)$$

where $q_{i,j}$ is in [0, 1]. The migrated coordinate in dimension j is

(123) $$x_{i,j}^{\mathrm{mig}}=x_{i,j}^{\mathrm{rand}}-x_{i,j}^{\mathrm{brnd}}$$

In vector form, this becomes

(124) $$X_{i}^{\mathrm{mig}}=X_{i}^{\mathrm{rand}}-X_{i}^{\mathrm{brnd}}$$

After migration, boundary repair is applied to the candidate position:

(125) $$x_{i,j}^{\mathrm{mig}}=\left\{\begin{array}{c} lb_{j},x_{i,j}^{\mathrm{mig}}<lb_{j},\\ x_{i,j}^{\mathrm{mig}},lb_{j}\leq x_{i,j}^{\mathrm{mig}}\leq ub_{j},\\ ub_{j},x_{i,j}^{\mathrm{mig}}>ub_{j}. \end{array}\right.\;$$

The fitness of each migrated individual is then evaluated, and the better position is retained using a greedy criterion.

This strategy expands candidate coverage through globally sampled positions while incorporating the current search state through a range centered on the best individual. It therefore regenerates diverse candidates when the population becomes concentrated. Here, population migration denotes position migration in swarm-intelligence optimization and is unrelated to neural-network parameter transfer.

#### 4.2.4. Cauchy Mutation Strategy

During late iterations, the population usually searches locally around the best individual. If that solution is locally optimal, conventional updates may not produce sufficiently large moves. The heavy-tailed Cauchy distribution can generate both small local perturbations and occasional large jumps, making it suitable for improving escape from local optima. Its standard probability density function is

(126) $$f\left(c\right)=\frac{1}{\pi \left(1+c^{2}\right)},\;-\infty <c<+\infty$$

Let a random variable be uniformly distributed over (0, 1). A standard Cauchy random variable can then be generated by inverse transformation:

(127) $$c=\tan\left[\pi \left(r-\frac{1}{2}\right)\right]$$

For a D-dimensional problem, generate the Cauchy random vector

(128) $$C_{\mathrm{cauchy}}=\left[c_{1},c_{2},\ldots ,c_{D}\right]$$

After the standard position update, a Cauchy-mutation candidate is generated from the current best individual:

(129) $$X_{c}=2X^{*}\left(t\right)+\eta X^{*}\left(t\right)⊙C_{\mathrm{cauchy}}$$

where η is the mutation scale factor, set to 0.1. The factor $2X^{*}\left(t\right)$ is shown explicitly because the production code forms $A_{\mathrm{mut}}=1+\eta C_{\mathrm{cauchy}}$ and then computes $X_{c}=X^{*}+X^{*}⊙A_{\mathrm{mut}}$. This offset means that even a small Cauchy draw is not a local perturbation around $X^{*}$; boundary repair is therefore an important part of the implemented candidate generation. The current best solution is updated using the greedy criterion:

(130) $$X^{*}\left(t\right)=\left\{\begin{array}{c} X_{c},F\left(X_{c}\right)<F\left(X^{*}\left(t\right)\right),\\ X^{*}\left(t\right),F\left(X_{c}\right)\geq F\left(X^{*}\left(t\right)\right). \end{array}\right.\;$$

The heavy-tailed candidate replaces the current best solution only when it has better fitness, so this extra trial cannot degrade the recorded optimum. In summary, deterministic good-point-based initialization changes the initial sample, adaptive weighting regulates encirclement updates, population migration supplies replacement candidates, and the Cauchy-based rule supplies one additional heavy-tailed trial per iteration. These are four highlighted mechanisms within the evaluated production IWOA; the additional legacy mechanisms are listed above.

### 4.3. IWOA Flowchart and Pseudocode

Figure 4 and Algorithm 1 provide a conceptual overview of the four highlighted mechanisms, and Table 4 summarizes their intended effects. They are not an exhaustive line-by-line representation of the executed production routine, which also contains pooling, preferential selection, DFDB, and SOBL. The released MATLAB code is the authoritative specification for reproducing the main numerical comparison.

### 4.4. Time-Complexity Analysis

The time complexity of IWOA depends on the population size *N*, problem dimension *D*, maximum number of iterations $T_{\max}$, and dimension-dependent cost C(D) of one objective-function evaluation. The number of whales selected for population migration is $N_{m}$, where $N_{m}\leq N$.

(131) $$\begin{array}{cc} O(IWOA)= & O(parameters)+O(initialization)+O(position\;update)+\\ & O(function\;evaluation)+O(adaptive\;weight)+\\ & O(migration\;search)+O(Cauchy\;mutation) \end{array}$$

The computational costs of the main steps are as follows:

1. Algorithm-parameter initialization has time complexity O(1).
2. Generating N D-dimensional individuals with a good-point set and mapping them to the search space has time complexity

(132) $$O\left(ND\right)$$

3. Evaluating the fitness of the N individuals in the initial population has time complexity

(133) $$O\left(NC(D)\right)$$

4. At each iteration, encirclement, random search, or spiral search updates all D dimensions of each of the N individuals at a cost of

(134) $$O\left(T_{\max}ND\right)$$

5. Evaluating the N updated individuals at every iteration incurs a total fitness-evaluation cost of

(135) $$O\left(T_{\max}NC\left(D\right)\right)$$

6. The adaptive weight is calculated once per iteration, with a total cost of

(136) $$O\left(T_{\max}\right)$$

7. During random migration, new *D*-dimensional positions are generated for $N_{m}$ whales before the ordinary population-evaluation loop. Migration therefore adds vector operations but no separate objective-evaluation pass, with total cost

(137) $$O\left(T_{\max}N_{m}D\right)$$

Because $N_{m}\leq N$, the worst-case migration cost does not exceed

(138) $$O\left(T_{\max}ND\right)$$

8. One D-dimensional Cauchy-mutation candidate is generated and evaluated at each iteration, giving a total cost of

(139) $$O\left(T_{\max}\left[D+C\left(D\right)\right]\right)$$

9. The cached opposition-based learning step constructs and evaluates *N* opposite candidates at each iteration, giving

(140) $$O\left(T_{\max}N\left[D+C\left(D\right)\right]\right).$$

Combining initialization, the standard population update and evaluation, migration generation, the single mutation candidate, and the opposition-based learning pass gives

(141) $$\begin{array}{cc} O(IWOA)= & O(N[D+C(D)]\\ \; & +T_{\max}\left\{2N\left[D+C\left(D\right)\right]+N_{m}D+D+C\left(D\right)\right\}). \end{array}$$

Since $N_{m}\leq N$, and omitting lower-order initialization and single-candidate terms for nontrivial $T_{\max}$, the worst-case order is

(142) $$O\left(IWOA\right)=O\left(T_{\max}N\left[D+C\left(D\right)\right]\right).$$

If evaluating one candidate is linear in the encoded dimension, C(D)=Θ(D), then

(143) $$O\left(IWOA\right)=O\left(T_{\max}ND\right).$$

If objective evaluation dominates vector operations, C(D)≫D, the expression may instead be written as

(144) $$O\left(IWOA\right)=O\left(T_{\max}NC\left(D\right)\right).$$

For the same objective and a migration fraction bounded independently of *N*, IWOA and standard WOA therefore have the same asymptotic order. IWOA nevertheless has larger constant factors because each iteration evaluates *N* opposition-based candidates and one Cauchy-mutation candidate, in addition to the *N* ordinary updated candidates. This result does not assume that objective-evaluation cost is independent of dimension.

## 5. Benchmark-Function Experiments and Performance Analysis of IWOA

We evaluated the numerical optimization performance of IWOA on the CEC2017 benchmark functions. The comparison included the original WOA, dung beetle optimization (DBO), particle swarm optimization (PSO), grey wolf optimization (GWO), the sparrow search algorithm (SSA), Harris hawks optimization (HHO), and ant colony optimization (ACO). Their different population structures and position-update mechanisms provide a broad comparison of global exploration, local exploitation, and escape from local optima.

Across multiple independent runs, the evaluation compared the minimum, mean, standard deviation, median, and maximum fitness values. Mean ranks, box plots, convergence curves, and Wilcoxon rank-sum tests characterized accuracy, result distributions, convergence behavior, and statistical differences. Representative functions were further examined through population search histories, one-dimensional trajectories, mean population fitness, exploration–exploitation ratios, and population diversity.

These experiments establish the general continuous-optimization performance of IWOA before its application to high-dimensional, multi-constraint three-dimensional UAV path planning.

### 5.1. Parameter Settings and Experimental Environment

All experiments were conducted on a Lenovo Legion R9000P 2025H laptop running 64-bit Windows 11, with an AMD Ryzen 9 8945HX processor, 32 GB of memory, a 2 TB solid-state drive, and an NVIDIA GeForce RTX 5070 Laptop GPU.

The evaluation used 29 CEC2017 functions, namely F1 and F3–F30; F2 is excluded from the suite. The functions comprise unimodal, multimodal, hybrid, and composition categories. F1 and F3 primarily assess local exploitation and final accuracy. Functions F4–F10 contain multiple local optima and assess global exploration and escape from local optima. Functions F11–F20 combine heterogeneous subfunctions and exhibit strong variable interactions. Functions F21–F30 use shifts, rotations, and groupings to create more complex fitness landscapes and assess overall search capability.

Because the CEC2017 functions have different bias and optimum values, the experiments report the raw fitness values returned by the benchmark program (Table 5). Lower fitness consistently indicates better performance.

### 5.2. Experiments and Analysis

This section first analyzes IWOA search behavior using search trajectories, mean population fitness, individual motion trajectories, exploration-exploitation ratios, and population diversity. It then compares IWOA with seven algorithms on 29 CEC2017 functions using mean ranks, Wilcoxon rank-sum tests, box plots, and convergence curves.

Figure 5 visualizes eight representative functions. For each function, it shows the three-dimensional landscape, population search history, mean population fitness, one-dimensional individual trajectories, IWOA convergence curve, and two-dimensional population distributions after 7500, 15,000, 22,500, and 30,000 function evaluations. These views respectively characterize the problem landscape, regions visited by the population, changes in overall population quality, individual motion over time, and improvement of the best fitness.

The search histories show that IWOA visited broad regions early in each run, when individuals were widely dispersed. As the number of function evaluations increased, the population gradually concentrated in low-fitness regions, while some individuals remained outside the current-best region. This pattern is consistent with the intended effects of good-point-based initialization, population migration, and Cauchy perturbation. However, isolating the contribution of each strategy requires an ablation analysis.

On F5, F8, F25, and F28, both mean and best population fitness decreased rapidly during the early stage, indicating rapid identification of promising regions. The range of the one-dimensional trajectories also narrowed over time, reflecting a transition from broad exploration to local exploitation. On F3, individual positions changed more gradually and the population retained a wider search range before concentrating. For the hybrid functions F12 and F18, the best-fitness curves improved in several steps during the middle and late stages, indicating that additional search mechanisms generated competitive candidates after an incumbent best solution had been found.

Overall, IWOA exhibited three search stages: broad early exploration, mid-stage selection of promising directions, and late-stage refinement around the best region. Stepwise improvements on complex functions during the middle and late stages indicate partial alleviation of late-stage stagnation.

To further examine the balance between global exploration and local exploitation, the corresponding ratios were calculated over the function-evaluation budget for the eight representative functions.

On F1, F5, F8, and F18, population diversity decreased rapidly during the early stage and then remained low but nonzero. IWOA therefore narrowed the search after identifying promising regions while retaining some differences among individuals. Diversity on F3 decreased more slowly and remained relatively high during the early and middle stages, indicating gradual screening of the search space rather than immediate concentration.

The F12 diversity curve showed several mid-stage rebounds and fluctuations that corresponded to changes in its exploration ratio. Some individuals therefore left the concentrated region and searched new locations. This behavior is consistent with population migration and Cauchy mutation, although their independent contributions require an ablation analysis. On F25 and F28, diversity decreased rapidly and remained low. IWOA ranked second on both functions, slightly behind SSA in mean fitness, indicating rapid convergence to high-quality regions but some remaining scope to improve late-stage exploitation.

Taken together, IWOA population diversity was high initially, decreased during the middle stage, and fluctuated at a low level during the late stage, as shown in Figure 6. This pattern is consistent with a transition from global exploration to local exploitation. Diversity recovery on some complex functions indicates an ability to resume broader search, whereas the rapid decline on other functions identifies a remaining limitation.

On F1, F5, and F8, the exploration ratio decreased rapidly during the early stage while the exploitation ratio increased and remained high. The algorithm therefore identified promising regions quickly and allocated more evaluations to local refinement. On F3, the transition was more gradual: exploration dominated initially, then decreased until exploitation became dominant during the middle stage. This transition may reduce the risk of premature concentration when the search space remains insufficiently explored.

The exploration ratio on F12 fluctuated and rebounded during the middle stage, indicating renewed global-search activity during local search. This behavior is consistent with population migration and Cauchy mutation, but the responsible mechanism requires ablation analysis. F18, F25, and F28 completed most exploration early, after which exploitation increased rapidly and late-stage search focused on identified high-quality regions.

Overall, IWOA shifted dynamically from global exploration to local exploitation. On some functions, however, exploration decreased rapidly, indicating a comparatively exploitation-oriented search. The exploration-exploitation ratio alone is therefore insufficient to establish that premature convergence was avoided; it must be interpreted together with population diversity and repeated-run statistics.

Figure 7 complements the population-diversity results in Figure 6 by separating the exploration and exploitation ratios. A high exploration ratio indicates broad search, whereas a rising exploitation ratio indicates increasing concentration around promising regions.

Across the 29 functions, IWOA achieved a mean rank of 1.38 and ranked first among the eight algorithms. It ranked first on 20 functions, second on seven, and third on two, placing within the top three for every function. The mean ranks of WOA, DBO, PSO, GWO, SSA, HHO, and ACO were 7.52, 5.34, 3.17, 3.66, 3.41, 7.07, and 4.45, respectively (Table 6).

IWOA obtained the lowest median on 20 of the 29 functions, indicating that most repeated-run results were concentrated in lower-fitness regions. In particular, both its mean and median were lower than those of the other algorithms on F3, F5, F7, F8, F11, F14, F17, F18, F21, F22, F24, and F29.

The median and the complete observed range in Table 6 provide a robust counterpart to the mean-based ranking. On F1, IWOA had a median of $1.37\times 10^{3}$, a mean of $8.45\times 10^{3}$, and a range from $1.04\times 10^{2}$ to $1.90\times 10^{5}$; the large mean–median separation confirms a right-skewed distribution with upper-tail outliers. On F13, ACO achieved a lower median than IWOA ($7.82\times 10^{3}$ versus $8.78\times 10^{3}$), although IWOA had the lower mean ($1.38\times 10^{4}$ versus $1.98\times 10^{4}$). On F19, SSA likewise achieved a lower median than IWOA ($4.37\times 10^{3}$ versus $5.40\times 10^{3}$), whereas IWOA had the lower mean ($7.12\times 10^{3}$ versus $1.15\times 10^{4}$). The comparison algorithms were therefore competitive on these functions, and the global mean rank should not be interpreted as uniform dominance. The archived result workbook contains the five reported descriptive statistics but not the 30 individual observations; consequently, an interquartile range cannot be reconstructed reliably and is not invented here.

IWOA obtained the lowest standard deviation on eight functions: F3, F7, F11, F13, F14, F15, F18, and F19. SSA and ACO showed lower dispersion on several other functions. IWOA’s principal advantage therefore lies in its combination of accuracy and mean rank, rather than uniformly low variability. The box plots, means, and medians show low fitness on most functions, but they also reveal dispersion and outliers on several functions. These results indicate that stability can be improved (Figure 8).

On F3, F5, and F8, IWOA reduced fitness rapidly during early iterations and maintained low values during the middle and late stages. This behavior is consistent with the intended roles of good-point-set initialization and adaptive search weighting, but the combined experiment does not isolate their individual causal contributions.

On the hybrid functions F12 and F18, the IWOA convergence curves improved in several steps after an incumbent best had been found. Population migration and Cauchy mutation were designed to support such renewed exploration; however, attribution to either mechanism requires the ablation study identified in the limitations. On F25 and F28, IWOA approached its final value relatively early, but SSA ultimately achieved slightly lower mean fitness. IWOA was therefore competitive on these functions, although its final local exploitation was less accurate than that of SSA.

On F1, the representative IWOA run reached a low fitness value, whereas the repeated-run results had a large standard deviation. A single convergence curve therefore illustrates only one search trajectory and cannot replace statistics from 30 independent runs. Algorithm performance must be assessed jointly from means, standard deviations, medians, box plots, and statistical tests.

Overall, IWOA converged rapidly during early iterations and achieved low final fitness on most representative functions. Stepwise middle- and late-stage improvements on complex functions indicate continued generation of useful search directions and partial mitigation of population concentration and stagnation, shown in Figure 9.

Table 7 reports pairwise Wilcoxon rank-sum tests between IWOA and the seven comparison algorithms on 29 functions at a significance level of 0.05. Of the 203 comparisons, 188 had *p* < 0.05 (92.61%), indicating different result distributions for most function-algorithm pairs. Together with the mean ranks and descriptive statistics, these tests support IWOA’s overall advantage on most functions. Statistical significance alone, however, does not establish that IWOA outperformed every algorithm on every function; the mean, median, and rank for each function must also be considered.

## 6. Application and Simulation Analysis of IWOA for Three-Dimensional UAV Path Planning

### 6.1. Experimental Setup

A digital elevation model (DEM) was used to construct three-dimensional mission environments containing radar threats, other threats, and no-fly zones. The single- and multi-UAV evaluations each used four map–complexity combinations, yielding eight scenarios (Figure 10).

IWOA was evaluated in repeated single-UAV and cooperative multi-UAV experiments. The single-UAV study used ten independent runs per algorithm and scenario. In the legacy cooperative study, each of eight algorithms was run 30 times in four scenarios. Every run used a population of 30, 500 iterations, and the same 230-dimensional encoding, yielding 960 completed runs. A separate feasibility-priority experiment compared IWOA-FP and WOA-FP across the same four scenarios, using 30 paired seed labels, a population of 30, and 500 iterations. This experiment yielded another 240 completed runs. Both feasibility-priority algorithms received the same scenario-specific validated incumbent as one initial population member, and no formal run was excluded. Selection scores were recomputed from every saved final solution and agreed within the stored numerical precision. We first report feasibility counts and Wilson 95% intervals. We then report the mean, standard deviation, median, and IQR of the implemented composite objective among feasible final solutions. Two-sided paired Wilcoxon signed-rank tests used an asymptotic normal approximation with tie and continuity corrections, and Holm adjustment was applied to the four feasibility-priority comparisons. The comparisons control population size and iteration count, but not the number of objective evaluations. WOA uses 15,030 evaluations per run, whereas IWOA uses 30,530 because each iteration additionally evaluates opposition-based and Cauchy candidates. The results are therefore fixed-generation comparisons and do not establish equal-function-evaluation efficiency.

UAV paths were represented using spherical increments. Each variable group describes the distance and direction from one waypoint to the next, aligning the decision variables with motion between adjacent points and reducing path reversals and disordered waypoint distributions relative to direct Cartesian-coordinate optimization. In the multi-UAV experiments, the path and speed parameters of all UAVs were combined in one decision vector to optimize spatial paths and flight times jointly.

Seven population-based algorithms were compared with IWOA: WOA, DBO, PSO, GWO, SSA, HHO, and ACO. WOA provides a fixed-generation reference for the complete production IWOA implementation. However, the two implementations differ in both their operator sets and numbers of objective evaluations. The comparison therefore does not isolate the combined effect of the four highlighted mechanisms. DBO and SSA are more recent swarm-intelligence methods, whereas PSO, GWO, and ACO are established algorithms. HHO combines global exploration with rapid local exploitation. Within each scenario, all algorithms used identical objective functions, variable bounds, and generation limits, shown in Figure 11.

To keep the evaluated configurations distinct, the implementation map below links each name to its evidence track. The production and feasibility-priority IWOA routines retain the integrated operators described in Section 4; the modular variants use the switchable common backbone defined only for the factor screen.
**Name****Evidence Track****Objective and Selection****Initialization and Active Procedure**Production IWOACEC2017, single-UAV, and legacy cooperative comparisons$F_{\mathrm{impl}}$ with penalty-only rankingDeterministic coordinate-wise initialization; integrated IWOA procedure in Section 4Modular WOA variantsControlled factor screen in M4-C2$F_{\mathrm{impl}}$ with penalty-only rankingOne common WOA backbone with GPS, AW, PM, and CM switched individually or jointlyIWOA-FPFeasibility-priority comparison$F_{FP}$ for selection; $F_{\mathrm{impl}}$ reported conditional on feasibilityOne shared validated incumbent plus the production IWOA population/update procedureWOA-FPFeasibility-priority comparison$F_{FP}$ for selection; $F_{\mathrm{impl}}$ reported conditional on feasibilityOne shared validated incumbent plus uniform initialization and standard WOA updates

### 6.2. Implementation Tools and Control-System Integration

The experiments were implemented as MATLAB scripts using double-precision numerical calculations on the Windows 11 workstation specified in Section 5.1. The simulation package separates environment construction, spherical-to-Cartesian decoding, objective evaluation, algorithm updates, B-spline visualization, path plotting, and spreadsheet export. The UAV samples are simulated mission instances rather than measurements from physical airframes: each instance contains a DEM, threat and no-fly-zone parameters, start and goal coordinates, departure and expected-arrival times, speed and attitude limits, and 15 intermediate waypoints per UAV. No hardware-in-the-loop or real-flight data are included.

When integrated with an existing UAV control system, IWOA should operate as an offline mission-planning module above the flight-control layer. A ground-control or mission-management system supplies the static map, geofences, vehicle limits, start and goal states, and timing requirements. IWOA returns an ordered waypoint–speed schedule for each UAV. A separate validation gate then checks the unsmoothed path and, when used, the densely sampled smoothed path against all spatial, kinematic, timing, and pairwise-separation constraints. Only a validated schedule should be converted into the waypoint format accepted by the flight controller. During execution, the onboard controller remains responsible for trajectory tracking, state estimation, emergency handling, and local avoidance. The offline planner does not replace these safety-critical functions.

### 6.3. Single-UAV Planning

Figure 12 summarizes the paths and convergence curves of the eight algorithms. Table 8 reports the mean fitness and standard deviation over ten independent runs. Single-UAV performance was evaluated jointly from path geometry, convergence behavior, and repeated-run statistics.

The enlarged panels make the convergence comparison explicit. In M1-C1 and M1-C2, IWOA descends rapidly during the first iterations and remains among the lowest curves. In the two M2 scenarios, it continues to improve after the initial descent and reaches the lowest or nearly lowest terminal cost. These curves visualize one trajectory per scenario and are interpreted together with the ten-run mean and standard deviation in Table 8 rather than as stand-alone statistical evidence.

All algorithms generated candidate paths connecting the start and target points, although their smoothness differed in the plan and three-dimensional views. Path plots alone do not show all penalties for threats, angular constraints, and minimum segment lengths. They must therefore be interpreted together with the composite fitness and path-quality statistics.

Compared with the M1 scenarios, IWOA’s mean-fitness advantage over most comparison algorithms was more pronounced in the more complex M2-C1 and M2-C2 scenarios.

As shown in Table 8, IWOA achieved the lowest mean fitness in M1-C2, M2-C1, and M2-C2 and ranked second in M1-C1. Its mean rank across the four scenarios was 1.25. Although IWOA did not obtain the best individual result in every scenario, the ten independent runs indicate stronger overall cross-scenario search quality rather than isolated extreme performance.

### 6.4. Cooperative Multi-UAV Planning

Table 1 and Table 2 summarize the 30 independently seeded, 500-iteration runs per algorithm and scenario. IWOA had the lowest median final fitness in M3-C1, M3-C2, and M4-C2, whereas SSA had the lowest median in M4-C1. The mean results were less favorable: ACO had the lowest mean in M3-C1 and M4-C2, and SSA had the lowest mean in M3-C2 and M4-C1. This difference between mean and median is caused by large penalty-triggered outliers and is retained rather than hidden. Across the four scenarios, the mean Friedman ranks were 2.33 for ACO, 2.50 for IWOA, and 2.65 for SSA; all four scenario-wise Friedman tests rejected equal performance ($p<1.6\times 10^{-21}$).

Holm-adjusted paired Wilcoxon tests further delimit the legacy penalty-only result. IWOA outperformed six of seven baselines in M3-C1 (all except ACO) and M3-C2 (all except SSA). In M4-C1, IWOA was worse than SSA, better than HHO, and not detectably different from the remaining five algorithms after adjustment. In M4-C2, IWOA outperformed WOA, DBO, PSO, and HHO, but was not detectably different from GWO, SSA, or ACO. All proportions passing the legacy post hoc screen were 0. Consequently, this experiment supports competitive typical final fitness in three scenarios but exposes a constraint-handling failure; it does not support a feasibility claim. Section 6.6 reports the targeted feasibility-priority correction.

Figure 13 and Figure 14 retain the archived representative three-dimensional and top-view trajectories for the eight algorithms; they are illustrative examples and were not selected from, or used as the statistical basis for, the 30-run comparison. Different UAVs are shown in different colors, start and target points use consistent markers, and radar threats, other threats, and no-fly zones use distinct low-saturation colors. Spatial plots alone cannot establish temporal coordination or constraint feasibility. The 0/120 legacy IWOA screen result is therefore reported explicitly and is not inferred away from visual appearance; the corrected feasibility-priority experiment is evaluated numerically in Table 9 and Figure 15.

Figure 1 and Figure 2 make the stability limitation visible. IWOA’s median curve is competitive in M3-C1, M3-C2, and M4-C2, but its wide IQR in M4-C1 and M4-C2 shows that some runs enter high-penalty regions. Reporting only a selected trajectory would conceal this behavior. The raw run-level data, recomputation checks, and statistical tables are supplied in the accompanying source-data workbook.

### 6.5. Controlled Modular Factor Screen

To provide a limited factor-screening comparison with minimal additional experimentation, a modular WOA-backbone study was performed in the difficult M4-C2 scenario. The modular WOA reference, WOA plus good-point-set initialization (GPS), WOA plus adaptive weight (AW), WOA plus population migration (PM), WOA plus Cauchy mutation (CM), and WOA with all four strategies (All4) were each evaluated for 30 paired seeds using populations of 30 and 500 iterations. The variants share one modular backbone, but the added operators trigger additional objective evaluations; realized counts range from 15,030 to 25,530 per run. Moreover, the modular backbone and operator implementations are not numerically identical to the legacy production WOA/IWOA routines used in the main comparison, and the production routine includes additional pooling, preferential-selection, DFDB, and SOBL mechanisms. This experiment is therefore a fixed-generation factor screen, not an equal-function-evaluation causal ablation of the production IWOA code.

Under these fixed-generation settings, All4 had 35.0% lower median fitness and 36.6% lower mean fitness than the modular WOA reference, with a positive paired association ($p_{\mathrm{Holm}}=0.0093$, rank-biserial correlation =0.639). PM and CM also showed adjusted paired associations, whereas GPS and AW alone did not reach the adjusted threshold. Unequal objective-evaluation counts, broad IQRs, and the 0 proportion passing the limited post hoc screen for every variant prevent a causal efficiency claim or a reliable-feasibility claim.

### 6.6. Feasibility-Priority Validation Experiment

The feasibility-priority experiment directly addresses the failure of the legacy penalty-only search to satisfy its own reported constraint screen. IWOA-FP and WOA-FP were evaluated with 30 paired seed labels in each of the four 230-dimensional scenarios, using population 30 and 500 iterations. Both algorithms started each run with the same scenario-specific validated feasible incumbent as one initial population member and used Equation (94) for selection. All 240 returned terminal incumbents had $CV_{T}=CV_{A}=CV_{S}=CV_{C}=CV_{N}=0$, and all saved selection scores agreed with post-run recomputation within stored numerical precision. Thus, the modeled-feasibility rate was 30/30 for every algorithm–scenario group (100%; Wilson 95% CI 88.65–100%) and 120/120 for each algorithm overall. These counts demonstrate preservation and improvement of a feasible incumbent, not feasibility of every population member or random-start feasible-solution discovery; the Wilson intervals therefore describe the observed terminal-incumbent outcomes under this warm-start protocol and are not discovery-probability estimates.

Under the corrected modeled constraints, IWOA-FP and WOA-FP were statistically indistinguishable in M3-C1. IWOA-FP obtained a lower objective in M4-C1 and M4-C2, whereas WOA-FP performed better in M3-C2. This mixed result precludes a blanket superiority claim. The 100% terminal rate follows from a common feasible incumbent and feasibility-priority elitism. It therefore demonstrates feasible-plan preservation and optimization within the tested model, but it does not estimate random-start feasibility discovery. The validator also excludes DEM clearance, complete timing acceptance, smoothed-curve clearance, dynamics, wind, sensing uncertainty, and real-flight execution. The result is therefore not a flight-safety certificate.

## 7. Conclusions

This study integrated joint spherical-increment path–speed encoding with a multi-strategy whale optimizer and an explicit feasibility-priority evaluation branch. The representation adjusts path geometry, travel time, and pairwise separation within a 230-dimensional continuous decision vector. The principal methodological contribution is separating objective optimization from modeled-feasibility assessment, thereby preventing attractive penalty scores from being interpreted as evidence of constraint satisfaction.

Under fixed-generation testing, IWOA achieved a mean rank of 1.38 over 29 CEC2017 functions and 1.25 over four repeated single-UAV scenarios. In the 960-run legacy cooperative comparison, it achieved the lowest median final fitness in three scenarios but the lowest mean in none, and no algorithm produced a solution passing the legacy post hoc screen. In the separate feasibility-priority study, IWOA-FP and WOA-FP each preserved modeled feasibility in 120/120 terminal incumbents. Conditional objective results favored IWOA-FP in M4-C1 and M4-C2, WOA-FP in M3-C2, and neither method in M3-C1. These comparisons use equal generation limits but unequal objective-evaluation counts and therefore do not establish computational-efficiency superiority.

The central result is that penalty weighting alone was insufficient for the stated planning constraints, whereas feasibility-priority selection preserved a validated feasible plan throughout optimization. This finding supports offline mission preplanning but remains conditional on a shared feasible warm start and five modeled checks. It establishes neither unseeded feasibility discovery nor flight safety. Practical deployment therefore requires comprehensive independent validation, flight-controller-compatible waypoint conversion, onboard tracking, local avoidance, and emergency safeguards.

### 7.1. Practical Recommendations

For practitioners, the proposed method should be used for offline planning in mapped static environments, not as a stand-alone real-time collision-avoidance controller. All candidate algorithms should share the same objective, bounds, stopping rule, random-run protocol, and, when efficiency is compared, the same objective-evaluation budget. The returned waypoint–speed schedule should be accepted only after an independent validator checks every spatial, terrain, no-fly-zone, kinematic, timing, smoothing, and continuous-time separation requirement; zero reported violation components are necessary but not sufficient. If B-spline smoothing is applied, dense resampling and a full constraint recheck are mandatory; otherwise, the validated piecewise-linear path should be retained. During flight, local avoidance and emergency logic should remain active, and replanning should be triggered when map, weather, communication, or vehicle-state deviations invalidate the static planning assumptions.

### 7.2. Limitations

Despite the expanded experiment, several limitations remain. First, the 230-dimensional multi-UAV objective produces strongly skewed and sometimes bimodal outcomes. IWOA had the lowest median in three scenarios but no lowest mean, and its IQR was wide in the M4 cases. Thirty runs support the reported nonparametric comparisons, but they do not remove the observed instability or establish behavior beyond the tested maps, five-UAV scale, population size, and 500-iteration budget.

Second, none of the 960 legacy runs passed the four-count post hoc screen. The feasibility-priority validator adds wrapped-angle, segment-to-threat, plan-view no-fly-zone, minimum-segment, and continuous-time separation checks. It does not yet include complete DEM clearance, timing acceptance, smoothed-curve validation, vehicle dynamics, or uncertainty. The 100% terminal-incumbent result is also conditional on inserting one validated feasible plan per scenario and therefore measures preservation rather than random-start discovery.

Third, the factor screen uses one modular WOA backbone in one difficult scenario, and its variants have unequal objective-evaluation counts. It therefore provides paired fixed-generation associations rather than a complete causal or efficiency decomposition. The study is further limited to known static environments and does not model moving obstacles, wind, positioning errors, communication delays, or full vehicle dynamics. The displayed B-spline curves were not subjected to complete dense post-smoothing checks, and no hardware-in-the-loop or real-flight validation was performed.

### 7.3. Future Work

Future work should evaluate feasibility discovery without a supplied incumbent, including constructive initialization and repair for terrain clearance, threat/no-fly-zone crossings, angular and minimum-segment violations, timing, and pairwise separation. Equal-objective-evaluation comparisons should then test whether IWOA’s additional candidates improve efficiency within the feasible set. A component-switchable implementation would also permit every mechanism and important interaction to be evaluated on the same WOA backbone.

The reference validator should independently add DEM clearance, timing acceptance, smoothed-path checking, and vehicle-dynamic limits to the corrected threat, no-fly-zone, angular, segment-length, and continuous-time separation checks used here. B-spline paths should be resampled densely and rechecked against every spatial, kinematic, timing, and pairwise-separation constraint before export.

Finally, future extensions should address dynamic and uncertain environments by incorporating moving obstacles, weather disturbances, communication delays, and UAV dynamic constraints. Scalable planning methods are also needed for larger numbers of UAVs and waypoints. Parallel evaluation, professional UAV simulators, hardware-in-the-loop systems, and real-flight tests should quantify computation time, path-tracking accuracy, safety margins, and engineering value.

## Figures and Tables

**Figure 1:**
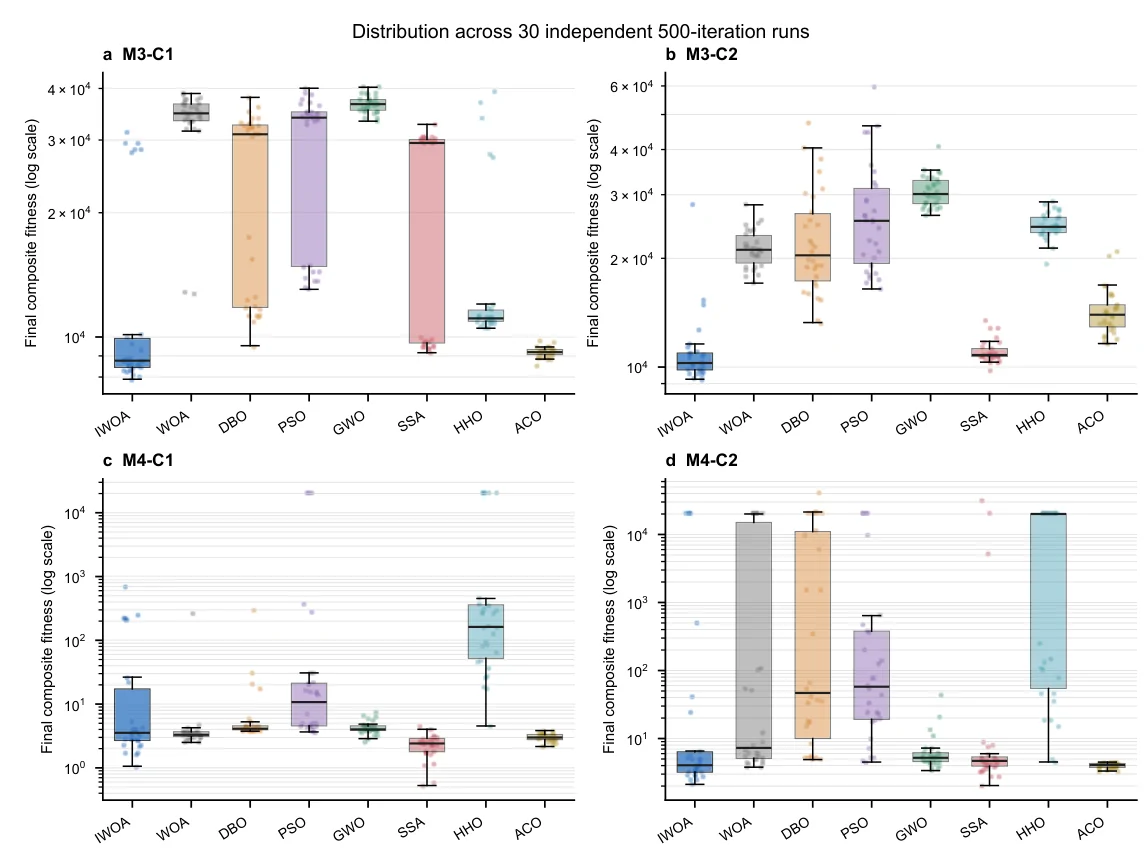
Final composite-fitness distributions for 30 independent 500-iteration runs in each cooperative scenario. Boxes show the median and interquartile range, whiskers extend to 1.5 IQR, and all runs are overlaid. The logarithmic axis exposes penalty-triggered outliers without excluding them.

**Figure 2:**
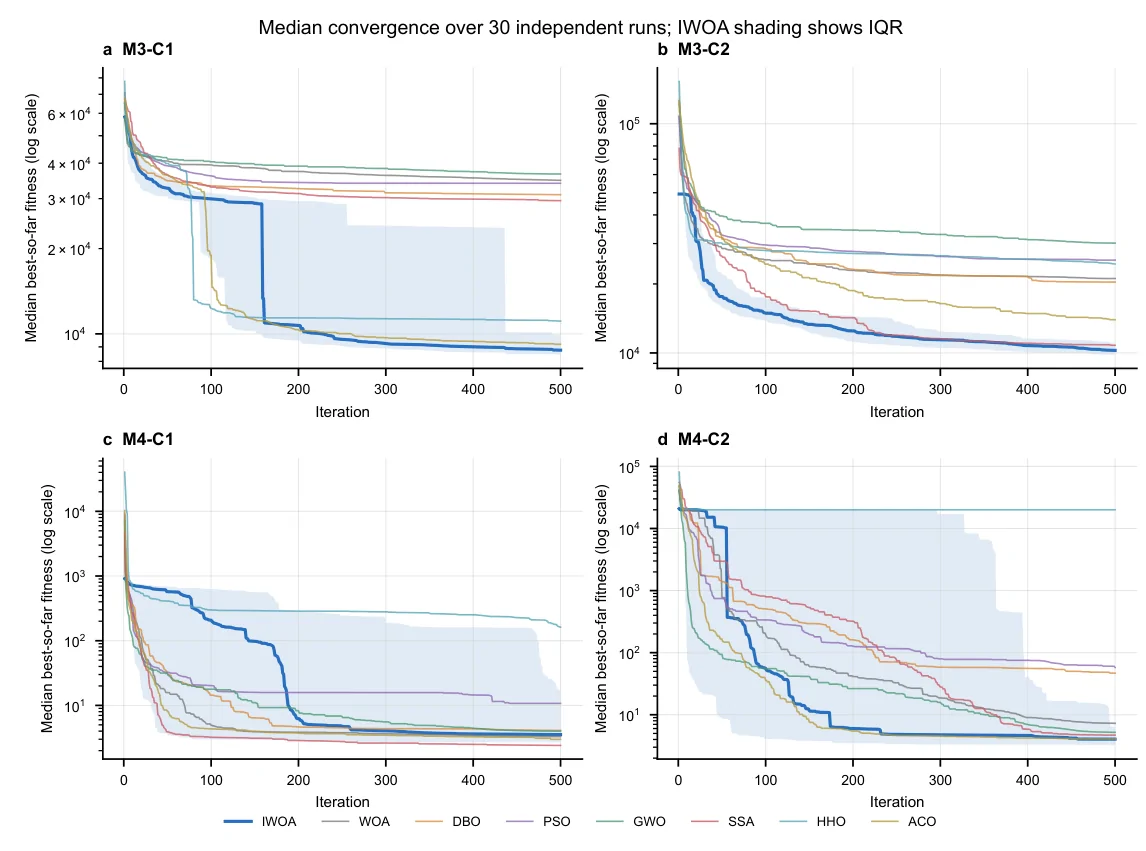
Median best-so-far convergence over 30 independent runs. The blue line denotes IWOA, and its shaded band denotes the IQR; comparator lines show their medians. The curves use the full 500-iteration budget.

**Figure 3:**
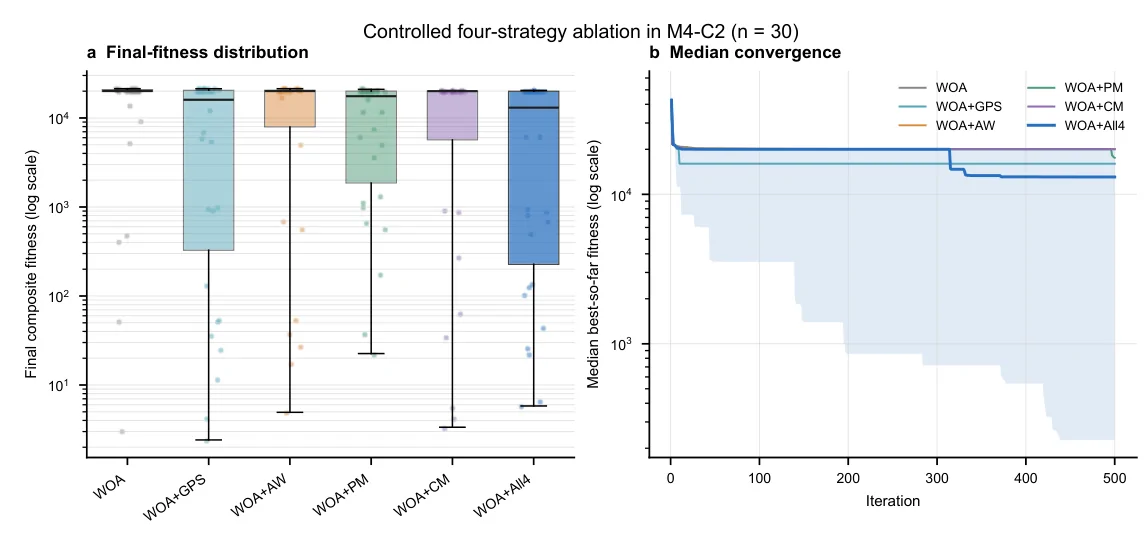
Controlled four-strategy modular WOA-backbone factor screen in M4-C2. (**a**) Final fitness distributions with every run shown. (**b**) Median best-so-far convergence; the shaded band is the IQR for WOA + All4.

**Figure 4:**
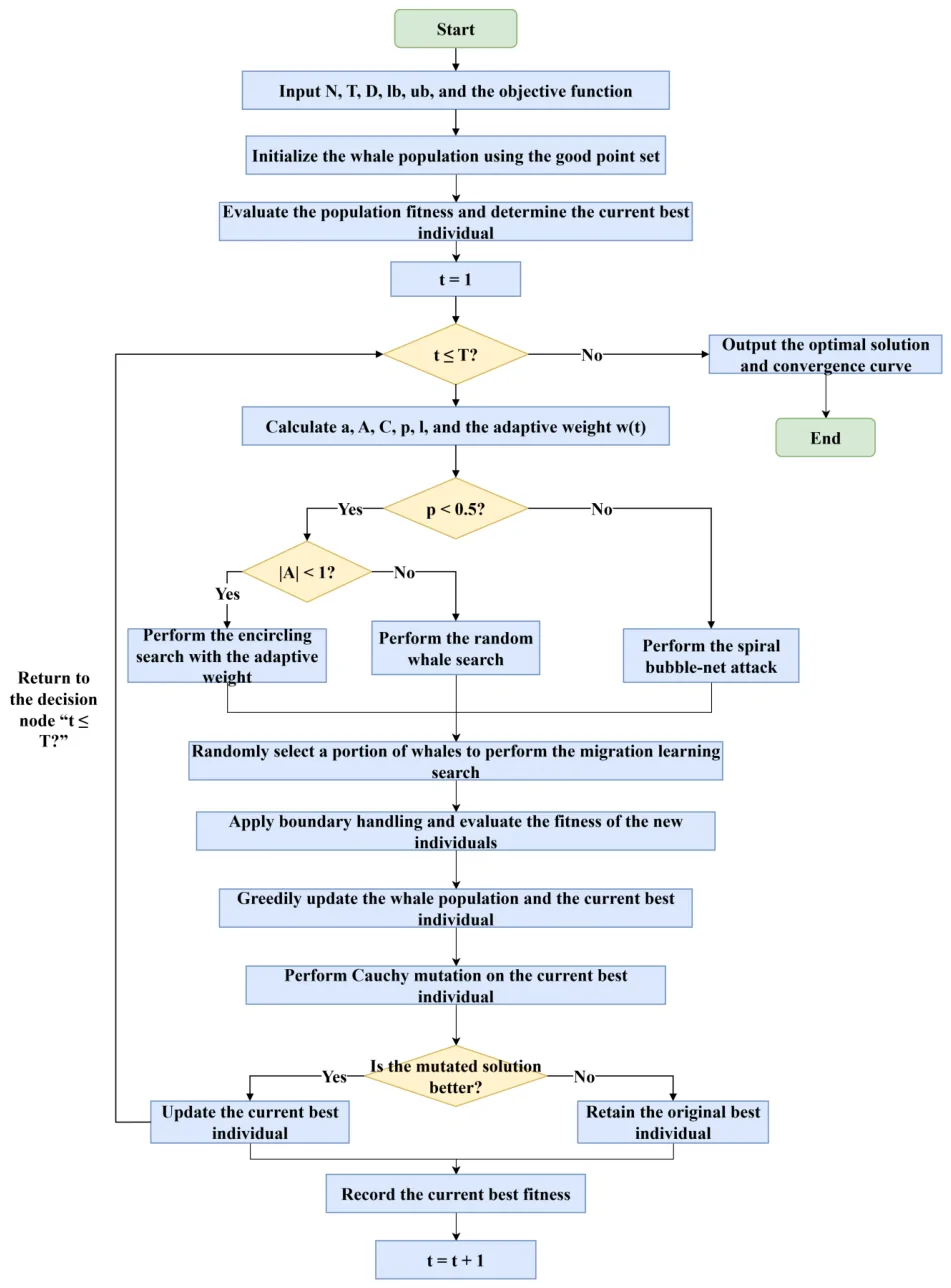
Flowchart of IWOA.

**Figure 5:**
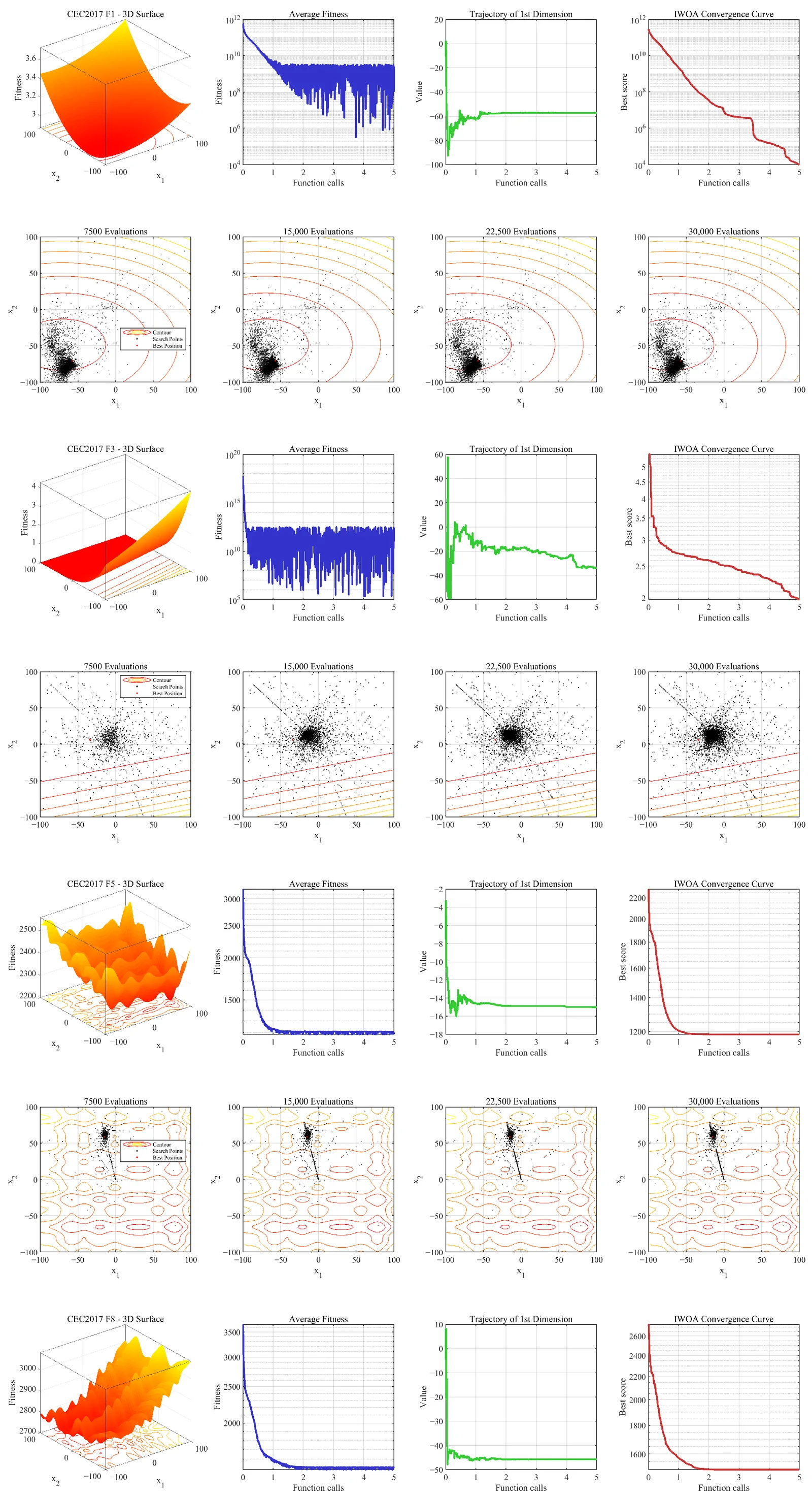

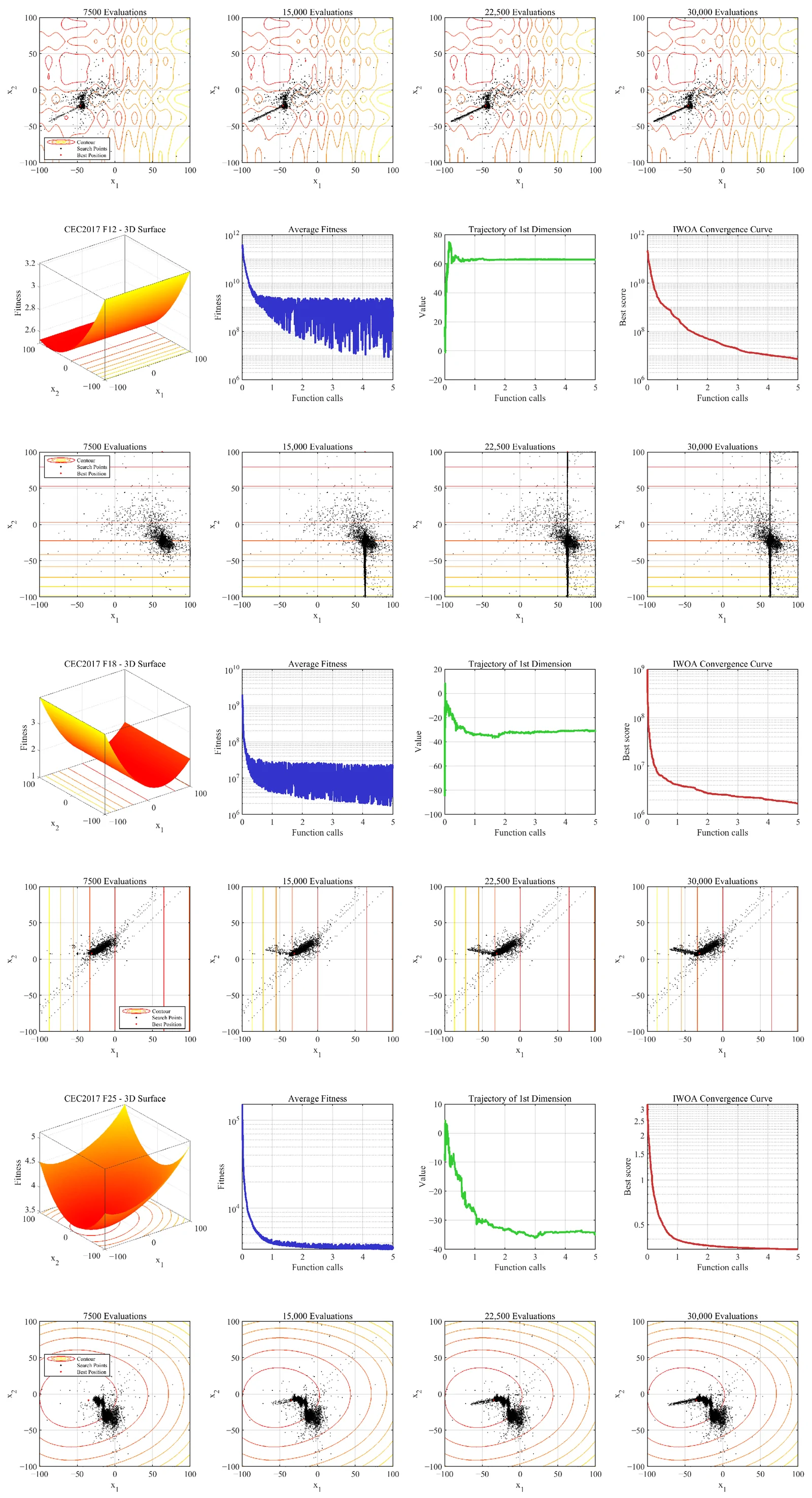

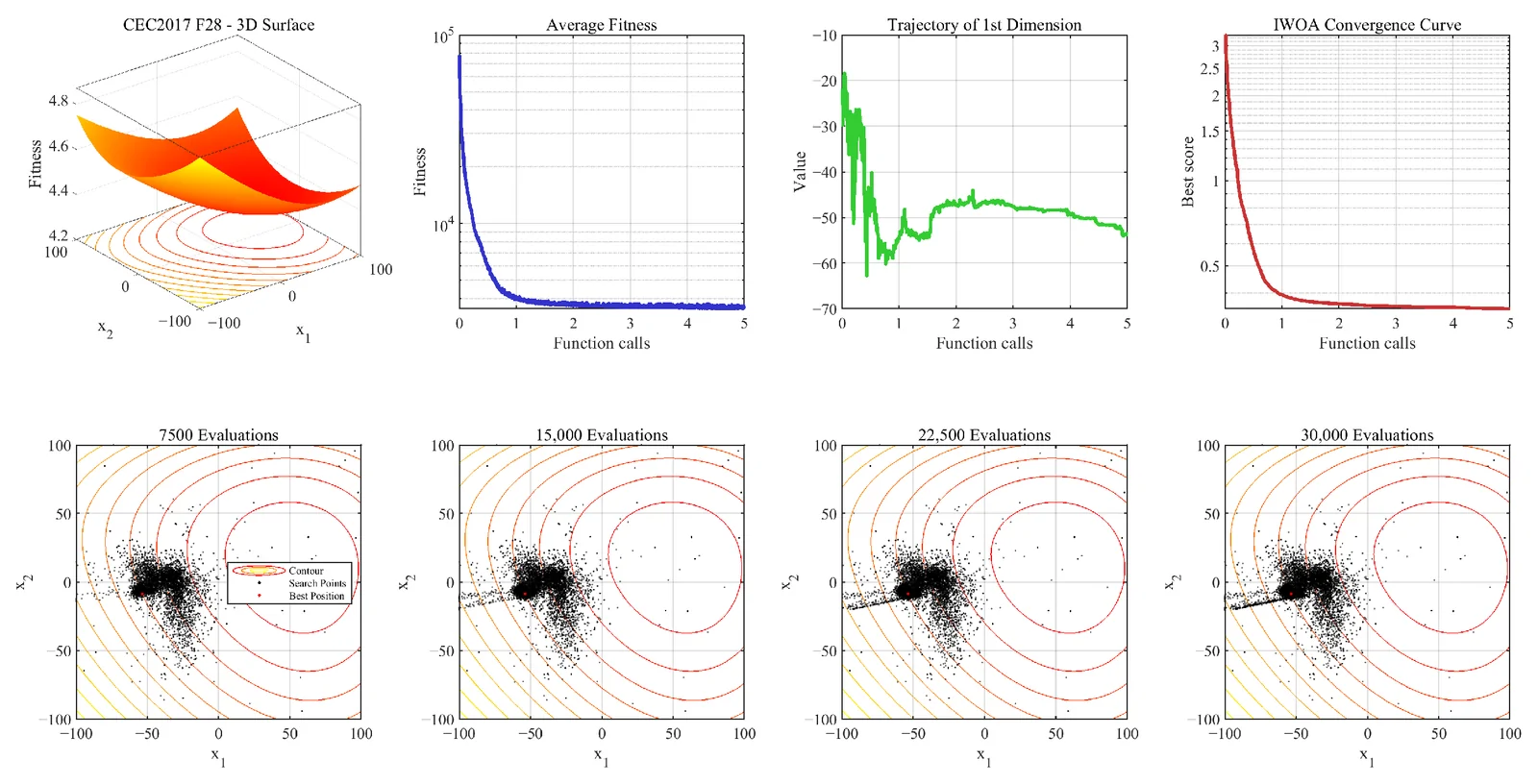
Search behavior of IWOA on eight representative CEC2017 functions.

**Figure 6:**
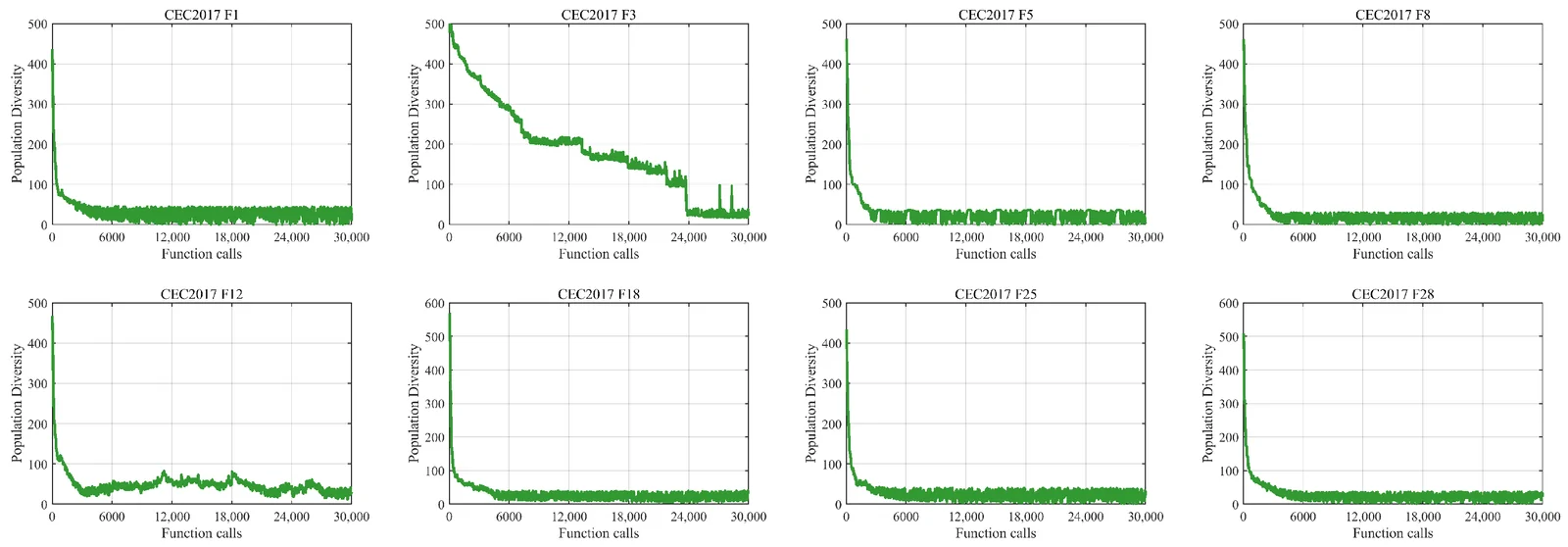
Population diversity of IWOA on eight representative CEC2017 functions.

**Figure 7:**
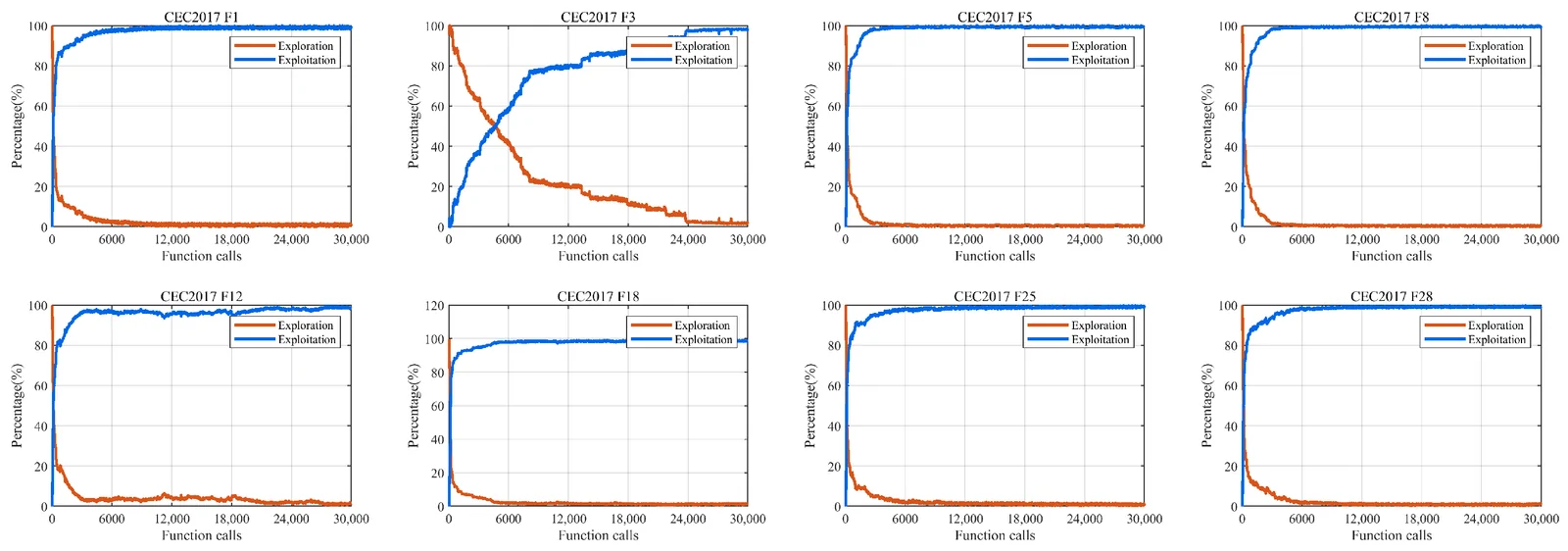
Exploration-exploitation ratio of IWOA on eight representative CEC2017 functions.

**Figure 8:**
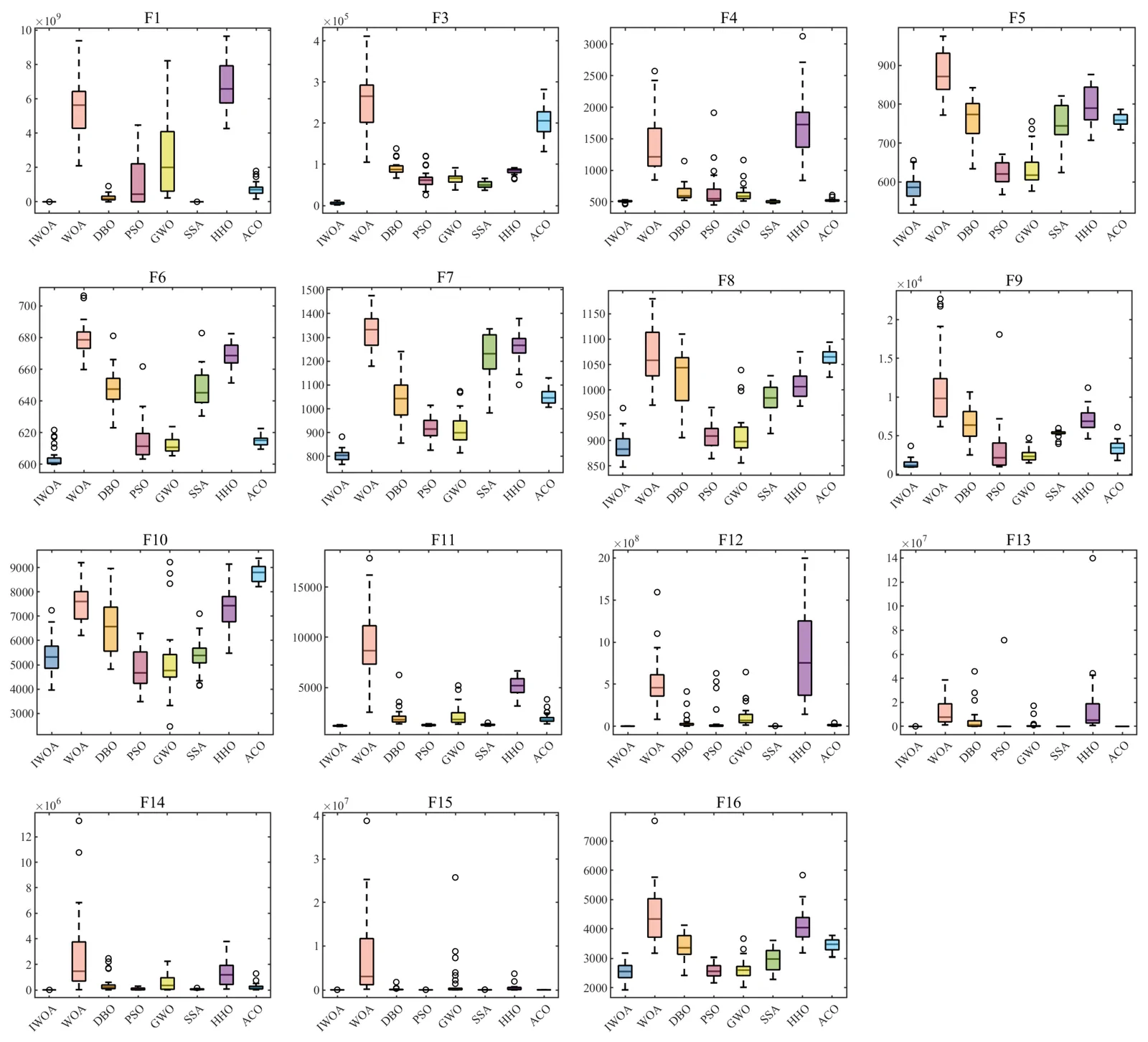

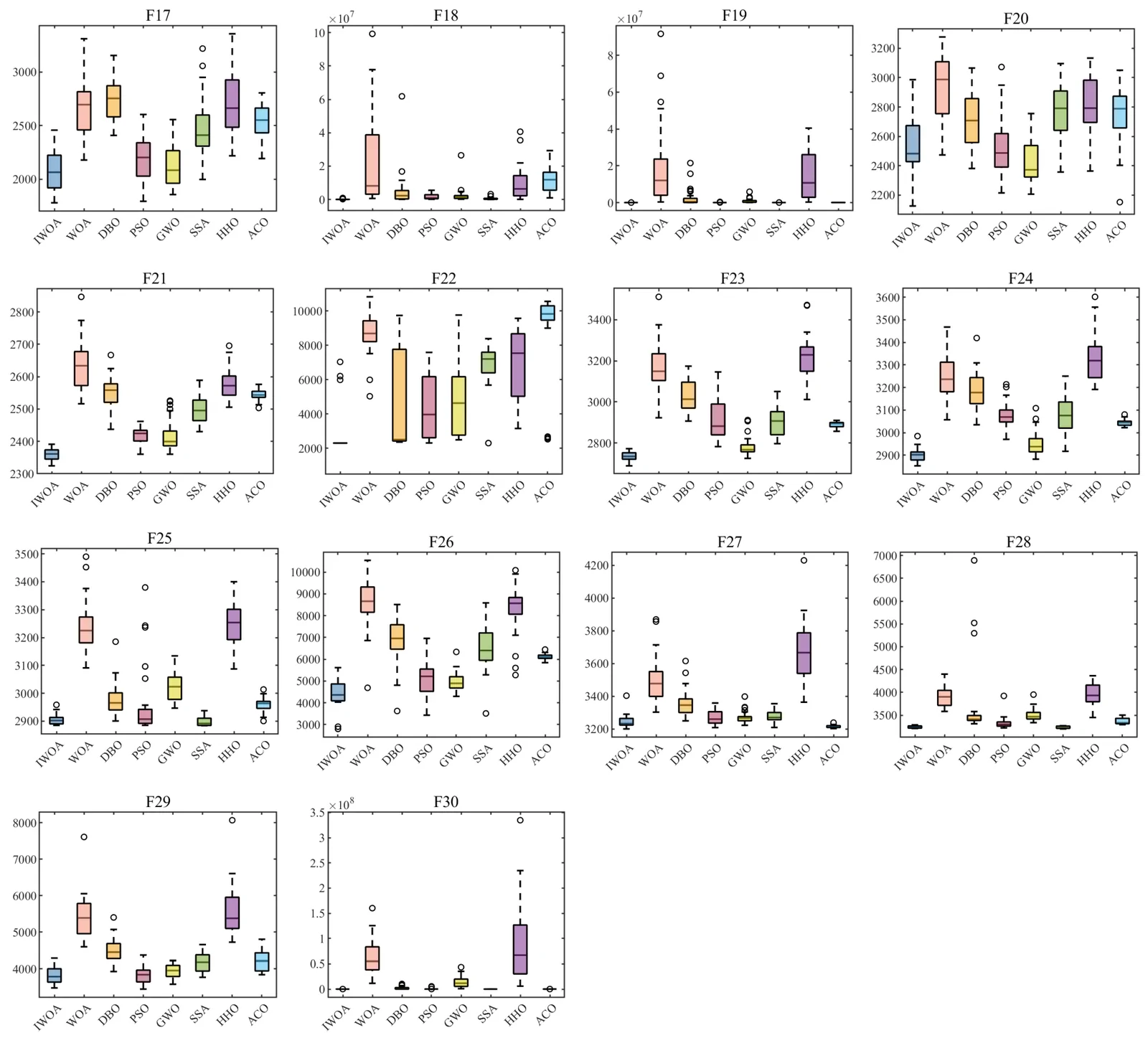
Enlarged box plots for eight algorithms on 29 CEC2017 functions, reassembled from the original plots without altering the data: (**upper panel**), F1 and F3–F16; (**lower panel**), F17–F30. The center line denotes the median, each box shows the interquartile spread represented in the archived plot, and whiskers/outliers show the plotted distribution.

**Figure 9:**
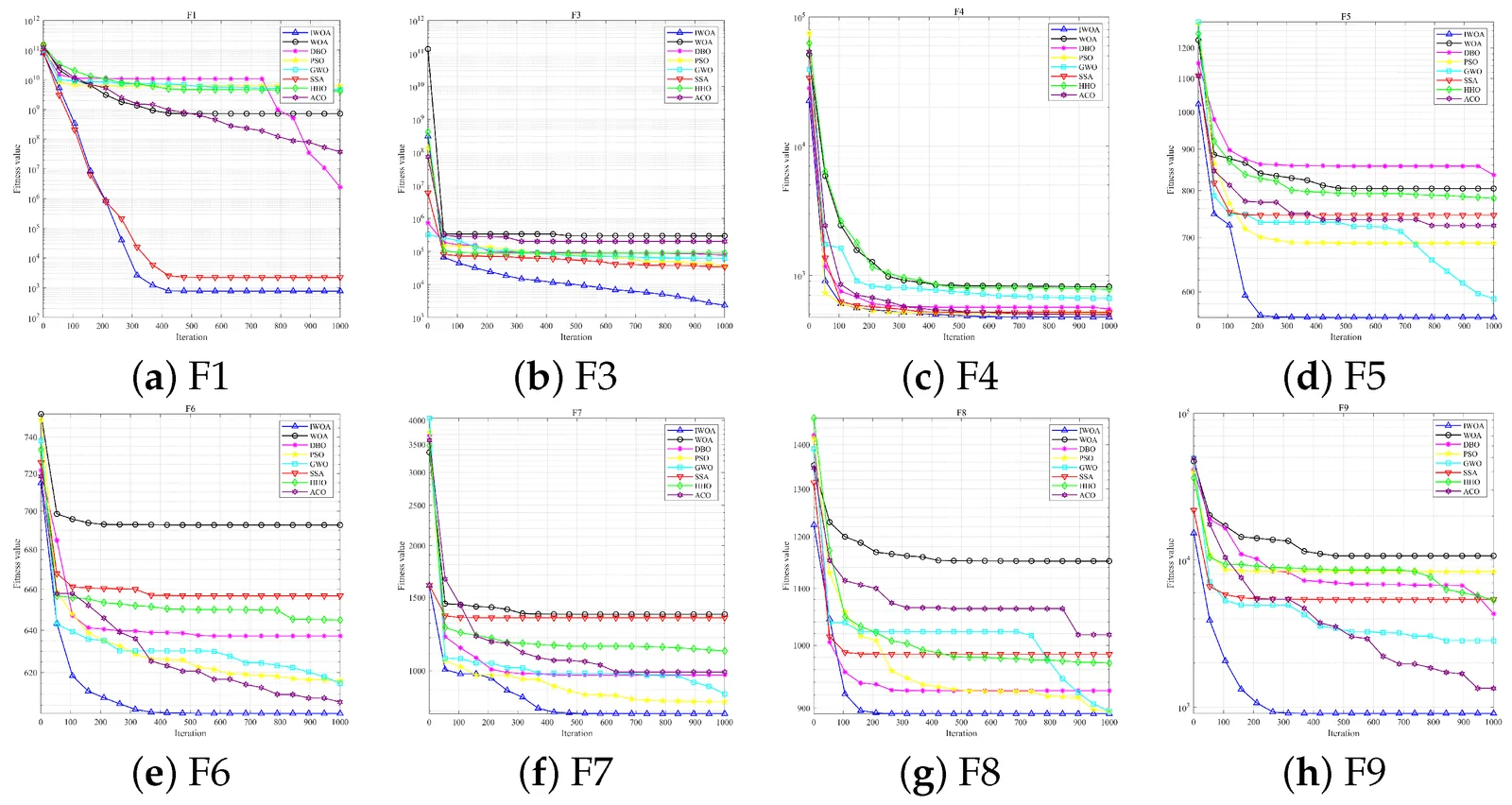

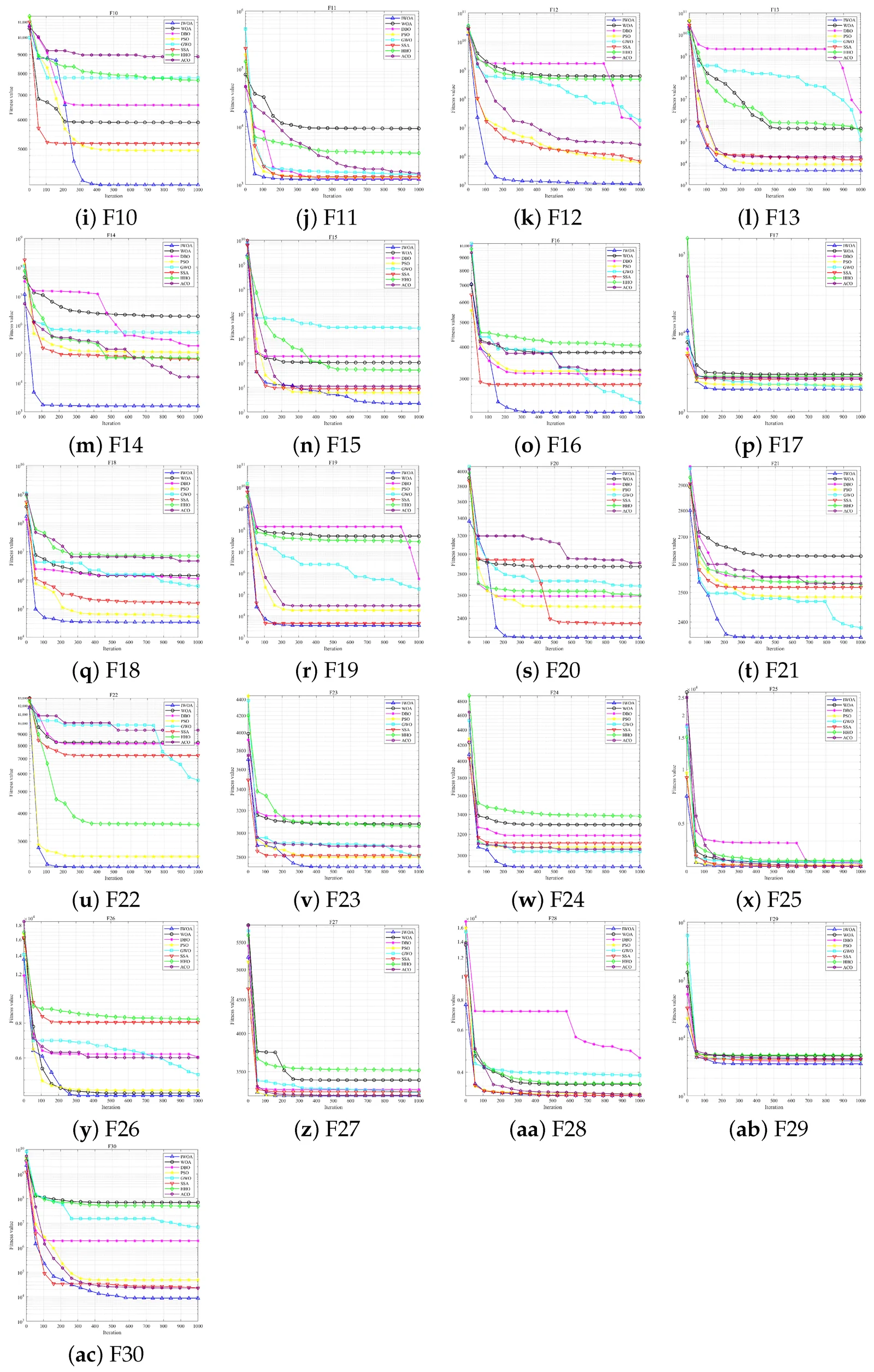
Convergence curves of eight algorithms on 29 CEC2017 functions. A four-column layout replaces the previous five-column plate so that axes, legends, and curve differences remain legible at manuscript scale.

**Figure 10:**
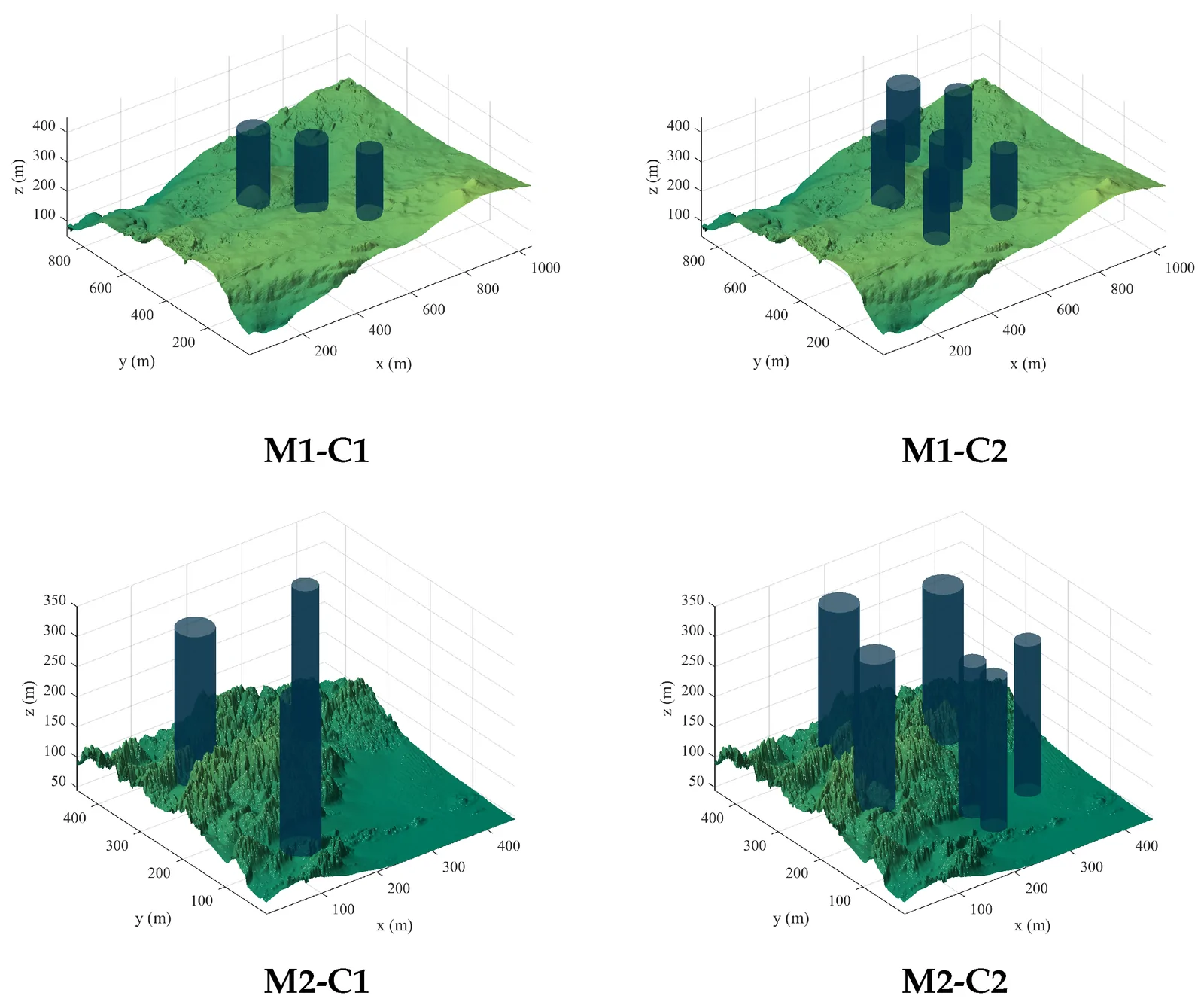
Single-UAV scenarios. Panels are ordered M1-C1, M1-C2, M2-C1, and M2-C2 from left to right and top to bottom; M denotes the map and C the complexity setting.

**Figure 11:**
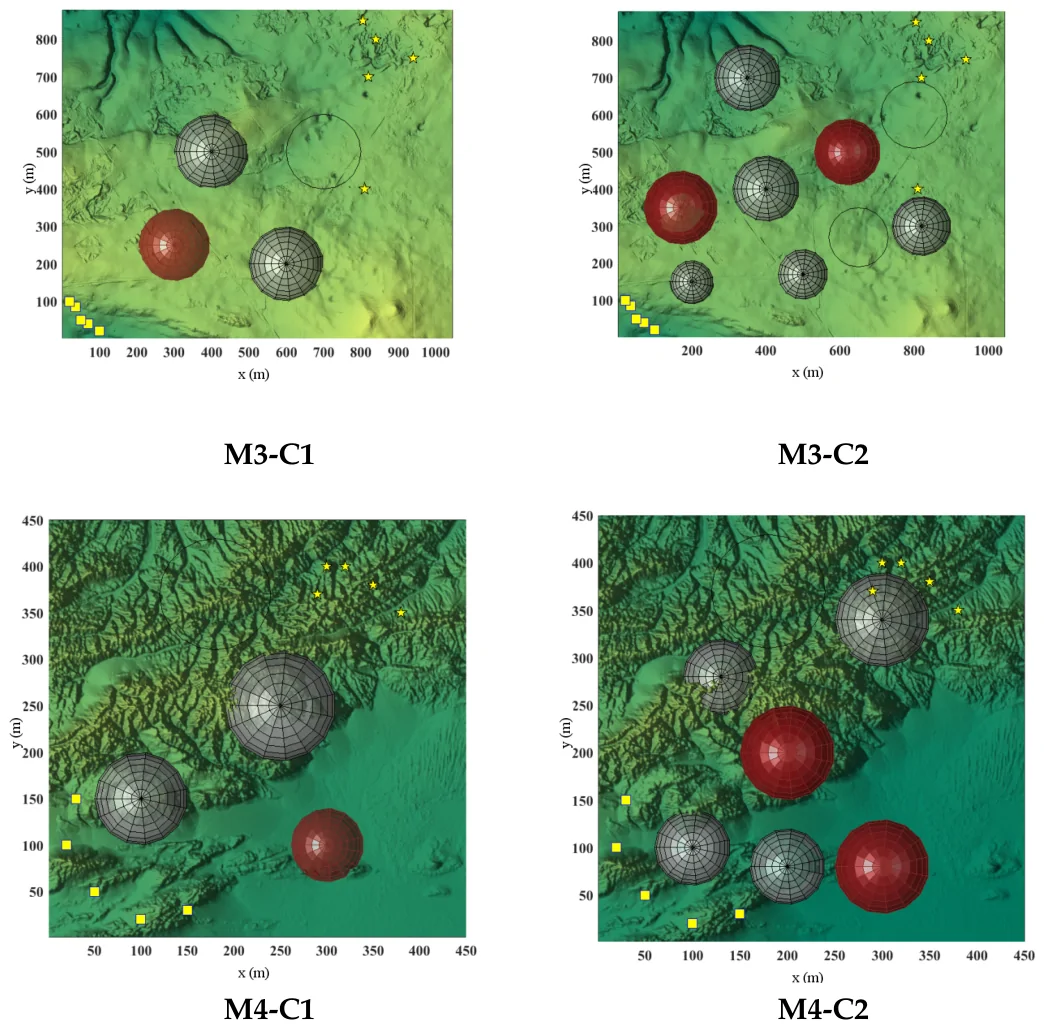
Cooperative multi-UAV scenarios. Panels are ordered M3-C1, M3-C2, M4-C1, and M4-C2 from left to right and top to bottom; the same identifiers are used in Table 1, Table 2 and Table 3.

**Figure 12:**
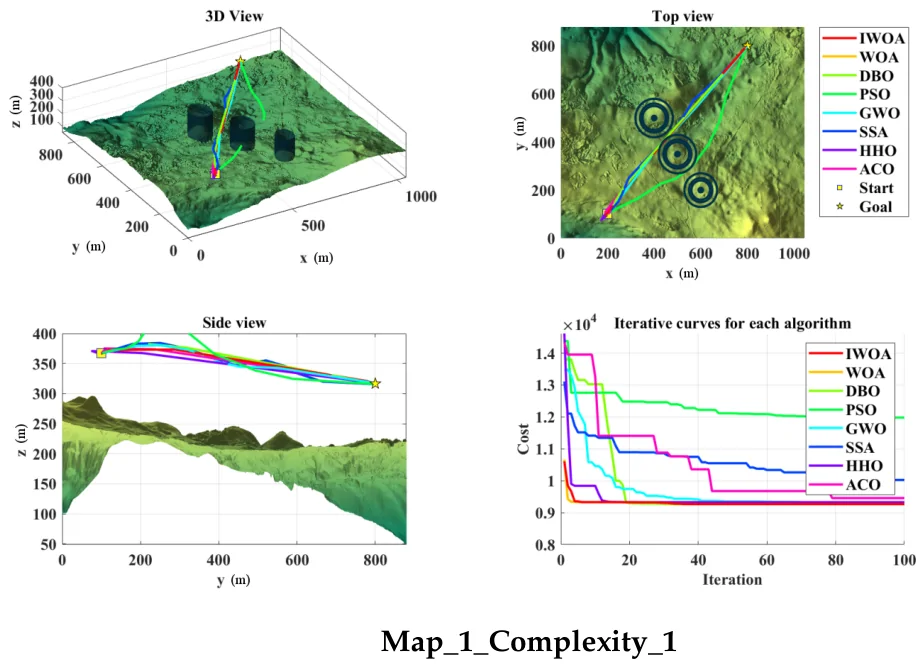

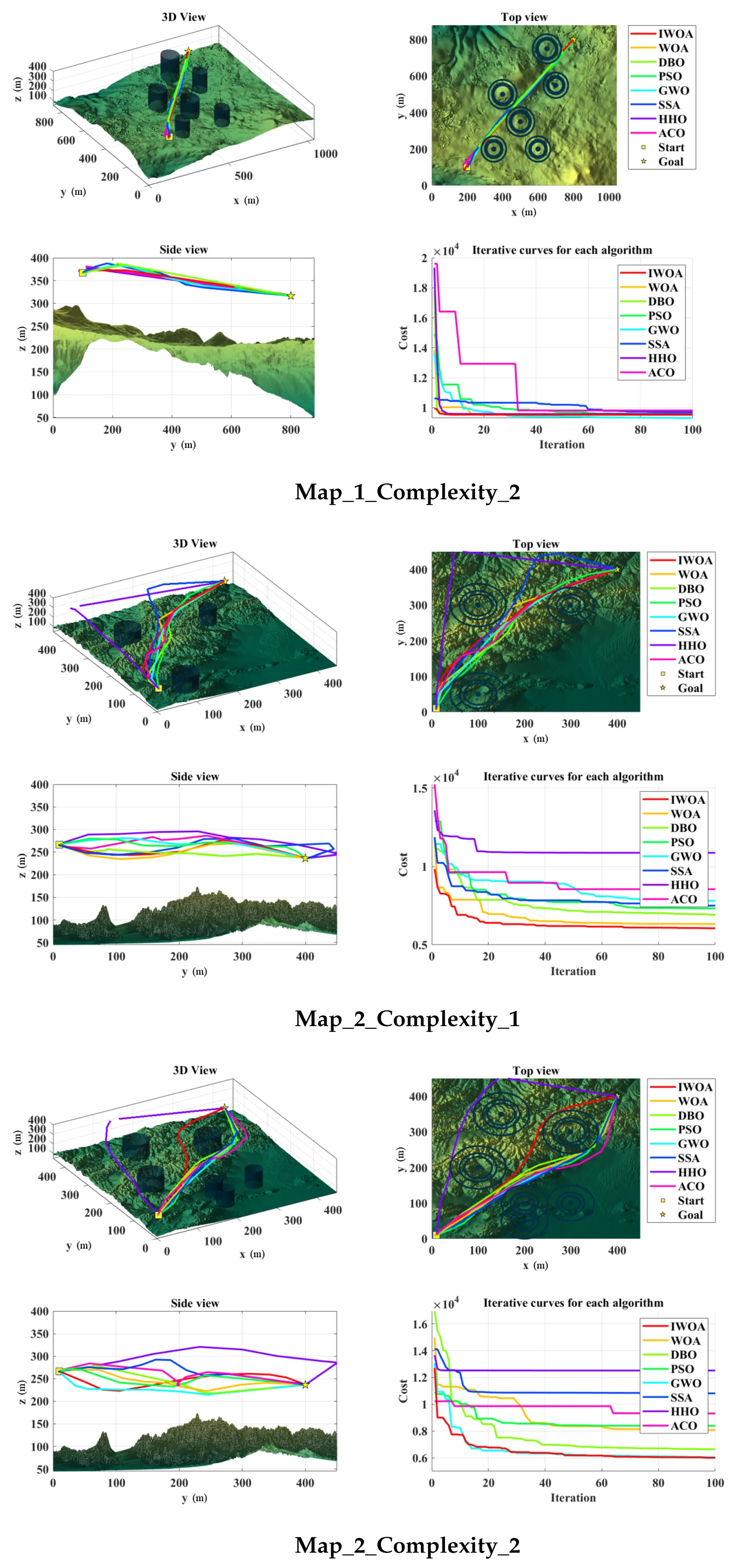

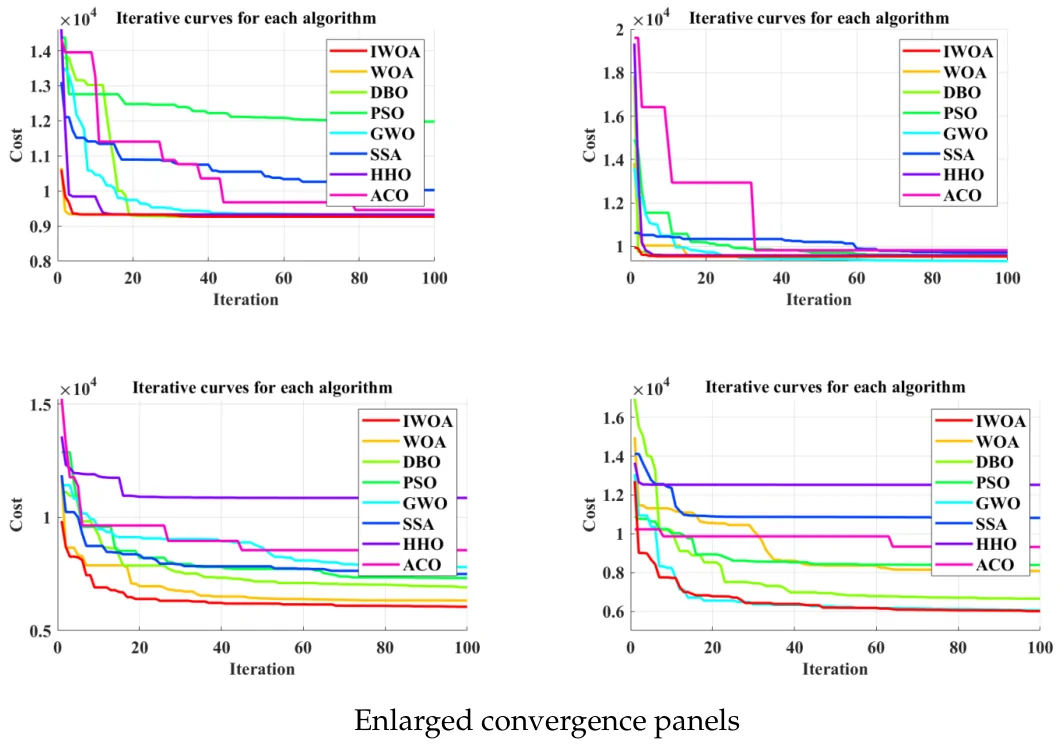
Single-UAV paths and convergence curves for IWOA and seven comparison algorithms. The final plate enlarges the four convergence panels (M1-C1, M1-C2, M2-C1, and M2-C2, read left-to-right and top-to-bottom) extracted from the scenario figures without changing the plotted data.

**Figure 13:**
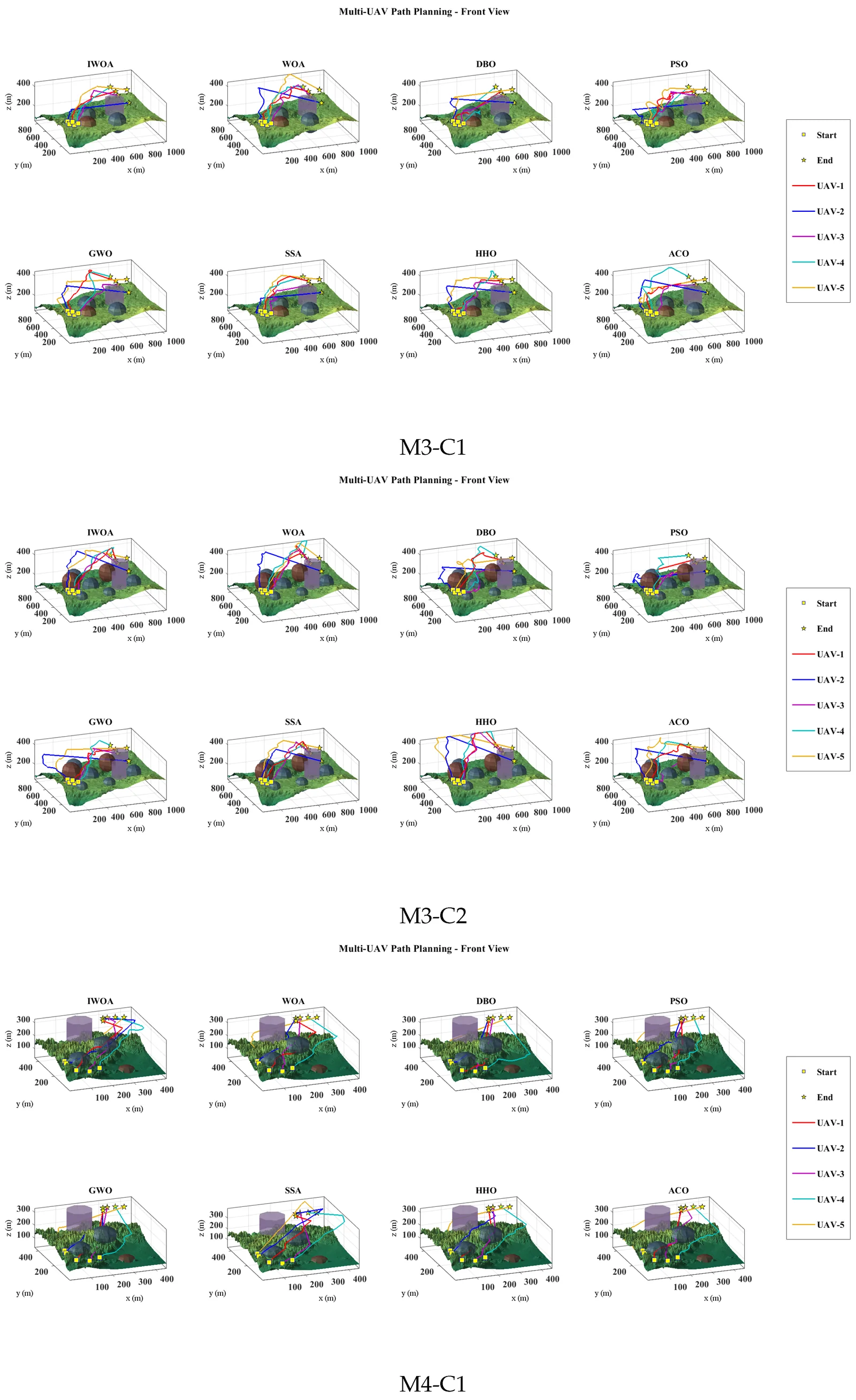

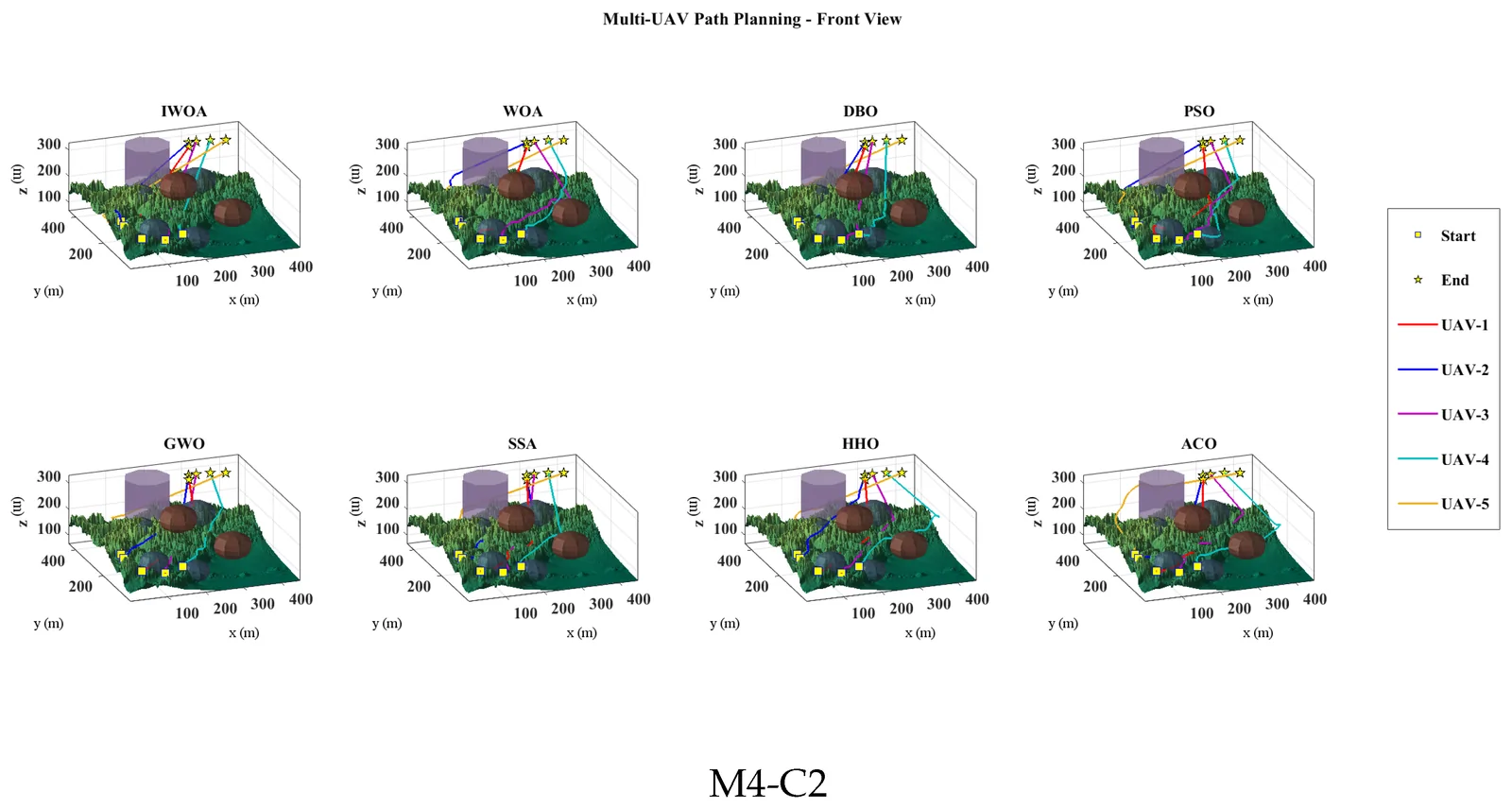
Representative three-dimensional multi-UAV path plates for M3-C1, M3-C2, M4-C1, and M4-C2. Within each plate, colors distinguish UAVs; markers denote starts and goals; translucent objects denote radar threats, other threats, and no-fly zones. These examples are illustrative and are not statistical or feasibility evidence.

**Figure 14:**
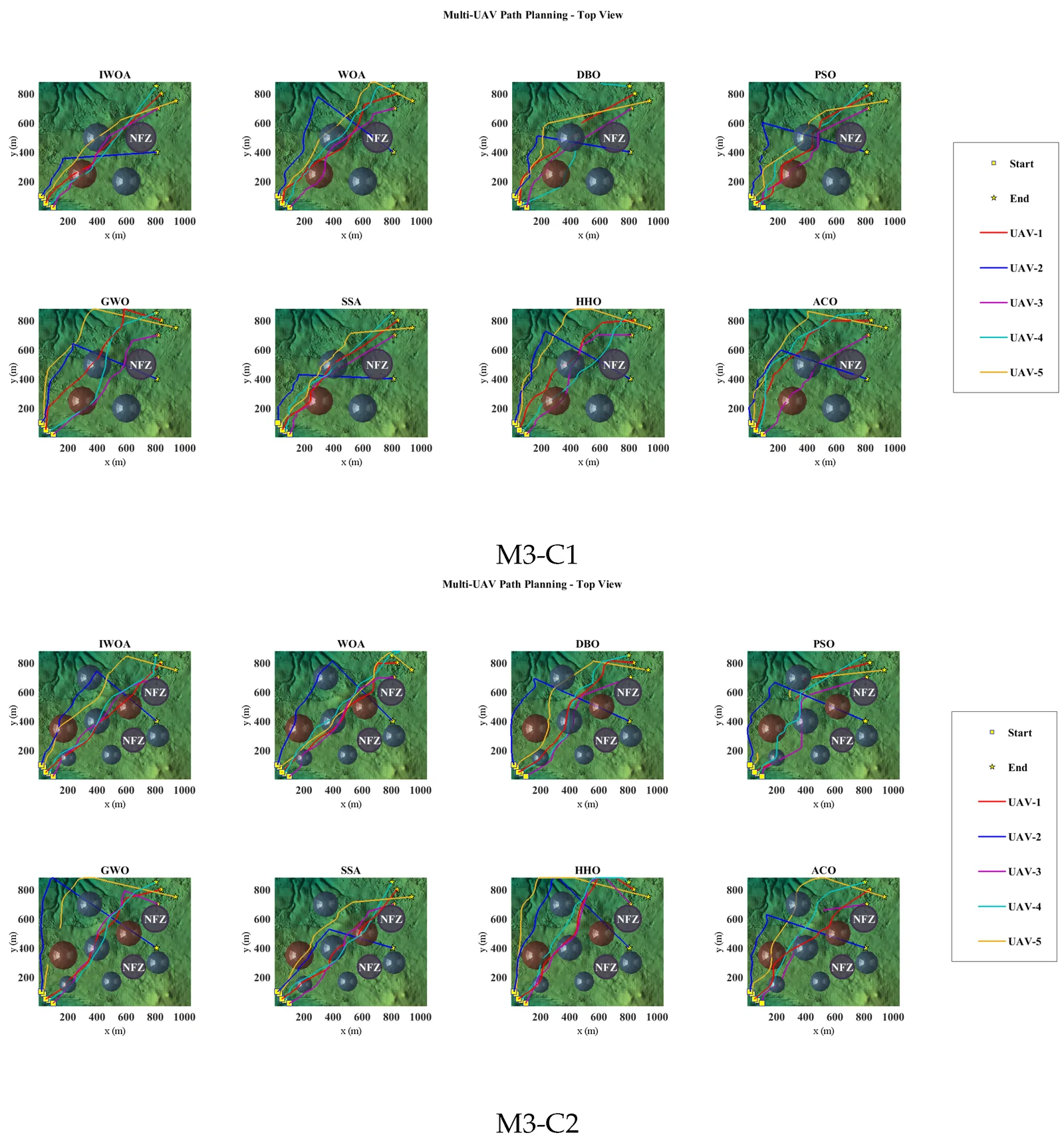

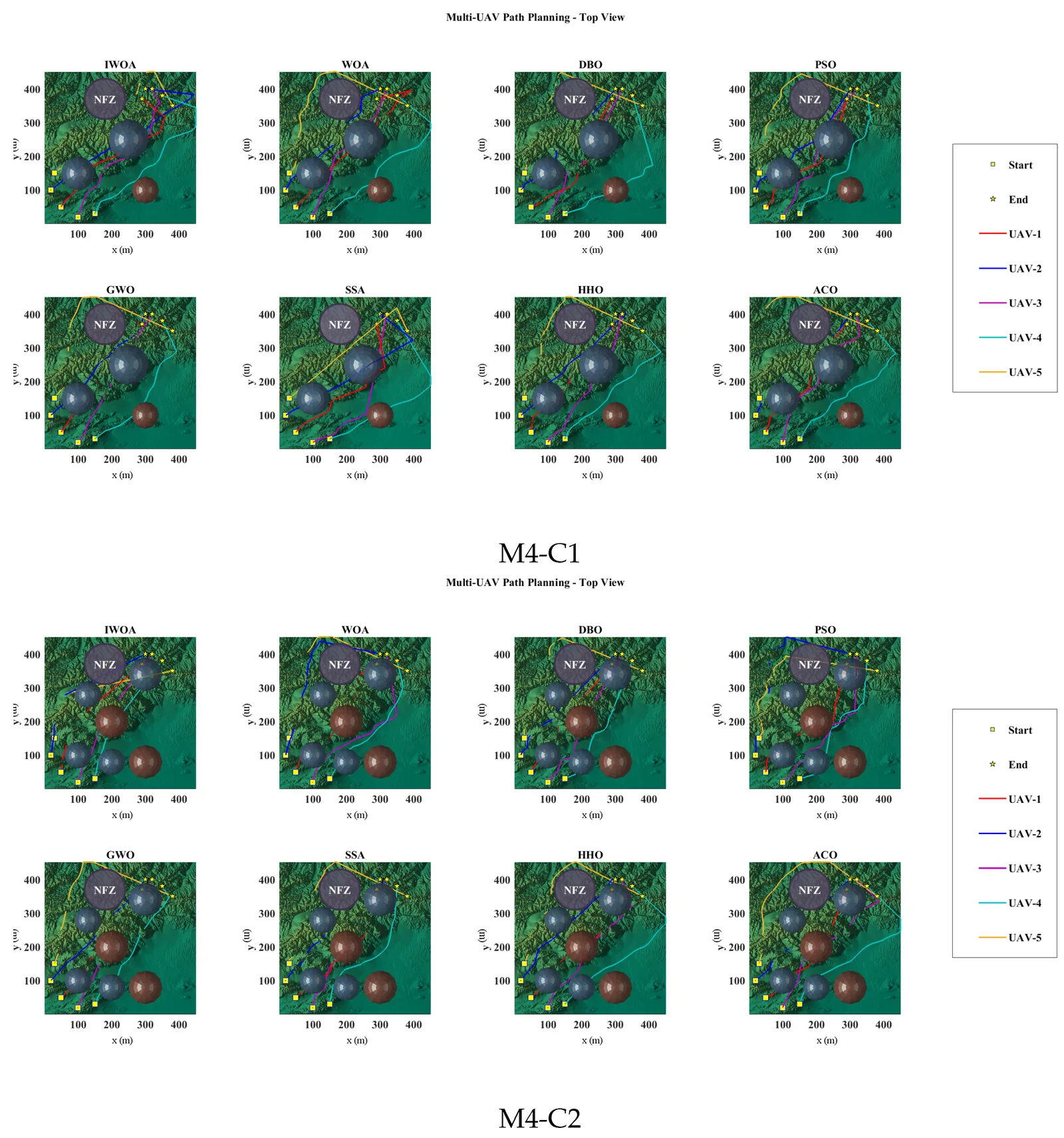
Top views corresponding to Figure 13, ordered M3-C1, M3-C2, M4-C1, and M4-C2. Colors distinguish UAVs and markers denote starts and goals; the plates illustrate geometry only and do not establish time-indexed separation or modeled feasibility.

**Figure 15:**
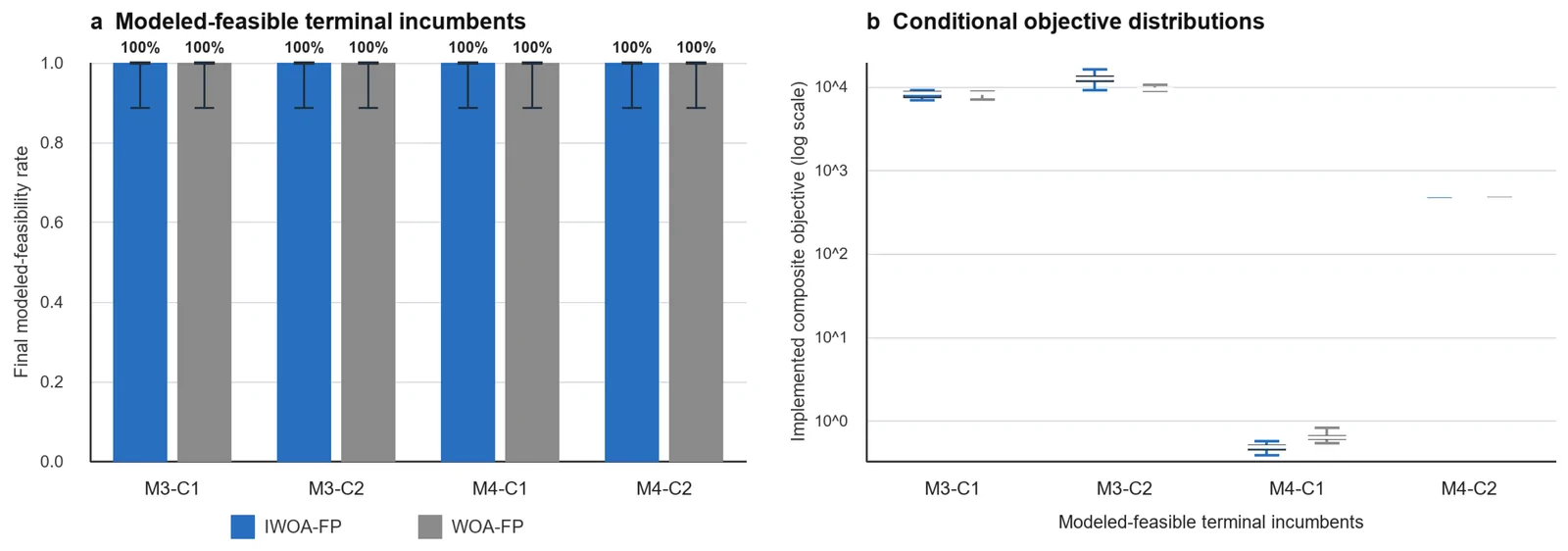
Feasibility-priority validation. (**a**) Final modeled-feasibility proportions for IWOA-FP and WOA-FP; error bars show Wilson 95% intervals. (**b**) Implemented composite-objective distributions conditional on modeled feasibility. Both methods start with the same validated feasible incumbent in each scenario.

**Table 1:** Median [interquartile range] of final composite fitness over 30 independent 500-iteration runs. Lower is better. M4-C1: Map 4, Complexity Setting 1; M4-C2: Map 4, Complexity Setting 2; M3-C2: Map 3, Complexity Setting 2; M3-C1: Map 3, Complexity Setting 1.

Algorithm	M3-C1	M3-C2	M4-C1	M4-C2
IWOA	8763.9 [8444.0, 9920.9]	10,260.9 [9820.5, 10,935.9]	3.539 [2.688, 17.257]	4.047 [3.210, 6.364]
WOA	34,813.3 [33,404.7, 36,667.1]	21,114.3 [19,416.0, 23,110.9]	3.297 [3.104, 3.671]	7.285 [5.121, 15,029.0]
DBO	30,981.5 [11,804.0, 32,616.0]	20,383.1 [17,318.5, 26,563.9]	4.105 [3.943, 4.544]	46.780 [10.015, 11,004.8]
PSO	33,985.0 [14,833.5, 35,081.1]	25,396.0 [19,383.4, 31,217.1]	10.736 [4.543, 21.253]	57.742 [19.217, 379.3]
GWO	36,650.3 [35,443.8, 37,563.0]	30,126.3 [28,335.3, 32,859.9]	4.003 [3.871, 4.546]	5.208 [4.588, 6.163]
SSA	29,507.4 [9674.4, 30,083.8]	10,799.7 [10,698.4, 11,238.9]	2.410 [1.788, 2.894]	4.690 [3.938, 5.354]
HHO	11,097.0 [10,922.2, 11,622.0]	24,442.6 [23,561.0, 25,978.6]	162.3 [52.228, 359.1]	20,008.8 [54.582, 20,027.9]
ACO	9191.9 [9062.7, 9325.9]	13,960.7 [12,918.5, 14,865.7]	2.989 [2.761, 3.328]	4.077 [3.775, 4.293]

**Table 2:** Mean ± standard deviation of final composite fitness and mean Friedman rank over four scenarios.

Algorithm	M3-C1	M3-C2	M4-C1	M4-C2	Rank
IWOA	12,812.8 ± 8361.8	11,259.6 ± 3522.7	63.871 ± 144.2	2688.9 ± 6908.4	2.50
WOA	33,752.6 ± 6043.0	21,383.0 ± 2559.0	12.185 ± 48.397	5348.5 ± 8988.7	5.01
DBO	24,001.2 ± 10,667.0	23,249.8 ± 8381.5	15.943 ± 53.933	6409.1 ± 10,324.0	5.32
PSO	28,953.2 ± 10,112.1	27,269.3 ± 10,401.3	2031.4 ± 6095.8	3083.2 ± 6974.2	6.17
GWO	36,642.1 ± 1789.4	30,758.3 ± 3197.7	4.356 ± 1.097	7.432 ± 7.846	5.88
SSA	21,236.8 ± 10,364.7	11,082.3 ± 822.3	2.359 ± 0.877	1866.4 ± 6606.2	2.65
HHO	14,724.2 ± 8552.4	24,713.2 ± 2029.6	4126.6 ± 8082.3	10,713.9 ± 10,124.4	6.15
ACO	9194.1 ± 270.5	14,334.7 ± 2218.5	3.015 ± 0.452	4.007 ± 0.344	2.33

**Table 3:** Controlled modular WOA-backbone factor screen in M4-C2 over 30 paired 500-iteration runs. Positive rank-biserial values favor the named variant over the modular reference. GPS: Good-Point-Set initialization; AW: Adaptive Weight; PM: Population Migration; CM: Cauchy mutation; All4: integrating all four strategies.

Variant	Mean ± SD	Median [IQR]	Median Change	$p_{\mathrm{Holm}}$	Rank-Biserial
WOA	16,682.5 ± 7465.6	20,068.3 [19,951.0, 20,729.7]	Reference	–	–
WOA + GPS	11,416.9 ± 9686.0	16,016.4 [327.3, 20,482.4]	20.2% lower	0.1406	0.381
WOA + AW	15,099.7 ± 8848.4	20,078.6 [7930.0, 20,495.2]	0.1% higher	0.1406	0.338
WOA + PM	12,256.1 ± 8752.4	17,563.6 [1859.7, 20,047.7]	12.5% lower	0.0093	0.634
WOA + CM	14,767.6 ± 8894.0	20,011.7 [5670.5, 20,022.4]	0.3% lower	0.0017	0.751
WOA + All4	10,570.3 ± 9741.3	13,051.0 [227.3, 20,012.0]	35.0% lower	0.0093	0.639

**Table 4:** Four highlighted IWOA strategies and their intended effects. The production routine also contains legacy pooling, preferential-selection, DFDB, and SOBL mechanisms.

Improvement Strategy	Primary Phase	Issue Addressed	Expected Impact
Good-point-based initialization	Initialization phase	Random population coverage is uneven	Deterministic rank-one initial stratification in the evaluated routine
Nonlinear adaptive weight	Iterative search	Poor exploration-exploitation transition	Regulates guidance by the best individual
Population-migration search	Each iteration	Population aggregation and local stagnation	Moves selected individuals to new regions
Cauchy mutation	Best-solution update	Difficulty escaping local optima	Uses heavy-tailed perturbations to expand the search range

**Table 5:** CEC2017 experimental parameter settings.

Parameters	Official Statistical Experiments	Visualization Experiment
Test functions	F1 and F3–F30	F1, F3, F5, F8, F12, F18, F25, and F28
Problem dimension	30	30
Population size	30	30
Maximum number of iterations	500	1000
Number of independent runs	30	One representative run per function
Function evaluations on the x-axis	–	0–30,000
Primary output	Minimum, mean, standard deviation, median, maximum, and rank	Search history, trajectories, convergence curves, exploration-exploitation ratio, and population diversity

**Table 6:** Performance of eight algorithms on 29 CEC2017 functions.

Function	Statistic	IWOA	WOA	DBO	PSO	GWO	SSA	HHO	ACO
F1	min	$1.04\times 10^{2}$	$2.10\times 10^{9}$	$5.18\times 10^{5}$	$1.70\times 10^{5}$	$2.23\times 10^{8}$	$5.38\times 10^{2}$	$4.27\times 10^{9}$	$1.62\times 10^{8}$
	std	$3.44\times 10^{4}$	$1.73\times 10^{9}$	$1.99\times 10^{8}$	$1.53\times 10^{9}$	$2.09\times 10^{9}$	$6.65\times 10^{3}$	$1.44\times 10^{9}$	$3.87\times 10^{8}$
	avg	$8.45\times 10^{3}$	$5.59\times 10^{9}$	$2.46\times 10^{8}$	$1.19\times 10^{9}$	$2.57\times 10^{9}$	$6.27\times 10^{3}$	$6.83\times 10^{9}$	$7.28\times 10^{8}$
	median	$1.37\times 10^{3}$	$5.64\times 10^{9}$	$1.80\times 10^{8}$	$4.47\times 10^{8}$	$2.01\times 10^{9}$	$3.64\times 10^{3}$	$6.58\times 10^{9}$	$7.07\times 10^{8}$
	worst	$1.90\times 10^{5}$	$9.38\times 10^{9}$	$9.13\times 10^{8}$	$4.47\times 10^{9}$	$8.22\times 10^{9}$	$1.95\times 10^{4}$	$9.65\times 10^{9}$	$1.80\times 10^{9}$
	rank	2	7	3	5	6	1	8	4
F3	min	$1.34\times 10^{3}$	$1.05\times 10^{5}$	$6.67\times 10^{4}$	$2.55\times 10^{4}$	$3.79\times 10^{4}$	$3.71\times 10^{4}$	$6.43\times 10^{4}$	$1.31\times 10^{5}$
	std	$3.01\times 10^{3}$	$7.82\times 10^{4}$	$1.50\times 10^{4}$	$2.25\times 10^{4}$	$1.16\times 10^{4}$	$7.73\times 10^{3}$	$6.86\times 10^{3}$	$3.55\times 10^{4}$
	avg	$5.85\times 10^{3}$	$2.58\times 10^{5}$	$9.01\times 10^{4}$	$6.33\times 10^{4}$	$6.50\times 10^{4}$	$5.04\times 10^{4}$	$8.38\times 10^{4}$	$2.06\times 10^{5}$
	median	$5.35\times 10^{3}$	$2.65\times 10^{5}$	$8.77\times 10^{4}$	$6.17\times 10^{4}$	$6.62\times 10^{4}$	$5.00\times 10^{4}$	$8.56\times 10^{4}$	$2.06\times 10^{5}$
	worst	$1.23\times 10^{4}$	$4.11\times 10^{5}$	$1.39\times 10^{5}$	$1.20\times 10^{5}$	$9.17\times 10^{4}$	$6.60\times 10^{4}$	$9.17\times 10^{4}$	$2.82\times 10^{5}$
	rank	1	8	6	3	4	2	5	7
F4	min	$4.63\times 10^{2}$	$8.46\times 10^{2}$	$5.23\times 10^{2}$	$4.49\times 10^{2}$	$5.12\times 10^{2}$	$4.72\times 10^{2}$	$8.38\times 10^{2}$	$5.06\times 10^{2}$
	std	$1.82\times 10^{1}$	$4.62\times 10^{2}$	$1.24\times 10^{2}$	$2.92\times 10^{2}$	$1.33\times 10^{2}$	$1.65\times 10^{1}$	$5.23\times 10^{2}$	$2.15\times 10^{1}$
	avg	$5.05\times 10^{2}$	$1.39\times 10^{3}$	$6.41\times 10^{2}$	$6.54\times 10^{2}$	$6.28\times 10^{2}$	$5.01\times 10^{2}$	$1.70\times 10^{3}$	$5.24\times 10^{2}$
	median	$5.13\times 10^{2}$	$1.21\times 10^{3}$	$5.94\times 10^{2}$	$5.47\times 10^{2}$	$5.87\times 10^{2}$	$5.05\times 10^{2}$	$1.72\times 10^{3}$	$5.19\times 10^{2}$
	worst	$5.31\times 10^{2}$	$2.57\times 10^{3}$	$1.15\times 10^{3}$	$1.91\times 10^{3}$	$1.16\times 10^{3}$	$5.32\times 10^{2}$	$3.12\times 10^{3}$	$6.07\times 10^{2}$
	rank	2	7	5	6	4	1	8	3
F5	min	$5.41\times 10^{2}$	$7.72\times 10^{2}$	$6.34\times 10^{2}$	$5.67\times 10^{2}$	$5.77\times 10^{2}$	$6.24\times 10^{2}$	$7.07\times 10^{2}$	$7.34\times 10^{2}$
	std	$3.18\times 10^{1}$	$5.53\times 10^{1}$	$5.70\times 10^{1}$	$2.97\times 10^{1}$	$4.53\times 10^{1}$	$4.98\times 10^{1}$	$4.91\times 10^{1}$	$1.51\times 10^{1}$
	avg	$5.86\times 10^{2}$	$8.82\times 10^{2}$	$7.62\times 10^{2}$	$6.23\times 10^{2}$	$6.33\times 10^{2}$	$7.49\times 10^{2}$	$7.99\times 10^{2}$	$7.60\times 10^{2}$
	median	$5.86\times 10^{2}$	$8.71\times 10^{2}$	$7.74\times 10^{2}$	$6.21\times 10^{2}$	$6.18\times 10^{2}$	$7.44\times 10^{2}$	$7.90\times 10^{2}$	$7.59\times 10^{2}$
	worst	$6.56\times 10^{2}$	$9.75\times 10^{2}$	$8.42\times 10^{2}$	$6.71\times 10^{2}$	$7.56\times 10^{2}$	$8.21\times 10^{2}$	$8.76\times 10^{2}$	$7.87\times 10^{2}$
	rank	1	8	6	2	3	4	7	5
F6	min	$6.00\times 10^{2}$	$6.60\times 10^{2}$	$6.23\times 10^{2}$	$6.03\times 10^{2}$	$6.05\times 10^{2}$	$6.31\times 10^{2}$	$6.51\times 10^{2}$	$6.10\times 10^{2}$
	std	$6.00\times 10^{0}$	$1.14\times 10^{1}$	$1.18\times 10^{1}$	$1.27\times 10^{1}$	$5.04\times 10^{0}$	$1.20\times 10^{1}$	$7.34\times 10^{0}$	$3.27\times 10^{0}$
	avg	$6.04\times 10^{2}$	$6.80\times 10^{2}$	$6.47\times 10^{2}$	$6.16\times 10^{2}$	$6.12\times 10^{2}$	$6.48\times 10^{2}$	$6.68\times 10^{2}$	$6.15\times 10^{2}$
	median	$6.01\times 10^{2}$	$6.79\times 10^{2}$	$6.47\times 10^{2}$	$6.11\times 10^{2}$	$6.11\times 10^{2}$	$6.45\times 10^{2}$	$6.69\times 10^{2}$	$6.15\times 10^{2}$
	worst	$6.22\times 10^{2}$	$7.06\times 10^{2}$	$6.81\times 10^{2}$	$6.62\times 10^{2}$	$6.24\times 10^{2}$	$6.83\times 10^{2}$	$6.82\times 10^{2}$	$6.23\times 10^{2}$
	rank	1	8	5	4	2	6	7	3
F7	min	$7.66\times 10^{2}$	$1.18\times 10^{3}$	$8.55\times 10^{2}$	$8.26\times 10^{2}$	$8.15\times 10^{2}$	$9.83\times 10^{2}$	$1.10\times 10^{3}$	$1.01\times 10^{3}$
	std	$2.38\times 10^{1}$	$7.89\times 10^{1}$	$8.43\times 10^{1}$	$4.96\times 10^{1}$	$6.46\times 10^{1}$	$9.90\times 10^{1}$	$5.62\times 10^{1}$	$3.02\times 10^{1}$
	avg	$8.03\times 10^{2}$	$1.33\times 10^{3}$	$1.04\times 10^{3}$	$9.16\times 10^{2}$	$9.13\times 10^{2}$	$1.22\times 10^{3}$	$1.26\times 10^{3}$	$1.05\times 10^{3}$
	median	$8.04\times 10^{2}$	$1.33\times 10^{3}$	$1.04\times 10^{3}$	$9.15\times 10^{2}$	$8.99\times 10^{2}$	$1.23\times 10^{3}$	$1.27\times 10^{3}$	$1.05\times 10^{3}$
	worst	$8.83\times 10^{2}$	$1.48\times 10^{3}$	$1.24\times 10^{3}$	$1.01\times 10^{3}$	$1.07\times 10^{3}$	$1.34\times 10^{3}$	$1.38\times 10^{3}$	$1.13\times 10^{3}$
	rank	1	8	4	3	2	6	7	5
F8	min	$8.48\times 10^{2}$	$9.70\times 10^{2}$	$9.06\times 10^{2}$	$8.64\times 10^{2}$	$8.56\times 10^{2}$	$9.14\times 10^{2}$	$9.68\times 10^{2}$	$1.03\times 10^{3}$
	std	$2.64\times 10^{1}$	$5.20\times 10^{1}$	$5.60\times 10^{1}$	$2.46\times 10^{1}$	$4.06\times 10^{1}$	$2.98\times 10^{1}$	$2.64\times 10^{1}$	$1.56\times 10^{1}$
	avg	$8.88\times 10^{2}$	$1.07\times 10^{3}$	$1.03\times 10^{3}$	$9.10\times 10^{2}$	$9.11\times 10^{2}$	$9.81\times 10^{2}$	$1.01\times 10^{3}$	$1.06\times 10^{3}$
	median	$8.83\times 10^{2}$	$1.06\times 10^{3}$	$1.04\times 10^{3}$	$9.09\times 10^{2}$	$8.98\times 10^{2}$	$9.84\times 10^{2}$	$1.01\times 10^{3}$	$1.07\times 10^{3}$
	worst	$9.64\times 10^{2}$	$1.18\times 10^{3}$	$1.11\times 10^{3}$	$9.65\times 10^{2}$	$1.04\times 10^{3}$	$1.03\times 10^{3}$	$1.08\times 10^{3}$	$1.09\times 10^{3}$
	rank	1	8	6	2	3	4	5	7
F9	min	$9.06\times 10^{2}$	$6.15\times 10^{3}$	$2.48\times 10^{3}$	$9.53\times 10^{2}$	$1.45\times 10^{3}$	$3.92\times 10^{3}$	$4.57\times 10^{3}$	$1.77\times 10^{3}$
	std	$5.62\times 10^{2}$	$4.75\times 10^{3}$	$2.14\times 10^{3}$	$3.47\times 10^{3}$	$8.39\times 10^{2}$	$3.97\times 10^{2}$	$1.45\times 10^{3}$	$9.28\times 10^{2}$
	avg	$1.32\times 10^{3}$	$1.12\times 10^{4}$	$6.41\times 10^{3}$	$3.34\times 10^{3}$	$2.46\times 10^{3}$	$5.31\times 10^{3}$	$7.04\times 10^{3}$	$3.37\times 10^{3}$
	median	$1.10\times 10^{3}$	$9.82\times 10^{3}$	$6.35\times 10^{3}$	$2.12\times 10^{3}$	$2.29\times 10^{3}$	$5.39\times 10^{3}$	$6.85\times 10^{3}$	$3.41\times 10^{3}$
	worst	$3.65\times 10^{3}$	$2.27\times 10^{4}$	$1.06\times 10^{4}$	$1.81\times 10^{4}$	$4.69\times 10^{3}$	$5.95\times 10^{3}$	$1.12\times 10^{4}$	$6.08\times 10^{3}$
	rank	1	8	6	3	2	5	7	4
F10	min	$3.96\times 10^{3}$	$6.21\times 10^{3}$	$4.82\times 10^{3}$	$3.48\times 10^{3}$	$2.46\times 10^{3}$	$4.14\times 10^{3}$	$5.48\times 10^{3}$	$8.22\times 10^{3}$
	std	$7.33\times 10^{2}$	$7.18\times 10^{2}$	$1.23\times 10^{3}$	$7.10\times 10^{2}$	$1.44\times 10^{3}$	$6.79\times 10^{2}$	$7.42\times 10^{2}$	$3.26\times 10^{2}$
	avg	$5.38\times 10^{3}$	$7.50\times 10^{3}$	$6.68\times 10^{3}$	$4.80\times 10^{3}$	$5.09\times 10^{3}$	$5.43\times 10^{3}$	$7.32\times 10^{3}$	$8.76\times 10^{3}$
	median	$5.32\times 10^{3}$	$7.60\times 10^{3}$	$6.57\times 10^{3}$	$4.67\times 10^{3}$	$4.76\times 10^{3}$	$5.38\times 10^{3}$	$7.43\times 10^{3}$	$8.80\times 10^{3}$
	worst	$7.24\times 10^{3}$	$9.20\times 10^{3}$	$8.96\times 10^{3}$	$6.30\times 10^{3}$	$9.22\times 10^{3}$	$7.10\times 10^{3}$	$9.15\times 10^{3}$	$9.39\times 10^{3}$
	rank	3	7	5	1	2	4	6	8
F11	min	$1.13\times 10^{3}$	$2.55\times 10^{3}$	$1.40\times 10^{3}$	$1.17\times 10^{3}$	$1.36\times 10^{3}$	$1.18\times 10^{3}$	$3.17\times 10^{3}$	$1.39\times 10^{3}$
	std	$4.00\times 10^{1}$	$3.38\times 10^{3}$	$9.33\times 10^{2}$	$6.40\times 10^{1}$	$9.82\times 10^{2}$	$7.75\times 10^{1}$	$9.72\times 10^{2}$	$5.06\times 10^{2}$
	avg	$1.20\times 10^{3}$	$9.33\times 10^{3}$	$2.08\times 10^{3}$	$1.26\times 10^{3}$	$2.20\times 10^{3}$	$1.30\times 10^{3}$	$5.18\times 10^{3}$	$1.97\times 10^{3}$
	median	$1.21\times 10^{3}$	$8.67\times 10^{3}$	$1.81\times 10^{3}$	$1.24\times 10^{3}$	$1.84\times 10^{3}$	$1.28\times 10^{3}$	$5.20\times 10^{3}$	$1.79\times 10^{3}$
	worst	$1.30\times 10^{3}$	$1.79\times 10^{4}$	$6.26\times 10^{3}$	$1.40\times 10^{3}$	$5.21\times 10^{3}$	$1.51\times 10^{3}$	$6.66\times 10^{3}$	$3.83\times 10^{3}$
	rank	1	8	5	2	6	3	7	4
F12	min	$2.11\times 10^{5}$	$8.35\times 10^{7}$	$2.42\times 10^{6}$	$3.97\times 10^{5}$	$1.43\times 10^{7}$	$1.66\times 10^{5}$	$1.43\times 10^{8}$	$1.65\times 10^{6}$
	std	$1.30\times 10^{6}$	$3.22\times 10^{8}$	$8.56\times 10^{7}$	$1.66\times 10^{8}$	$1.32\times 10^{8}$	$1.15\times 10^{6}$	$5.31\times 10^{8}$	$9.54\times 10^{6}$
	avg	$1.92\times 10^{6}$	$5.20\times 10^{8}$	$4.81\times 10^{7}$	$6.54\times 10^{7}$	$1.20\times 10^{8}$	$1.36\times 10^{6}$	$8.37\times 10^{8}$	$1.52\times 10^{7}$
	median	$1.65\times 10^{6}$	$4.59\times 10^{8}$	$2.22\times 10^{7}$	$2.24\times 10^{6}$	$7.05\times 10^{7}$	$8.49\times 10^{5}$	$7.53\times 10^{8}$	$1.40\times 10^{7}$
	worst	$4.68\times 10^{6}$	$1.59\times 10^{9}$	$4.13\times 10^{8}$	$6.30\times 10^{8}$	$6.44\times 10^{8}$	$4.41\times 10^{6}$	$2.00\times 10^{9}$	$4.24\times 10^{7}$
	rank	2	7	4	5	6	1	8	3
F13	min	$1.65\times 10^{3}$	$1.27\times 10^{6}$	$4.38\times 10^{4}$	$1.62\times 10^{3}$	$8.50\times 10^{4}$	$3.94\times 10^{3}$	$8.23\times 10^{5}$	$1.69\times 10^{3}$
	std	$1.54\times 10^{4}$	$9.18\times 10^{6}$	$9.93\times 10^{6}$	$1.31\times 10^{7}$	$3.56\times 10^{6}$	$2.10\times 10^{4}$	$2.66\times 10^{7}$	$2.06\times 10^{4}$
	avg	$1.38\times 10^{4}$	$1.12\times 10^{7}$	$5.37\times 10^{6}$	$2.42\times 10^{6}$	$1.22\times 10^{6}$	$3.06\times 10^{4}$	$1.53\times 10^{7}$	$1.98\times 10^{4}$
	median	$8.78\times 10^{3}$	$7.70\times 10^{6}$	$1.20\times 10^{6}$	$2.03\times 10^{4}$	$1.81\times 10^{5}$	$2.08\times 10^{4}$	$5.21\times 10^{6}$	$7.82\times 10^{3}$
	worst	$6.27\times 10^{4}$	$3.86\times 10^{7}$	$4.58\times 10^{7}$	$7.17\times 10^{7}$	$1.71\times 10^{7}$	$6.56\times 10^{4}$	$1.40\times 10^{8}$	$6.51\times 10^{4}$
	rank	1	7	6	5	4	3	8	2
F14	min	$1.48\times 10^{3}$	$1.86\times 10^{4}$	$8.14\times 10^{3}$	$2.18\times 10^{3}$	$8.76\times 10^{3}$	$6.20\times 10^{3}$	$7.34\times 10^{4}$	$1.62\times 10^{4}$
	std	$4.04\times 10^{2}$	$3.20\times 10^{6}$	$6.91\times 10^{5}$	$7.72\times 10^{4}$	$6.61\times 10^{5}$	$3.56\times 10^{4}$	$1.02\times 10^{6}$	$2.58\times 10^{5}$
	avg	$1.77\times 10^{3}$	$2.74\times 10^{6}$	$4.96\times 10^{5}$	$7.83\times 10^{4}$	$5.95\times 10^{5}$	$5.69\times 10^{4}$	$1.35\times 10^{6}$	$2.46\times 10^{5}$
	median	$1.61\times 10^{3}$	$1.47\times 10^{6}$	$2.27\times 10^{5}$	$4.79\times 10^{4}$	$3.54\times 10^{5}$	$4.81\times 10^{4}$	$1.19\times 10^{6}$	$1.84\times 10^{5}$
	worst	$3.26\times 10^{3}$	$1.33\times 10^{7}$	$2.46\times 10^{6}$	$2.93\times 10^{5}$	$2.25\times 10^{6}$	$1.54\times 10^{5}$	$3.78\times 10^{6}$	$1.29\times 10^{6}$
	rank	1	8	5	3	6	2	7	4
F15	min	$1.73\times 10^{3}$	$1.64\times 10^{5}$	$1.13\times 10^{4}$	$1.90\times 10^{3}$	$2.73\times 10^{4}$	$2.15\times 10^{3}$	$1.61\times 10^{4}$	$1.76\times 10^{3}$
	std	$8.45\times 10^{3}$	$9.38\times 10^{6}$	$3.24\times 10^{5}$	$1.19\times 10^{4}$	$5.01\times 10^{6}$	$1.67\times 10^{4}$	$7.27\times 10^{5}$	$1.35\times 10^{4}$
	avg	$7.97\times 10^{3}$	$7.52\times 10^{6}$	$1.52\times 10^{5}$	$1.02\times 10^{4}$	$1.83\times 10^{6}$	$1.57\times 10^{4}$	$4.29\times 10^{5}$	$2.17\times 10^{4}$
	median	$5.07\times 10^{3}$	$3.09\times 10^{6}$	$7.51\times 10^{4}$	$5.32\times 10^{3}$	$9.04\times 10^{4}$	$9.99\times 10^{3}$	$1.98\times 10^{5}$	$2.29\times 10^{4}$
	worst	$4.13\times 10^{4}$	$3.88\times 10^{7}$	$1.80\times 10^{6}$	$4.68\times 10^{4}$	$2.58\times 10^{7}$	$7.16\times 10^{4}$	$3.73\times 10^{6}$	$4.27\times 10^{4}$
	rank	1	8	5	2	7	3	6	4
F16	min	$1.92\times 10^{3}$	$3.16\times 10^{3}$	$2.42\times 10^{3}$	$2.16\times 10^{3}$	$2.01\times 10^{3}$	$2.28\times 10^{3}$	$3.18\times 10^{3}$	$3.04\times 10^{3}$
	std	$3.02\times 10^{2}$	$9.34\times 10^{2}$	$4.00\times 10^{2}$	$2.24\times 10^{2}$	$3.45\times 10^{2}$	$3.69\times 10^{2}$	$5.57\times 10^{2}$	$1.94\times 10^{2}$
	avg	$2.57\times 10^{3}$	$4.46\times 10^{3}$	$3.41\times 10^{3}$	$2.56\times 10^{3}$	$2.62\times 10^{3}$	$2.94\times 10^{3}$	$4.10\times 10^{3}$	$3.44\times 10^{3}$
	median	$2.55\times 10^{3}$	$4.33\times 10^{3}$	$3.35\times 10^{3}$	$2.56\times 10^{3}$	$2.60\times 10^{3}$	$2.98\times 10^{3}$	$4.04\times 10^{3}$	$3.47\times 10^{3}$
	worst	$3.17\times 10^{3}$	$7.69\times 10^{3}$	$4.12\times 10^{3}$	$3.03\times 10^{3}$	$3.66\times 10^{3}$	$3.60\times 10^{3}$	$5.83\times 10^{3}$	$3.77\times 10^{3}$
	rank	2	8	5	1	3	4	7	6
F17	min	$1.78\times 10^{3}$	$2.18\times 10^{3}$	$2.41\times 10^{3}$	$1.79\times 10^{3}$	$1.86\times 10^{3}$	$2.00\times 10^{3}$	$2.22\times 10^{3}$	$2.19\times 10^{3}$
	std	$1.95\times 10^{2}$	$2.87\times 10^{2}$	$2.08\times 10^{2}$	$2.29\times 10^{2}$	$1.87\times 10^{2}$	$2.84\times 10^{2}$	$2.73\times 10^{2}$	$1.65\times 10^{2}$
	avg	$2.08\times 10^{3}$	$2.70\times 10^{3}$	$2.75\times 10^{3}$	$2.20\times 10^{3}$	$2.12\times 10^{3}$	$2.47\times 10^{3}$	$2.69\times 10^{3}$	$2.53\times 10^{3}$
	median	$2.06\times 10^{3}$	$2.70\times 10^{3}$	$2.75\times 10^{3}$	$2.20\times 10^{3}$	$2.08\times 10^{3}$	$2.41\times 10^{3}$	$2.66\times 10^{3}$	$2.55\times 10^{3}$
	worst	$2.46\times 10^{3}$	$3.31\times 10^{3}$	$3.15\times 10^{3}$	$2.60\times 10^{3}$	$2.56\times 10^{3}$	$3.22\times 10^{3}$	$3.36\times 10^{3}$	$2.81\times 10^{3}$
	rank	1	7	8	3	2	4	6	5
F18	min	$5.13\times 10^{3}$	$6.39\times 10^{5}$	$1.19\times 10^{5}$	$1.46\times 10^{5}$	$1.84\times 10^{5}$	$3.78\times 10^{4}$	$1.04\times 10^{5}$	$9.72\times 10^{5}$
	std	$1.67\times 10^{5}$	$2.65\times 10^{7}$	$1.14\times 10^{7}$	$1.45\times 10^{6}$	$4.74\times 10^{6}$	$6.51\times 10^{5}$	$1.02\times 10^{7}$	$7.50\times 10^{6}$
	avg	$9.11\times 10^{4}$	$2.14\times 10^{7}$	$5.34\times 10^{6}$	$1.56\times 10^{6}$	$2.48\times 10^{6}$	$5.50\times 10^{5}$	$9.48\times 10^{6}$	$1.19\times 10^{7}$
	median	$4.28\times 10^{4}$	$8.18\times 10^{6}$	$2.28\times 10^{6}$	$9.15\times 10^{5}$	$1.35\times 10^{6}$	$3.68\times 10^{5}$	$6.35\times 10^{6}$	$1.19\times 10^{7}$
	worst	$9.09\times 10^{5}$	$9.93\times 10^{7}$	$6.18\times 10^{7}$	$5.55\times 10^{6}$	$2.65\times 10^{7}$	$3.20\times 10^{6}$	$4.06\times 10^{7}$	$2.93\times 10^{7}$
	rank	1	8	5	3	4	2	6	7
F19	min	$2.10\times 10^{3}$	$3.55\times 10^{5}$	$2.08\times 10^{3}$	$2.11\times 10^{3}$	$6.42\times 10^{4}$	$2.08\times 10^{3}$	$2.84\times 10^{5}$	$2.41\times 10^{3}$
	std	$4.74\times 10^{3}$	$2.16\times 10^{7}$	$4.86\times 10^{6}$	$9.11\times 10^{4}$	$1.22\times 10^{6}$	$1.66\times 10^{4}$	$1.31\times 10^{7}$	$2.03\times 10^{4}$
	avg	$7.12\times 10^{3}$	$1.87\times 10^{7}$	$2.54\times 10^{6}$	$3.52\times 10^{4}$	$1.01\times 10^{6}$	$1.15\times 10^{4}$	$1.52\times 10^{7}$	$2.38\times 10^{4}$
	median	$5.40\times 10^{3}$	$1.20\times 10^{7}$	$4.78\times 10^{5}$	$6.35\times 10^{3}$	$7.15\times 10^{5}$	$4.37\times 10^{3}$	$1.07\times 10^{7}$	$1.67\times 10^{4}$
	worst	$2.17\times 10^{4}$	$9.17\times 10^{7}$	$2.15\times 10^{7}$	$3.79\times 10^{5}$	$5.88\times 10^{6}$	$5.66\times 10^{4}$	$4.04\times 10^{7}$	$5.65\times 10^{4}$
	rank	1	8	6	4	5	2	7	3
F20	min	$2.13\times 10^{3}$	$2.47\times 10^{3}$	$2.38\times 10^{3}$	$2.22\times 10^{3}$	$2.21\times 10^{3}$	$2.36\times 10^{3}$	$2.36\times 10^{3}$	$2.15\times 10^{3}$
	std	$1.94\times 10^{2}$	$2.10\times 10^{2}$	$1.78\times 10^{2}$	$2.02\times 10^{2}$	$1.48\times 10^{2}$	$1.94\times 10^{2}$	$2.04\times 10^{2}$	$1.89\times 10^{2}$
	avg	$2.54\times 10^{3}$	$2.93\times 10^{3}$	$2.70\times 10^{3}$	$2.51\times 10^{3}$	$2.43\times 10^{3}$	$2.77\times 10^{3}$	$2.80\times 10^{3}$	$2.75\times 10^{3}$
	median	$2.48\times 10^{3}$	$2.99\times 10^{3}$	$2.71\times 10^{3}$	$2.49\times 10^{3}$	$2.37\times 10^{3}$	$2.79\times 10^{3}$	$2.79\times 10^{3}$	$2.79\times 10^{3}$
	worst	$2.99\times 10^{3}$	$3.28\times 10^{3}$	$3.07\times 10^{3}$	$3.07\times 10^{3}$	$2.76\times 10^{3}$	$3.10\times 10^{3}$	$3.13\times 10^{3}$	$3.05\times 10^{3}$
	rank	3	8	4	2	1	6	7	5
F21	min	$2.32\times 10^{3}$	$2.52\times 10^{3}$	$2.44\times 10^{3}$	$2.36\times 10^{3}$	$2.36\times 10^{3}$	$2.43\times 10^{3}$	$2.51\times 10^{3}$	$2.50\times 10^{3}$
	std	$1.76\times 10^{1}$	$8.05\times 10^{1}$	$4.86\times 10^{1}$	$2.56\times 10^{1}$	$4.89\times 10^{1}$	$4.12\times 10^{1}$	$4.58\times 10^{1}$	$1.71\times 10^{1}$
	avg	$2.36\times 10^{3}$	$2.64\times 10^{3}$	$2.55\times 10^{3}$	$2.42\times 10^{3}$	$2.42\times 10^{3}$	$2.50\times 10^{3}$	$2.58\times 10^{3}$	$2.54\times 10^{3}$
	median	$2.36\times 10^{3}$	$2.63\times 10^{3}$	$2.56\times 10^{3}$	$2.42\times 10^{3}$	$2.40\times 10^{3}$	$2.50\times 10^{3}$	$2.57\times 10^{3}$	$2.54\times 10^{3}$
	worst	$2.39\times 10^{3}$	$2.85\times 10^{3}$	$2.67\times 10^{3}$	$2.46\times 10^{3}$	$2.53\times 10^{3}$	$2.59\times 10^{3}$	$2.70\times 10^{3}$	$2.58\times 10^{3}$
	rank	1	8	6	3	2	4	7	5
F22	min	$2.30\times 10^{3}$	$5.03\times 10^{3}$	$2.36\times 10^{3}$	$2.31\times 10^{3}$	$2.49\times 10^{3}$	$2.30\times 10^{3}$	$3.16\times 10^{3}$	$2.52\times 10^{3}$
	std	$1.26\times 10^{3}$	$1.14\times 10^{3}$	$2.67\times 10^{3}$	$1.92\times 10^{3}$	$2.13\times 10^{3}$	$1.63\times 10^{3}$	$2.01\times 10^{3}$	$2.57\times 10^{3}$
	avg	$2.71\times 10^{3}$	$8.65\times 10^{3}$	$4.79\times 10^{3}$	$4.48\times 10^{3}$	$4.73\times 10^{3}$	$6.70\times 10^{3}$	$6.92\times 10^{3}$	$8.98\times 10^{3}$
	median	$2.30\times 10^{3}$	$8.70\times 10^{3}$	$2.51\times 10^{3}$	$3.97\times 10^{3}$	$4.63\times 10^{3}$	$7.21\times 10^{3}$	$7.54\times 10^{3}$	$9.84\times 10^{3}$
	worst	$7.04\times 10^{3}$	$1.08\times 10^{4}$	$9.75\times 10^{3}$	$7.59\times 10^{3}$	$9.77\times 10^{3}$	$8.39\times 10^{3}$	$9.58\times 10^{3}$	$1.06\times 10^{4}$
	rank	1	7	4	2	3	5	6	8
F23	min	$2.69\times 10^{3}$	$2.92\times 10^{3}$	$2.91\times 10^{3}$	$2.78\times 10^{3}$	$2.72\times 10^{3}$	$2.80\times 10^{3}$	$3.01\times 10^{3}$	$2.86\times 10^{3}$
	std	$2.23\times 10^{1}$	$1.13\times 10^{2}$	$7.38\times 10^{1}$	$9.75\times 10^{1}$	$4.45\times 10^{1}$	$6.53\times 10^{1}$	$9.89\times 10^{1}$	$1.38\times 10^{1}$
	avg	$2.74\times 10^{3}$	$3.17\times 10^{3}$	$3.02\times 10^{3}$	$2.92\times 10^{3}$	$2.78\times 10^{3}$	$2.91\times 10^{3}$	$3.22\times 10^{3}$	$2.89\times 10^{3}$
	median	$2.73\times 10^{3}$	$3.15\times 10^{3}$	$3.01\times 10^{3}$	$2.88\times 10^{3}$	$2.77\times 10^{3}$	$2.91\times 10^{3}$	$3.23\times 10^{3}$	$2.89\times 10^{3}$
	worst	$2.77\times 10^{3}$	$3.51\times 10^{3}$	$3.17\times 10^{3}$	$3.15\times 10^{3}$	$2.91\times 10^{3}$	$3.05\times 10^{3}$	$3.47\times 10^{3}$	$2.91\times 10^{3}$
	rank	1	7	6	5	2	4	8	3
F24	min	$2.85\times 10^{3}$	$3.06\times 10^{3}$	$3.03\times 10^{3}$	$2.97\times 10^{3}$	$2.88\times 10^{3}$	$2.92\times 10^{3}$	$3.19\times 10^{3}$	$3.02\times 10^{3}$
	std	$2.86\times 10^{1}$	$9.72\times 10^{1}$	$7.78\times 10^{1}$	$5.75\times 10^{1}$	$5.41\times 10^{1}$	$7.90\times 10^{1}$	$1.00\times 10^{2}$	$1.38\times 10^{1}$
	avg	$2.90\times 10^{3}$	$3.26\times 10^{3}$	$3.19\times 10^{3}$	$3.07\times 10^{3}$	$2.95\times 10^{3}$	$3.08\times 10^{3}$	$3.32\times 10^{3}$	$3.04\times 10^{3}$
	median	$2.90\times 10^{3}$	$3.24\times 10^{3}$	$3.18\times 10^{3}$	$3.07\times 10^{3}$	$2.94\times 10^{3}$	$3.08\times 10^{3}$	$3.32\times 10^{3}$	$3.04\times 10^{3}$
	worst	$2.98\times 10^{3}$	$3.47\times 10^{3}$	$3.42\times 10^{3}$	$3.21\times 10^{3}$	$3.11\times 10^{3}$	$3.25\times 10^{3}$	$3.60\times 10^{3}$	$3.08\times 10^{3}$
	rank	1	7	6	4	2	5	8	3
F25	min	$2.88\times 10^{3}$	$3.09\times 10^{3}$	$2.90\times 10^{3}$	$2.88\times 10^{3}$	$2.95\times 10^{3}$	$2.88\times 10^{3}$	$3.09\times 10^{3}$	$2.90\times 10^{3}$
	std	$1.88\times 10^{1}$	$9.10\times 10^{1}$	$6.08\times 10^{1}$	$1.23\times 10^{2}$	$4.68\times 10^{1}$	$1.54\times 10^{1}$	$7.72\times 10^{1}$	$2.34\times 10^{1}$
	avg	$2.91\times 10^{3}$	$3.24\times 10^{3}$	$2.98\times 10^{3}$	$2.96\times 10^{3}$	$3.02\times 10^{3}$	$2.90\times 10^{3}$	$3.25\times 10^{3}$	$2.96\times 10^{3}$
	median	$2.90\times 10^{3}$	$3.22\times 10^{3}$	$2.97\times 10^{3}$	$2.91\times 10^{3}$	$3.02\times 10^{3}$	$2.89\times 10^{3}$	$3.25\times 10^{3}$	$2.96\times 10^{3}$
	worst	$2.96\times 10^{3}$	$3.49\times 10^{3}$	$3.19\times 10^{3}$	$3.38\times 10^{3}$	$3.13\times 10^{3}$	$2.94\times 10^{3}$	$3.40\times 10^{3}$	$3.01\times 10^{3}$
	rank	2	7	5	3	6	1	8	4

Notes: Boldface denotes the best performance among all compared algorithms for the corresponding statistic. The best result on F1 (min) is highlighted for the improved algorithm. All numerical results are reported in scientific notation, e.g., 1.04 × 10^2^.

**Table 7:** Results of the Wilcoxon rank-sum tests.

	WOA	DBO	PSO	GWO	SSA	HHO	ACO
F1	$3.02\times 10^{-11}$	$3.02\times 10^{-11}$	$3.34\times 10^{-11}$	$3.02\times 10^{-11}$	$1.22\times 10^{-2}$	$3.02\times 10^{-11}$	$3.02\times 10^{-11}$
F3	$3.02\times 10^{-11}$	$3.02\times 10^{-11}$	$3.02\times 10^{-11}$	$3.02\times 10^{-11}$	$3.02\times 10^{-11}$	$3.02\times 10^{-11}$	$3.02\times 10^{-11}$
F4	$3.02\times 10^{-11}$	$3.69\times 10^{-11}$	$4.46\times 10^{-4}$	$1.78\times 10^{-10}$	$1.49\times 10^{-1}$	$3.02\times 10^{-11}$	$4.86\times 10^{-3}$
F5	$3.02\times 10^{-11}$	$4.98\times 10^{-11}$	$4.64\times 10^{-5}$	$2.43\times 10^{-5}$	$5.49\times 10^{-11}$	$3.02\times 10^{-11}$	$3.02\times 10^{-11}$
F6	$3.02\times 10^{-11}$	$3.02\times 10^{-11}$	$5.19\times 10^{-7}$	$8.84\times 10^{-7}$	$3.02\times 10^{-11}$	$3.02\times 10^{-11}$	$9.06\times 10^{-8}$
F7	$3.02\times 10^{-11}$	$3.69\times 10^{-11}$	$8.99\times 10^{-11}$	$1.96\times 10^{-10}$	$3.02\times 10^{-11}$	$3.02\times 10^{-11}$	$3.02\times 10^{-11}$
F8	$3.02\times 10^{-11}$	$1.09\times 10^{-10}$	$1.24\times 10^{-3}$	$1.22\times 10^{-2}$	$1.33\times 10^{-10}$	$3.02\times 10^{-11}$	$3.02\times 10^{-11}$
F9	$3.02\times 10^{-11}$	$4.50\times 10^{-11}$	$3.77\times 10^{-4}$	$2.19\times 10^{-8}$	$3.02\times 10^{-11}$	$3.02\times 10^{-11}$	$3.16\times 10^{-10}$
F10	$2.37\times 10^{-10}$	$3.16\times 10^{-5}$	$6.10\times 10^{-3}$	$2.32\times 10^{-2}$	$7.17\times 10^{-1}$	$8.10\times 10^{-10}$	$3.02\times 10^{-11}$
F11	$3.02\times 10^{-11}$	$3.02\times 10^{-11}$	$1.04\times 10^{-4}$	$3.02\times 10^{-11}$	$1.73\times 10^{-7}$	$3.02\times 10^{-11}$	$3.02\times 10^{-11}$
F12	$3.02\times 10^{-11}$	$1.96\times 10^{-10}$	$3.92\times 10^{-2}$	$3.02\times 10^{-11}$	$7.01\times 10^{-2}$	$3.02\times 10^{-11}$	$6.12\times 10^{-10}$
F13	$3.02\times 10^{-11}$	$3.69\times 10^{-11}$	$2.50\times 10^{-3}$	$3.02\times 10^{-11}$	$2.01\times 10^{-4}$	$3.02\times 10^{-11}$	$3.95\times 10^{-1}$
F14	$3.02\times 10^{-11}$	$3.02\times 10^{-11}$	$4.50\times 10^{-11}$	$3.02\times 10^{-11}$	$3.02\times 10^{-11}$	$3.02\times 10^{-11}$	$3.02\times 10^{-11}$
F15	$3.02\times 10^{-11}$	$2.61\times 10^{-10}$	$6.31\times 10^{-1}$	$4.08\times 10^{-11}$	$2.42\times 10^{-2}$	$7.39\times 10^{-11}$	$8.66\times 10^{-5}$
F16	$3.34\times 10^{-11}$	$1.17\times 10^{-9}$	$9.35\times 10^{-1}$	$7.06\times 10^{-1}$	$2.13\times 10^{-4}$	$3.02\times 10^{-11}$	$5.49\times 10^{-11}$
F17	$3.16\times 10^{-10}$	$4.08\times 10^{-11}$	$4.06\times 10^{-2}$	$4.83\times 10^{-1}$	$3.01\times 10^{-7}$	$2.87\times 10^{-10}$	$8.10\times 10^{-10}$
F18	$4.08\times 10^{-11}$	$2.37\times 10^{-10}$	$3.16\times 10^{-10}$	$1.61\times 10^{-10}$	$1.25\times 10^{-7}$	$6.70\times 10^{-11}$	$3.02\times 10^{-11}$
F19	$3.02\times 10^{-11}$	$7.69\times 10^{-8}$	$5.20\times 10^{-1}$	$3.02\times 10^{-11}$	$2.97\times 10^{-1}$	$3.02\times 10^{-11}$	$3.18\times 10^{-4}$
F20	$4.69\times 10^{-8}$	$2.27\times 10^{-3}$	$4.12\times 10^{-1}$	$3.03\times 10^{-2}$	$9.21\times 10^{-5}$	$2.28\times 10^{-5}$	$1.41\times 10^{-4}$
F21	$3.02\times 10^{-11}$	$3.02\times 10^{-11}$	$3.82\times 10^{-10}$	$9.26\times 10^{-9}$	$3.02\times 10^{-11}$	$3.02\times 10^{-11}$	$3.02\times 10^{-11}$
F22	$4.98\times 10^{-11}$	$5.00\times 10^{-9}$	$2.02\times 10^{-8}$	$1.85\times 10^{-8}$	$1.01\times 10^{-8}$	$7.38\times 10^{-10}$	$9.92\times 10^{-11}$
F23	$3.02\times 10^{-11}$	$3.02\times 10^{-11}$	$3.02\times 10^{-11}$	$1.49\times 10^{-6}$	$3.02\times 10^{-11}$	$3.02\times 10^{-11}$	$3.02\times 10^{-11}$
F24	$3.02\times 10^{-11}$	$3.02\times 10^{-11}$	$3.69\times 10^{-11}$	$7.74\times 10^{-6}$	$6.70\times 10^{-11}$	$3.02\times 10^{-11}$	$3.02\times 10^{-11}$
F25	$3.02\times 10^{-11}$	$6.52\times 10^{-9}$	$2.34\times 10^{-1}$	$3.34\times 10^{-11}$	$1.56\times 10^{-2}$	$3.02\times 10^{-11}$	$2.44\times 10^{-9}$
F26	$7.39\times 10^{-11}$	$8.89\times 10^{-10}$	$5.32\times 10^{-3}$	$4.98\times 10^{-4}$	$6.72\times 10^{-10}$	$4.50\times 10^{-11}$	$3.02\times 10^{-11}$
F27	$6.70\times 10^{-11}$	$8.48\times 10^{-9}$	$3.34\times 10^{-3}$	$1.00\times 10^{-3}$	$7.30\times 10^{-4}$	$3.34\times 10^{-11}$	$4.11\times 10^{-7}$
F28	$3.02\times 10^{-11}$	$3.02\times 10^{-11}$	$1.09\times 10^{-5}$	$3.02\times 10^{-11}$	$1.02\times 10^{-1}$	$3.02\times 10^{-11}$	$3.02\times 10^{-11}$
F29	$3.02\times 10^{-11}$	$2.87\times 10^{-10}$	$6.31\times 10^{-1}$	$6.38\times 10^{-3}$	$9.53\times 10^{-7}$	$3.02\times 10^{-11}$	$1.36\times 10^{-7}$
F30	$3.02\times 10^{-11}$	$3.02\times 10^{-11}$	$2.19\times 10^{-8}$	$3.02\times 10^{-11}$	$1.71\times 10^{-1}$	$3.02\times 10^{-11}$	$1.07\times 10^{-7}$
count	0	0	6	2	6	0	1

**Table 8:** Mean and standard deviation of single-UAV path-planning fitness.

Algorithm	M1-C1	M1-C2	M2-C1	M2-C2	Average Ranking
IWOA	9305.32 ± 30.02	9571.63 ± 23.28	6071.74 ± 87.56	6286.26 ± 363.45	1.25
WOA	9307.98 ± 25.22	9575.59 ± 19.34	7249.49 ± 657.99	7146.20 ± 928.24	3.50
DBO	9381.71 ± 313.74	9836.08 ± 797.92	6756.30 ± 717.95	7088.76 ± 719.59	4.00
PSO	9832.85 ± 287.82	10,126.92 ± 569.29	7115.08 ± 512.73	7722.60 ± 939.64	5.50
GWO	9284.36 ± 8.63	9646.29 ± 698.10	6456.96 ± 381.49	6492.36 ± 458.14	2.25
SSA	9945.16 ± 533.19	11,324.70 ± 754.67	7529.39 ± 784.19	8675.83 ± 1509.07	7.25
HHO	9323.19 ± 22.30	9585.18 ± 2.93	8732.01 ± 579.21	9239.54 ± 1312.34	5.75
ACO	9458.03 ± 54.09	11,079.30 ± 1286.41	8281.58 ± 325.28	8386.21 ± 618.53	6.50

**Table 9:** Feasibility-priority comparison over 30 paired 500-iteration runs per scenario. Both algorithms were feasible in 30/30 runs. Values are the implemented composite objective among feasible final solutions; lower is better. Positive rank-biserial values favor IWOA-FP.

Scene	IWOA-FP Mean ± SD	IWOA-FP Median [IQR]	WOA-FP Mean ± SD	WOA-FP Median [IQR]	$p_{\mathrm{Holm}}$	Rank-Biserial
M3-C1	8194.1 ± 743.2	8452.4 [7354.2, 8859.1]	8201.0 ± 570.3	8348.5 [8148.1, 8614.5]	0.9836	0.006
M3-C2	12,564.0 ± 1562.0	12,631.2 [11,475.1, 13,599.6]	9550.8 ± 382.5	9445.6 [9335.0, 9724.1]	$8.09\times 10^{-6}$	−0.996
M4-C1	0.479 ± 0.046	0.486 [0.442, 0.512]	0.638 ± 0.065	0.631 [0.595, 0.672]	$8.09\times 10^{-6}$	0.996
M4-C2	498.420 ± 4.142	497.354 [495.102, 500.166]	506.008 ± 3.082	506.154 [504.743, 507.475]	$1.78\times 10^{-5}$	0.931

SD: standard deviation; IQR: interquartile range.

## Data Availability

The MATLAB scripts and scenario files used for the revision experiments are available from the corresponding author, no external dataset was analyzed in this study.

## Abbreviations

UAV	Unmanned Aerial Vehicle
3-D	Three-Dimensional
A*	A-star algorithm
PSO	Particle Swarm Optimization
GA	Genetic Algorithm
DE	Differential Evolution
OBST	Obstacle
SQP	Sequential Quadratic Programming
NMPC	Non-linear Model Predictive Control
CPU	Central Processing Unit
RMSE	Root Mean Square Error
MAPE	Mean Absolute Percentage Error
FPS	Frames Per Second
